# Supplementary material for: Hormone replacement therapy for postmenopausal atherosclerosis is offset by late age iron deposition
Source: eLife. 2023 Aug 10;12:e80494. doi: 10.7554/eLife.80494 (PMC10414966; doi:10.7554/eLife.80494)
Supplement: Figure 1—source data 1. [file elife-80494-fig1-data1.zip › Fig1/Fig1A/Fig1A.pptx]

## Slide 1
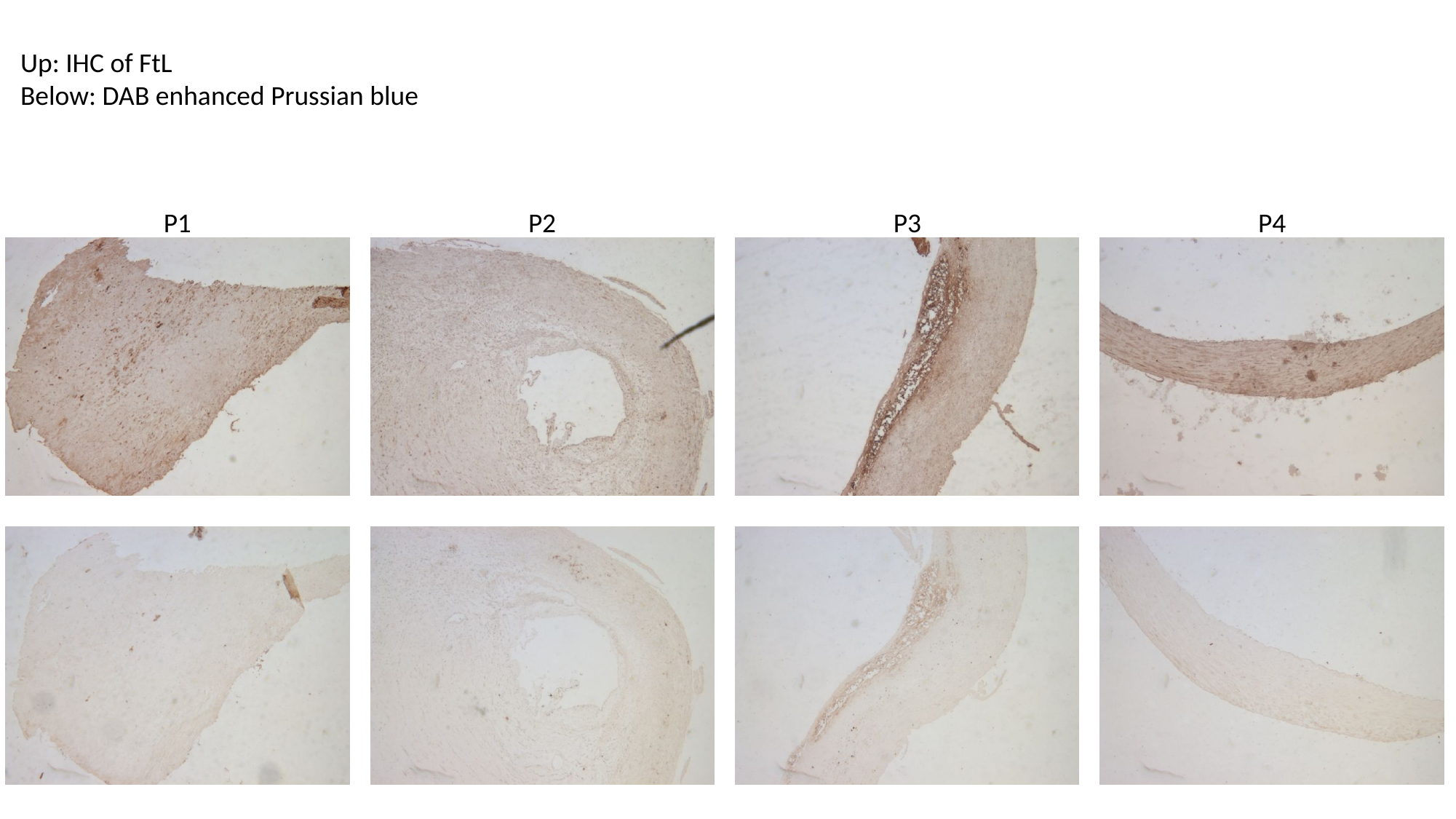

Up: IHC of FtL
Below: DAB enhanced Prussian blue
P1
P2
P3
P4

## Slide 2
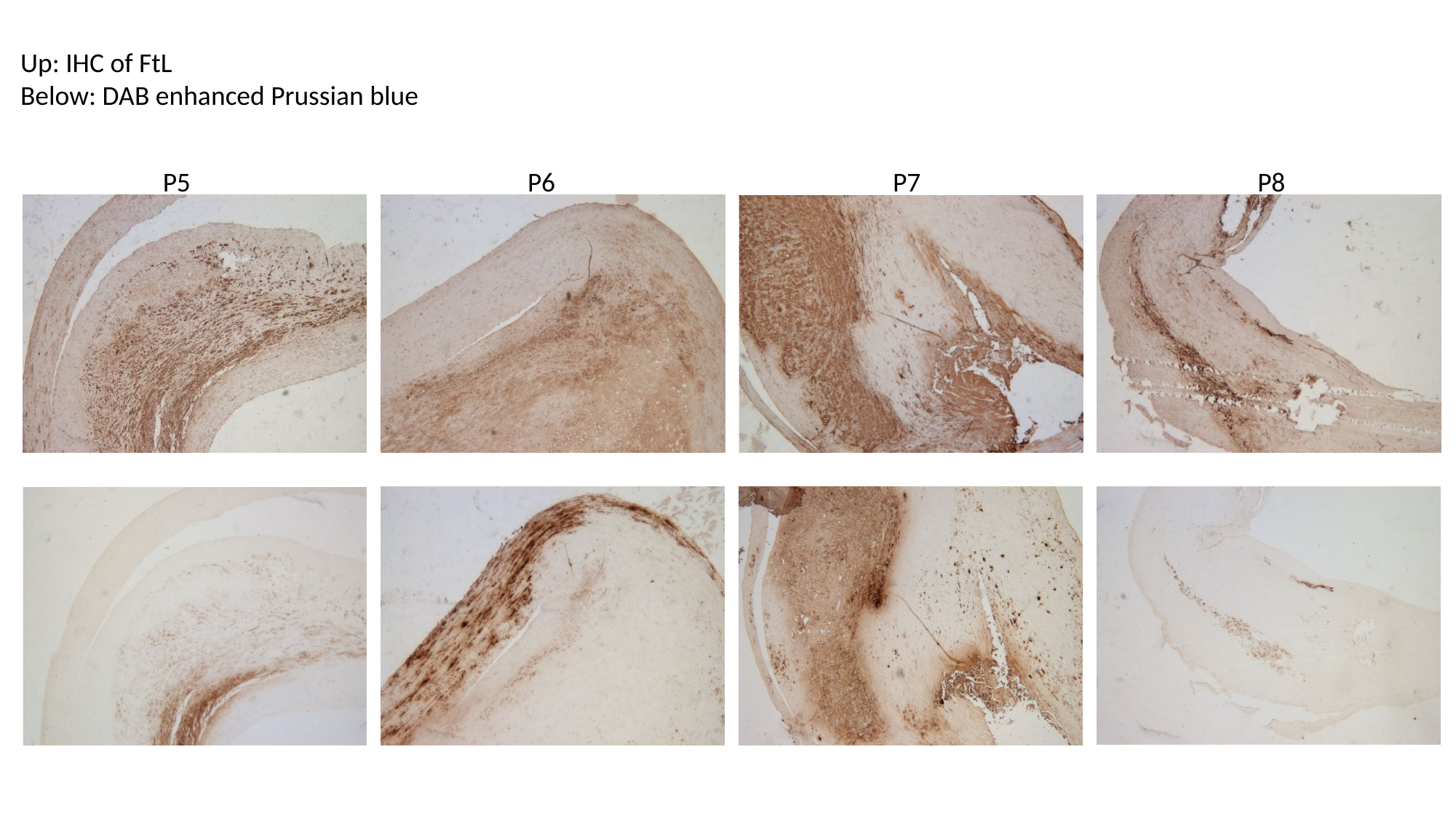

Up: IHC of FtL
Below: DAB enhanced Prussian blue
P5
P6
P7
P8
