# Supplementary figures and images for: Hormone replacement therapy for postmenopausal atherosclerosis is offset by late age iron deposition (part 1 of 2)
Source: eLife. 2023 Aug 10;12:e80494. doi: 10.7554/eLife.80494 (PMC10414966; doi:10.7554/eLife.80494)

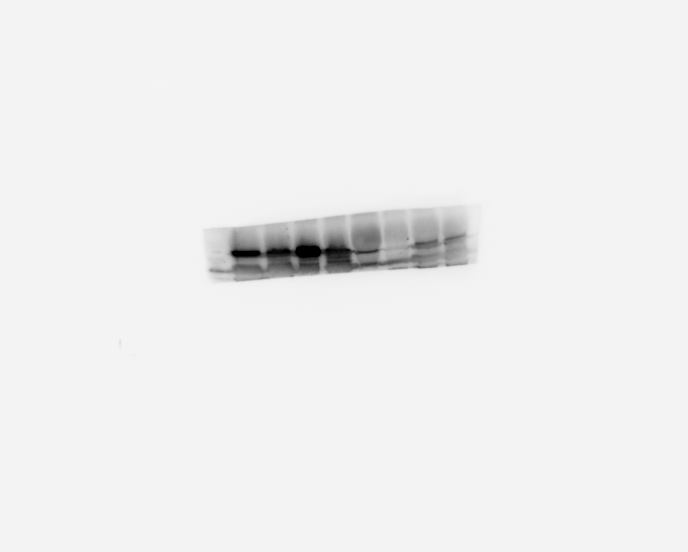

Supplement: Figure 1—source data 1. [file elife-80494-fig1-data1.zip › Fig1/Fig1B/ERa┴/60.tif]

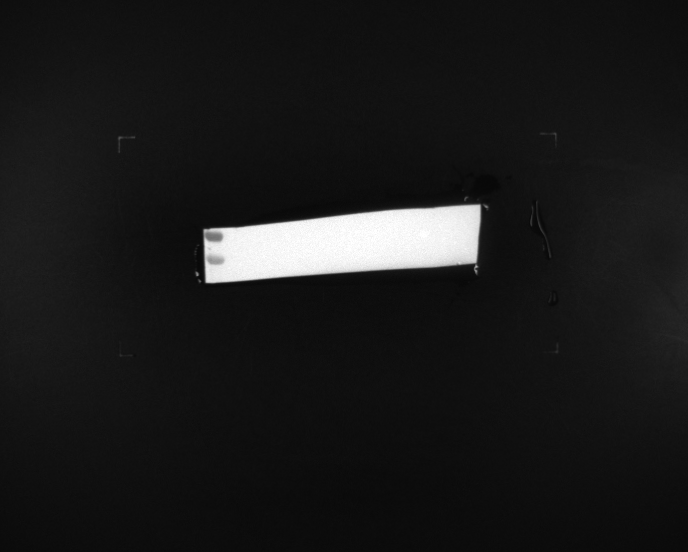

Supplement: Figure 1—source data 1. [file elife-80494-fig1-data1.zip › Fig1/Fig1B/ERa┴/marker.tif]

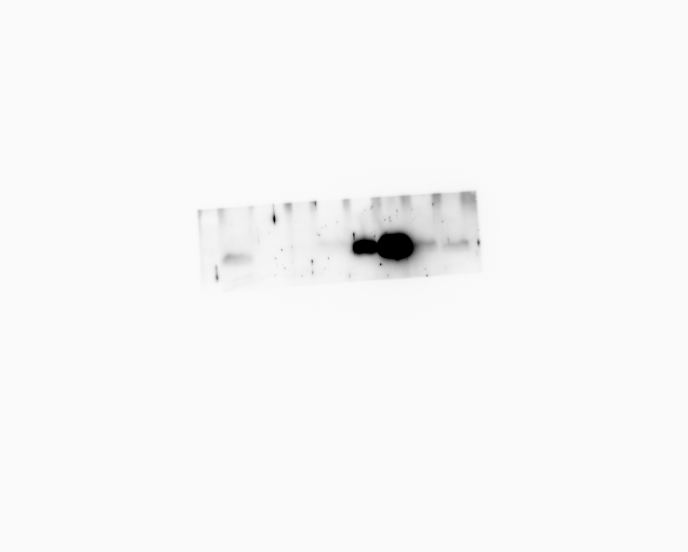

Supplement: Figure 1—source data 1. [file elife-80494-fig1-data1.zip › Fig1/Fig1B/Ftl/10.tif]

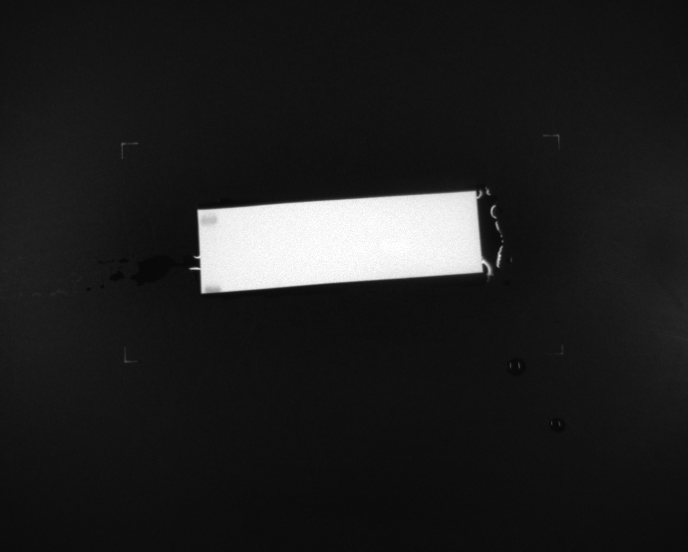

Supplement: Figure 1—source data 1. [file elife-80494-fig1-data1.zip › Fig1/Fig1B/Ftl/mk.tif]

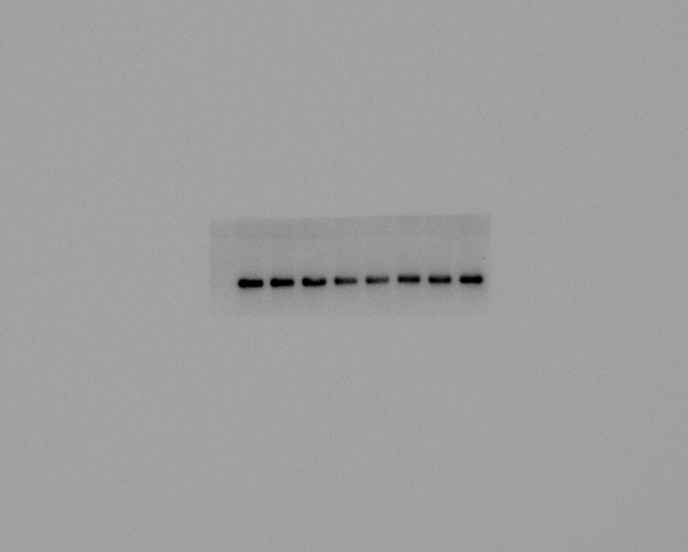

Supplement: Figure 1—source data 1. [file elife-80494-fig1-data1.zip › Fig1/Fig1B/GAPDH/10.tif]

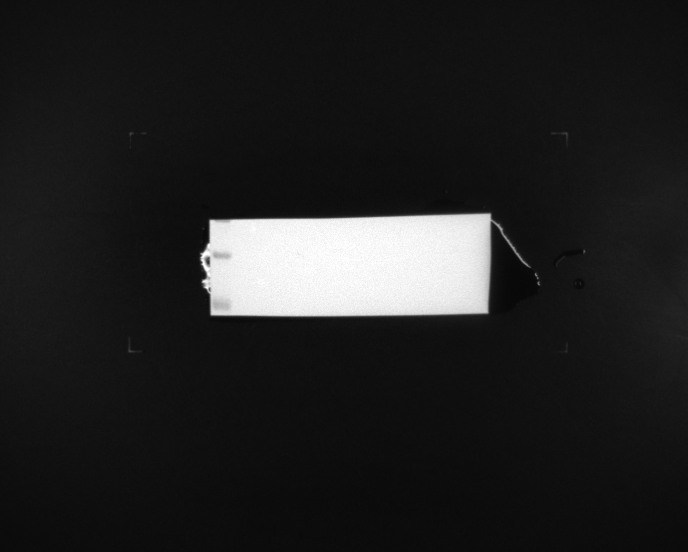

Supplement: Figure 1—source data 1. [file elife-80494-fig1-data1.zip › Fig1/Fig1B/GAPDH/MARKER.tif]

## Slide 1
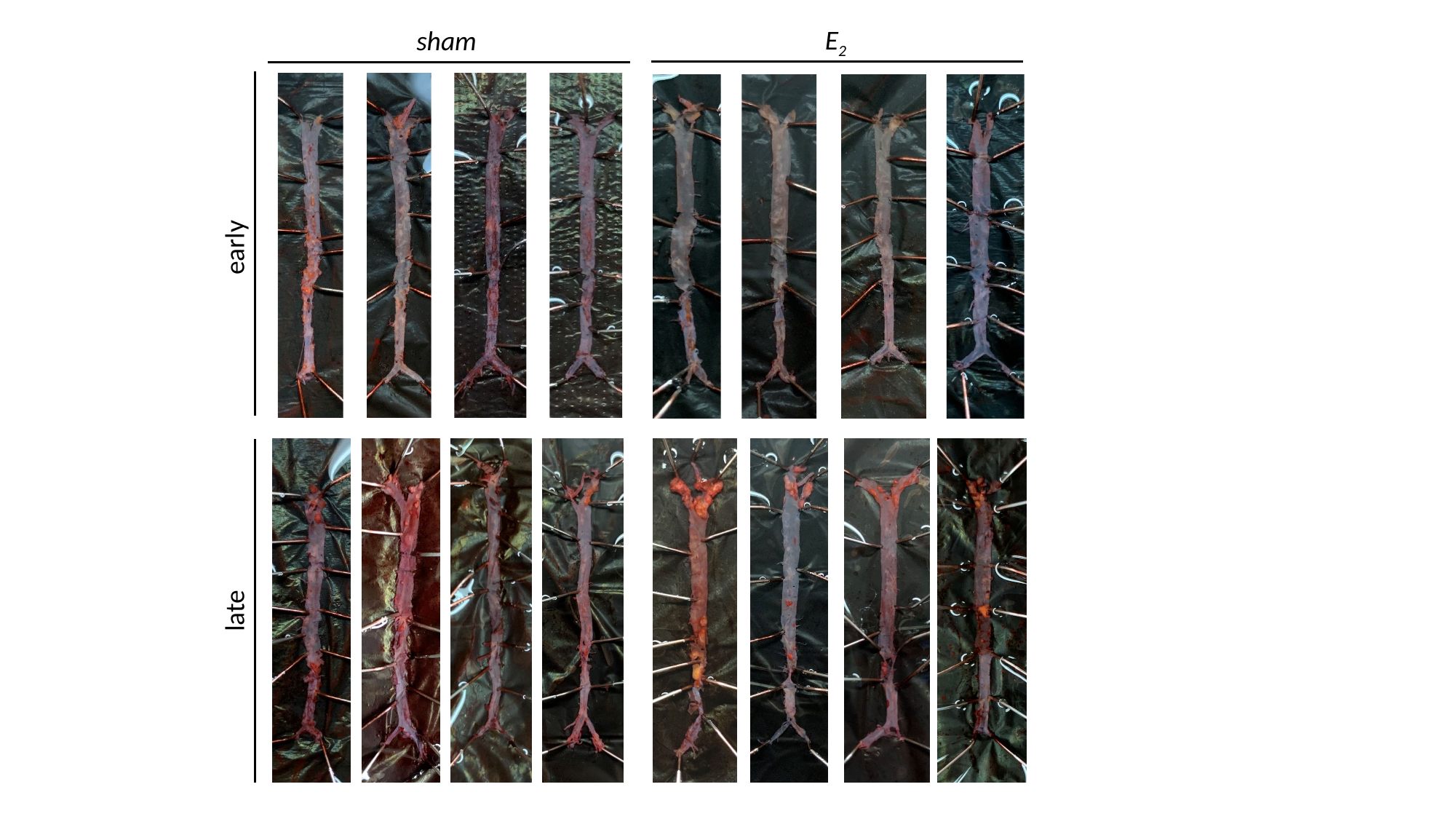

E2
sham
early
late

Supplement: Figure 2—source data 1. [file elife-80494-fig2-data1.zip › Fig2/Fig2B/Fig2B.pptx]

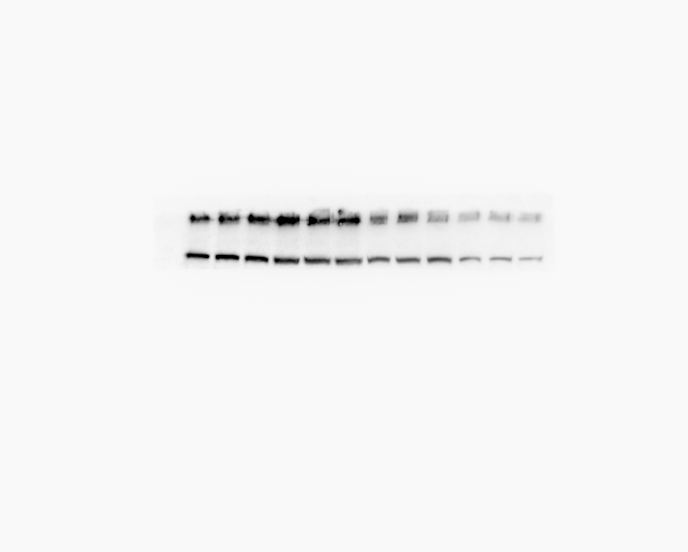

Supplement: Figure 2—source data 1. [file elife-80494-fig2-data1.zip › Fig2/Fig2D/ABCA1/10.tif]

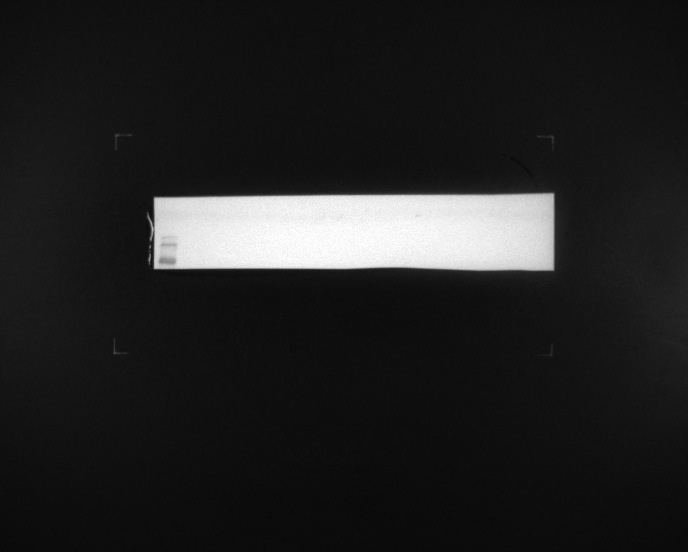

Supplement: Figure 2—source data 1. [file elife-80494-fig2-data1.zip › Fig2/Fig2D/ABCA1/MK.tif]

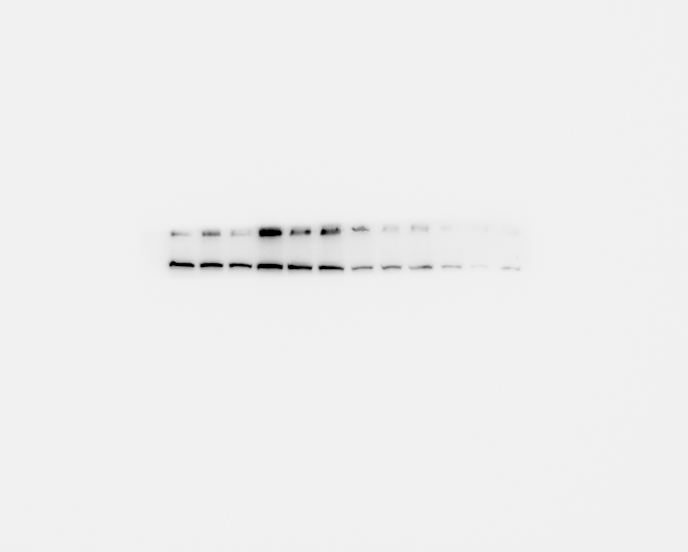

Supplement: Figure 2—source data 1. [file elife-80494-fig2-data1.zip › Fig2/Fig2D/eNOS/30.tif]

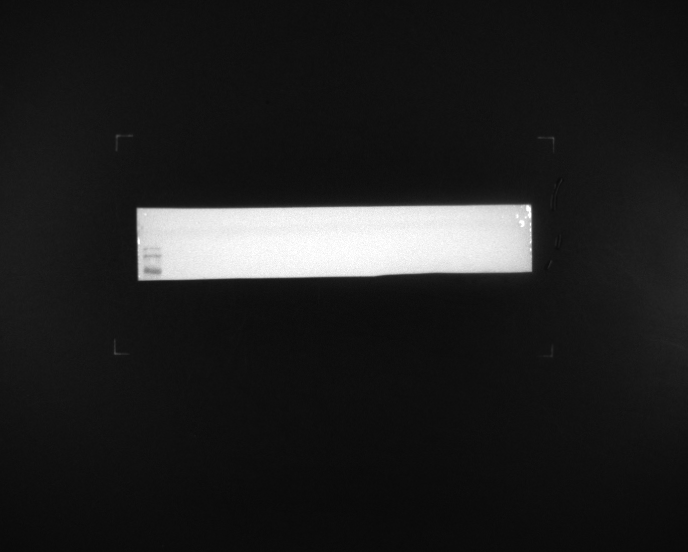

Supplement: Figure 2—source data 1. [file elife-80494-fig2-data1.zip › Fig2/Fig2D/eNOS/mk.tif]

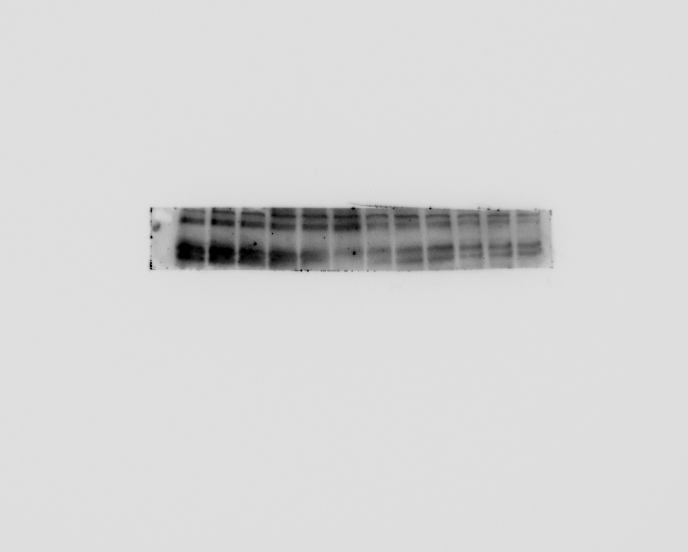

Supplement: Figure 2—source data 1. [file elife-80494-fig2-data1.zip › Fig2/Fig2D/ERa┴/60.tif]

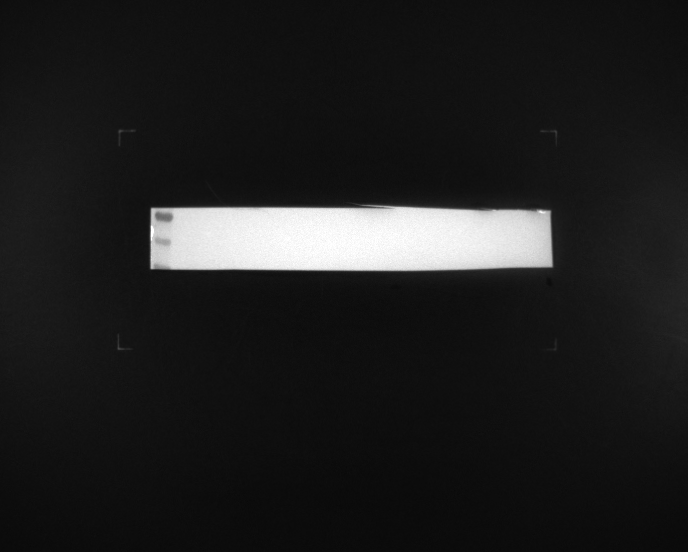

Supplement: Figure 2—source data 1. [file elife-80494-fig2-data1.zip › Fig2/Fig2D/ERa┴/MK.tif]

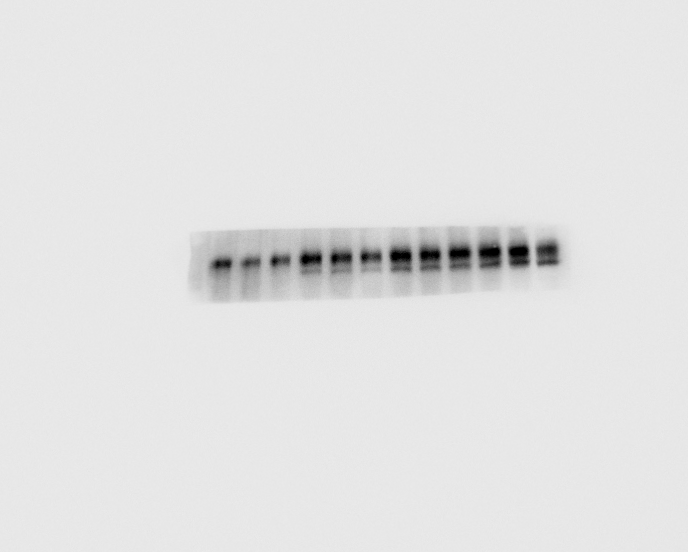

Supplement: Figure 2—source data 1. [file elife-80494-fig2-data1.zip › Fig2/Fig2D/Fph/1.tif]

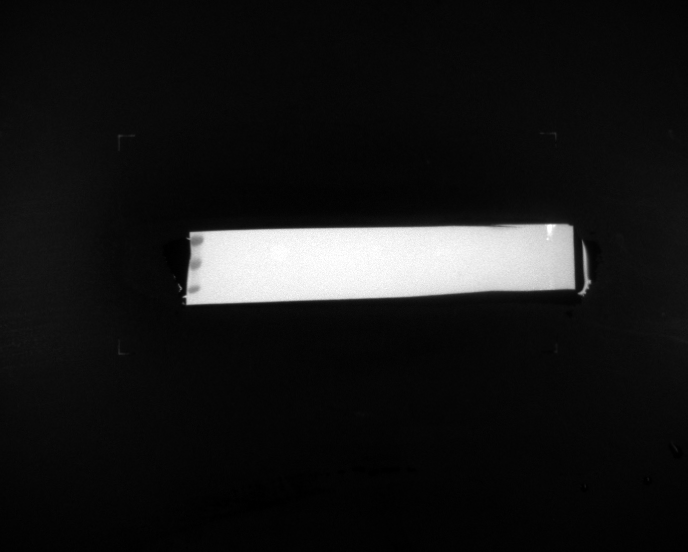

Supplement: Figure 2—source data 1. [file elife-80494-fig2-data1.zip › Fig2/Fig2D/Fph/mk.tif]

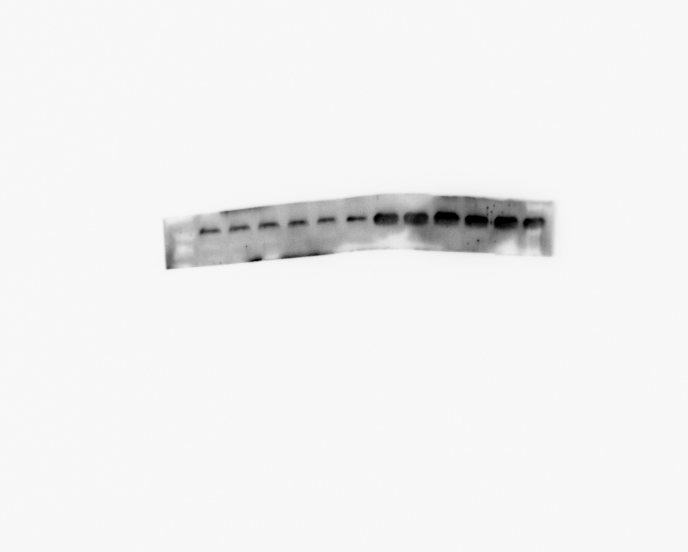

Supplement: Figure 2—source data 1. [file elife-80494-fig2-data1.zip › Fig2/Fig2D/Ftl/1.tif]

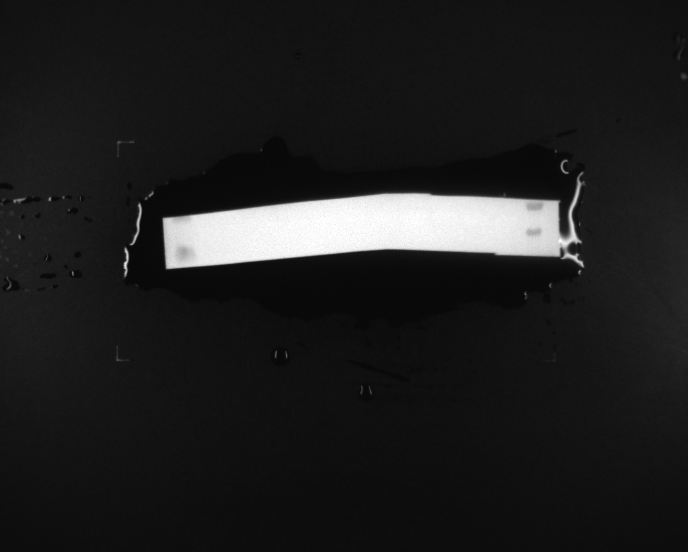

Supplement: Figure 2—source data 1. [file elife-80494-fig2-data1.zip › Fig2/Fig2D/Ftl/mk.tif]

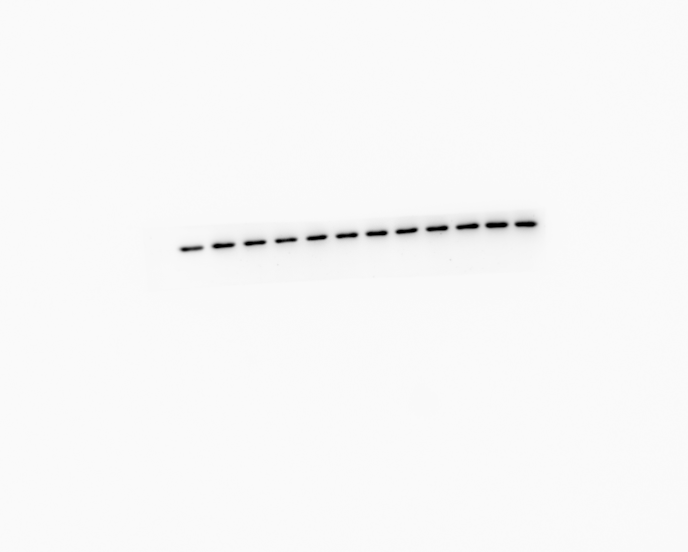

Supplement: Figure 2—source data 1. [file elife-80494-fig2-data1.zip › Fig2/Fig2D/GAPDH/10.tif]

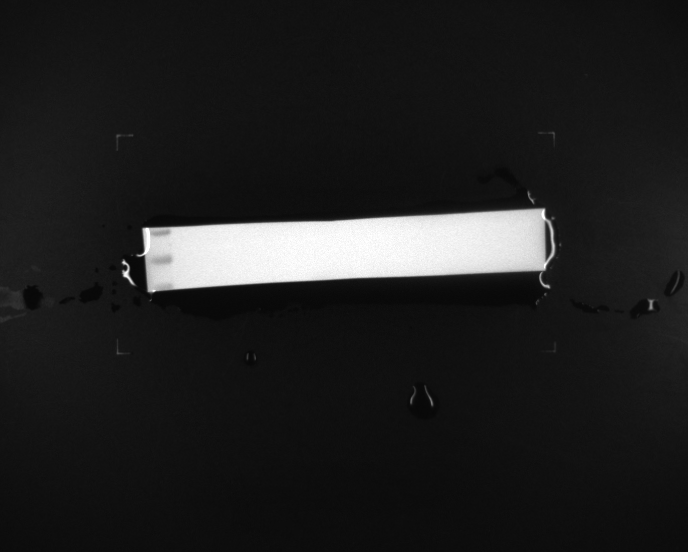

Supplement: Figure 2—source data 1. [file elife-80494-fig2-data1.zip › Fig2/Fig2D/GAPDH/mk.tif]

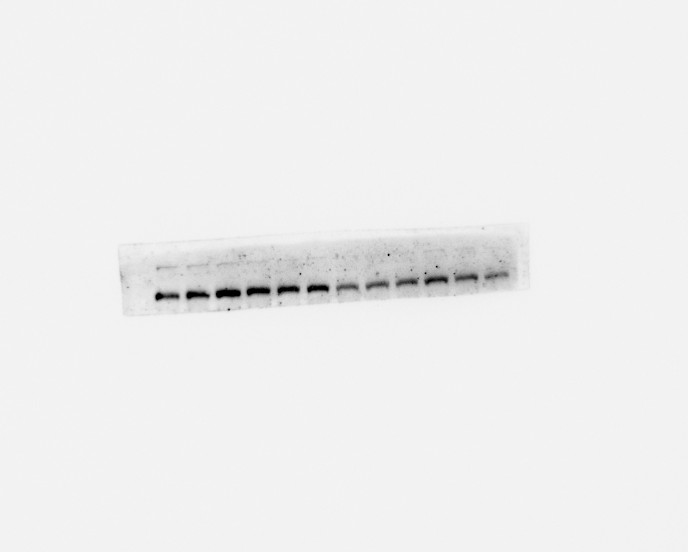

Supplement: Figure 2—source data 1. [file elife-80494-fig2-data1.zip › Fig2/Fig2D/TfR1/10.tif]

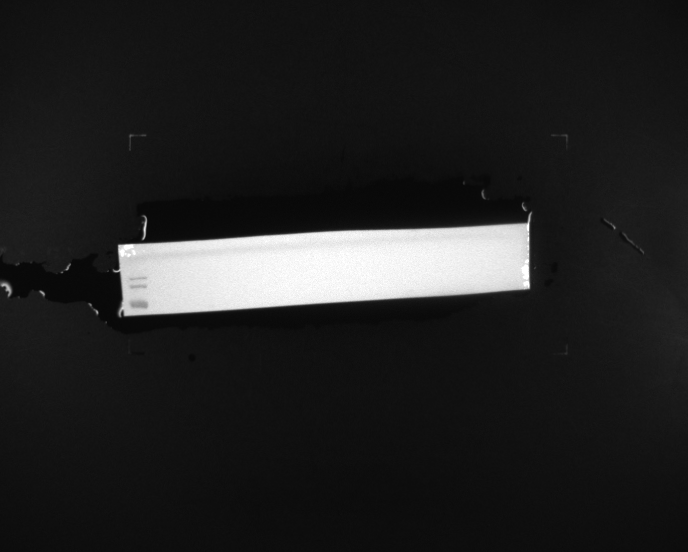

Supplement: Figure 2—source data 1. [file elife-80494-fig2-data1.zip › Fig2/Fig2D/TfR1/MK.tif]

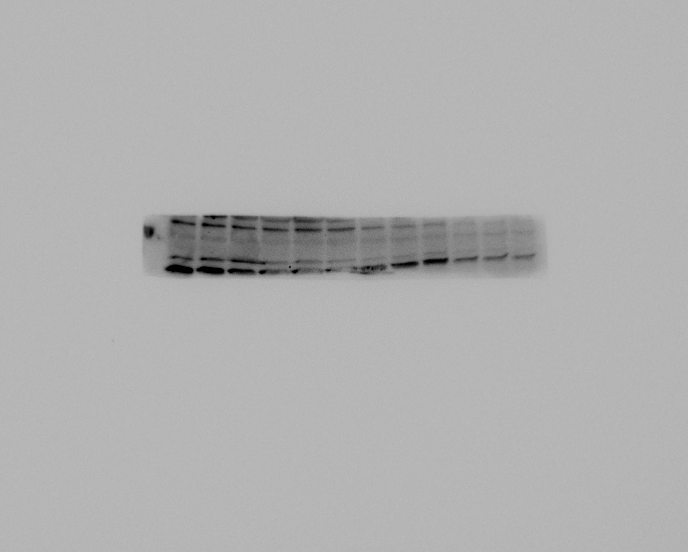

Supplement: Figure 2—source data 1. [file elife-80494-fig2-data1.zip › Fig2/Fig2D/VEGF/30.tif]

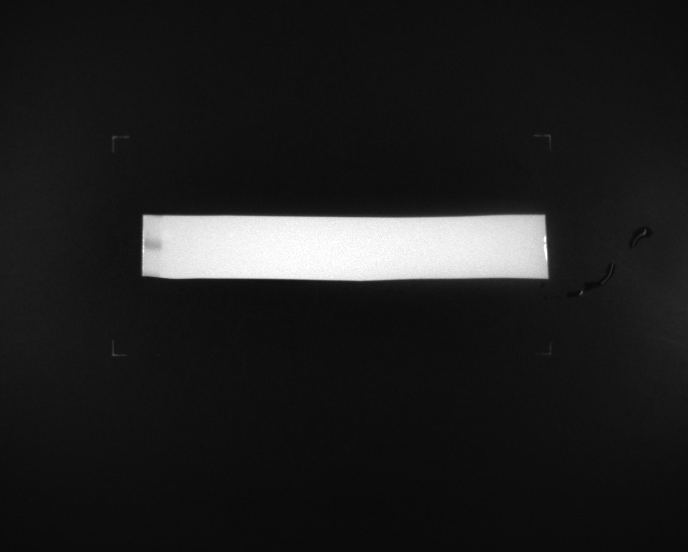

Supplement: Figure 2—source data 1. [file elife-80494-fig2-data1.zip › Fig2/Fig2D/VEGF/MK.tif]

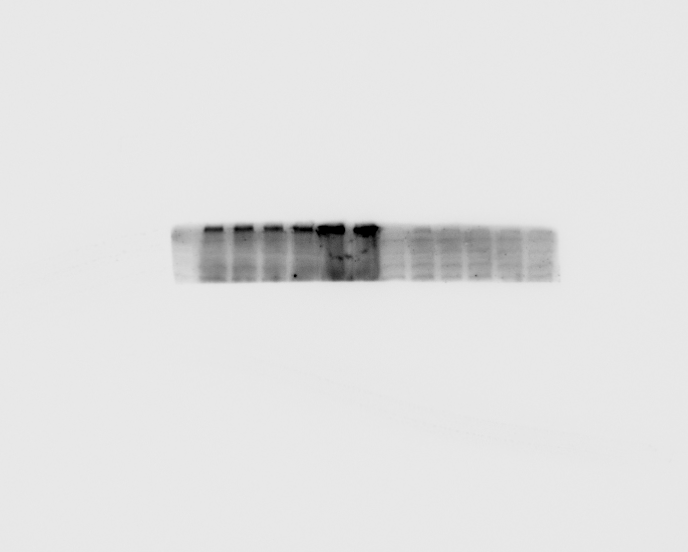

Supplement: Figure 2—source data 1. [file elife-80494-fig2-data1.zip › Fig2/Fig2E/ABCA1/30.tif]

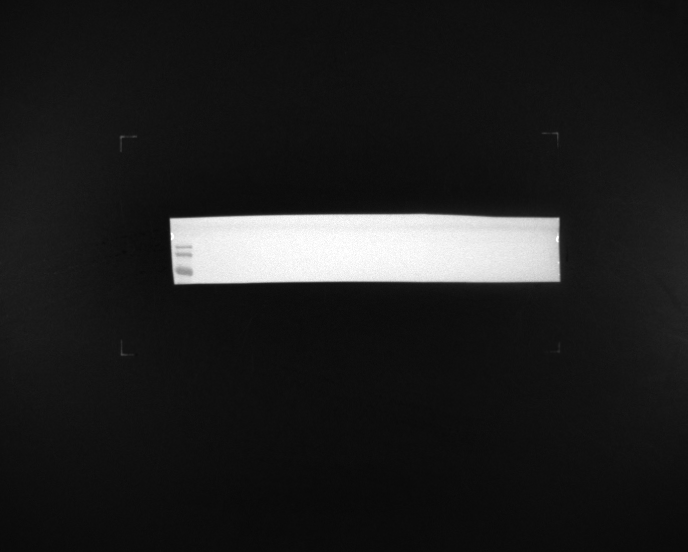

Supplement: Figure 2—source data 1. [file elife-80494-fig2-data1.zip › Fig2/Fig2E/ABCA1/MK.tif]

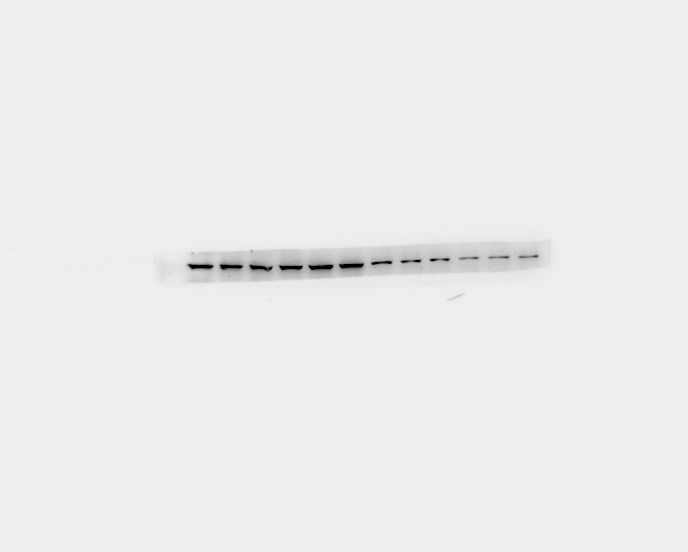

Supplement: Figure 2—source data 1. [file elife-80494-fig2-data1.zip › Fig2/Fig2E/ERa┴/30.tif]

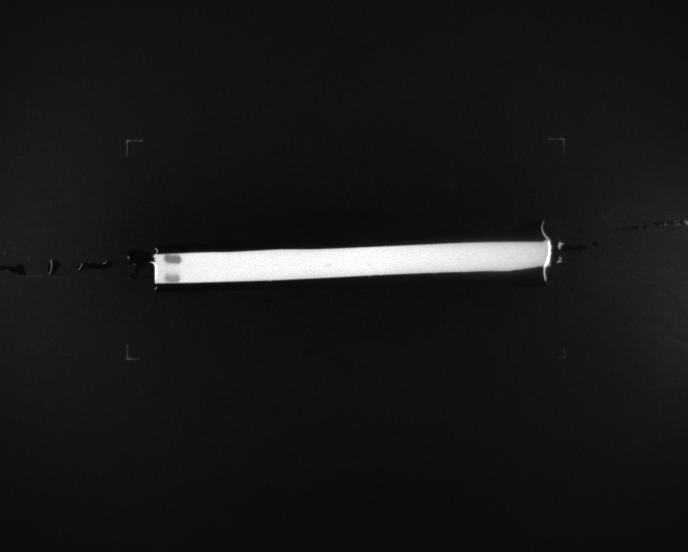

Supplement: Figure 2—source data 1. [file elife-80494-fig2-data1.zip › Fig2/Fig2E/ERa┴/MK.tif]

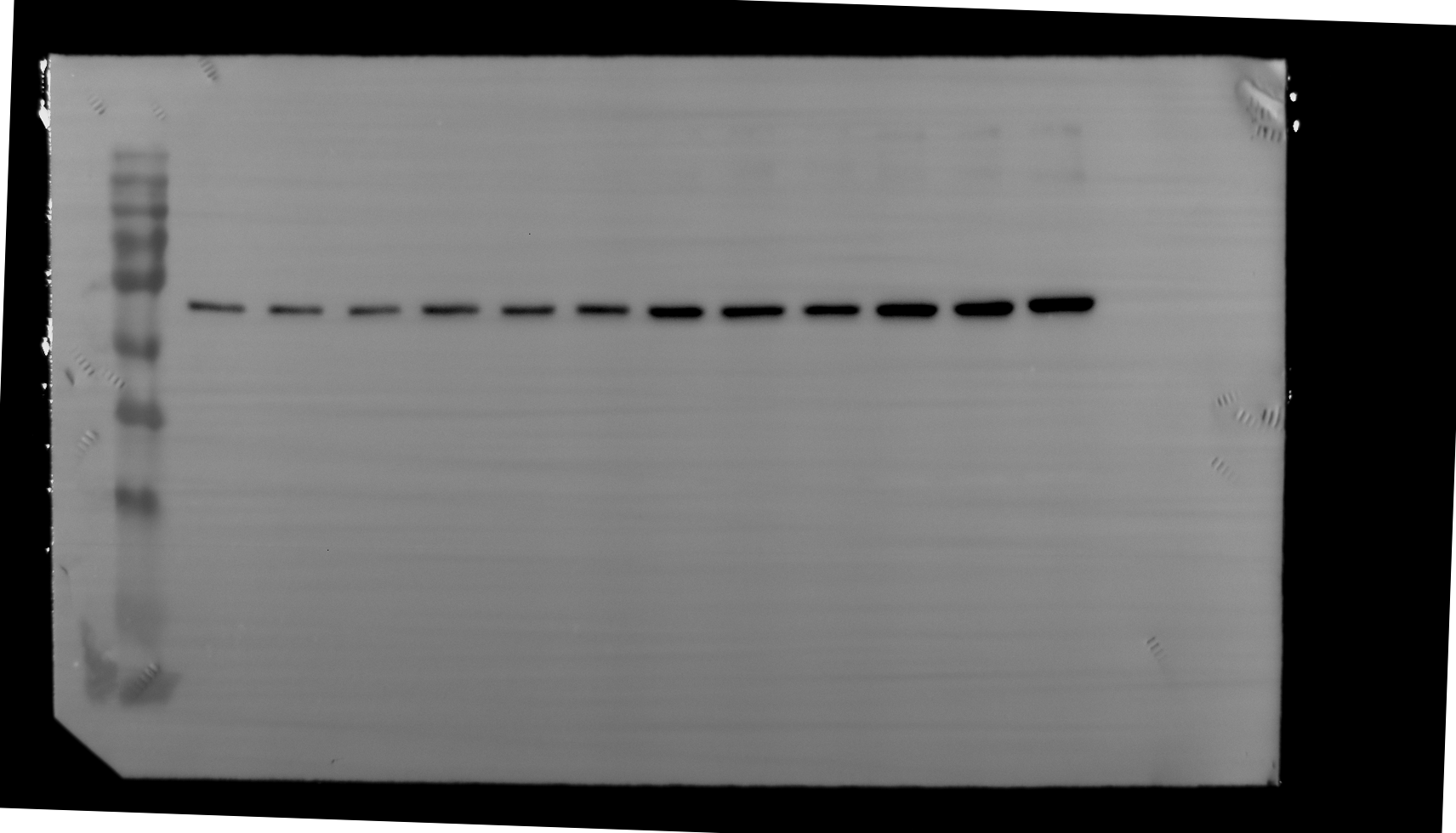

Supplement: Figure 2—source data 1. [file elife-80494-fig2-data1.zip › Fig2/Fig2E/FPN1.tif]

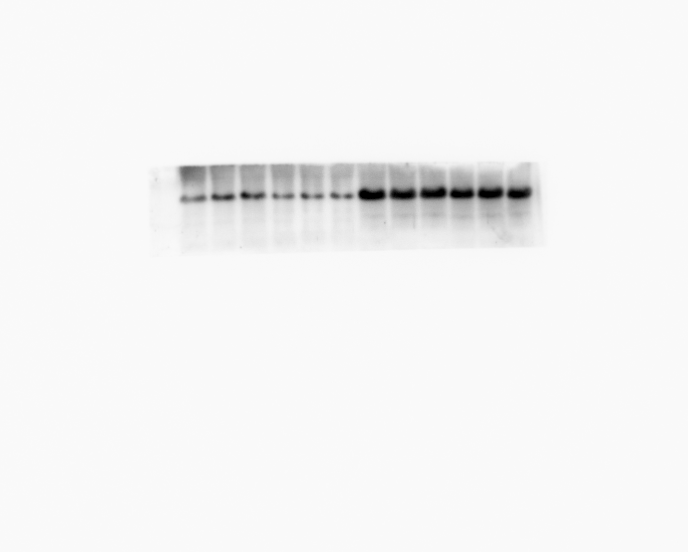

Supplement: Figure 2—source data 1. [file elife-80494-fig2-data1.zip › Fig2/Fig2E/Ftl/1.tif]

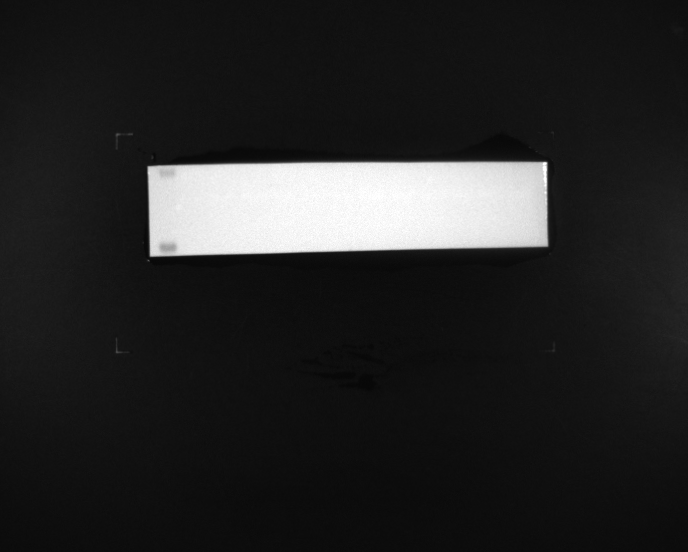

Supplement: Figure 2—source data 1. [file elife-80494-fig2-data1.zip › Fig2/Fig2E/Ftl/mk.tif]

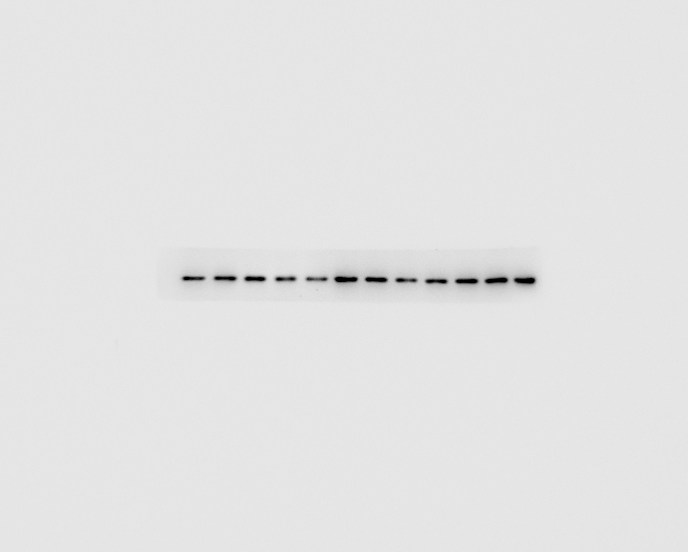

Supplement: Figure 2—source data 1. [file elife-80494-fig2-data1.zip › Fig2/Fig2E/GAPDH/3.tif]

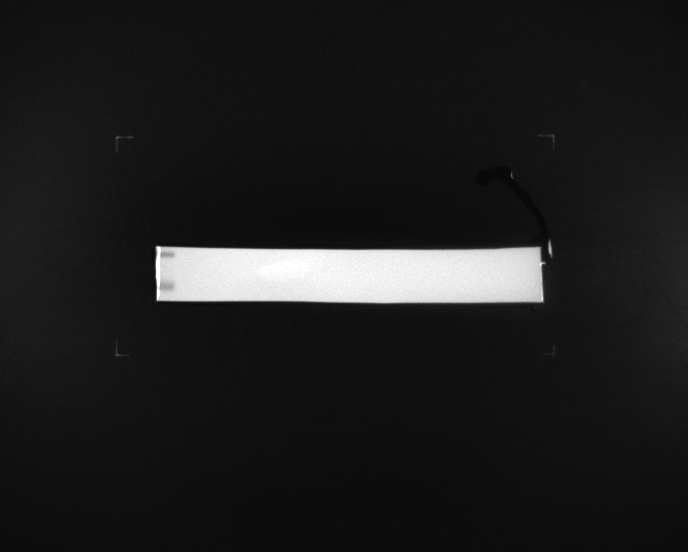

Supplement: Figure 2—source data 1. [file elife-80494-fig2-data1.zip › Fig2/Fig2E/GAPDH/mk.tif]

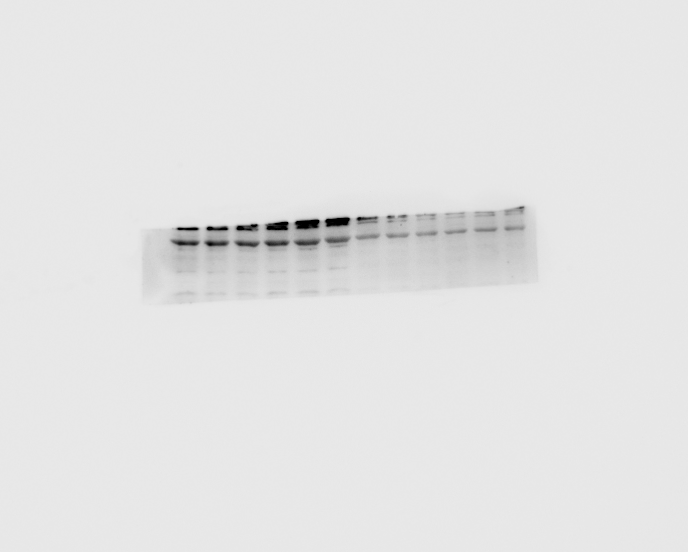

Supplement: Figure 2—source data 1. [file elife-80494-fig2-data1.zip › Fig2/Fig2E/VEGF/30.tif]

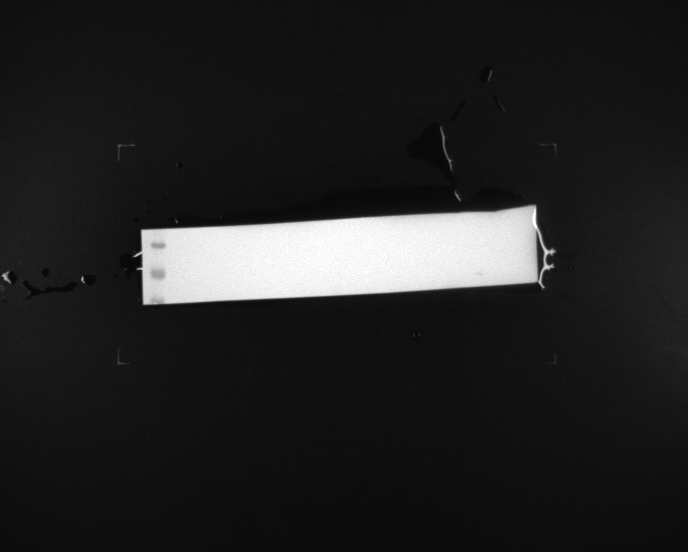

Supplement: Figure 2—source data 1. [file elife-80494-fig2-data1.zip › Fig2/Fig2E/VEGF/MK.tif]

## Slide 1
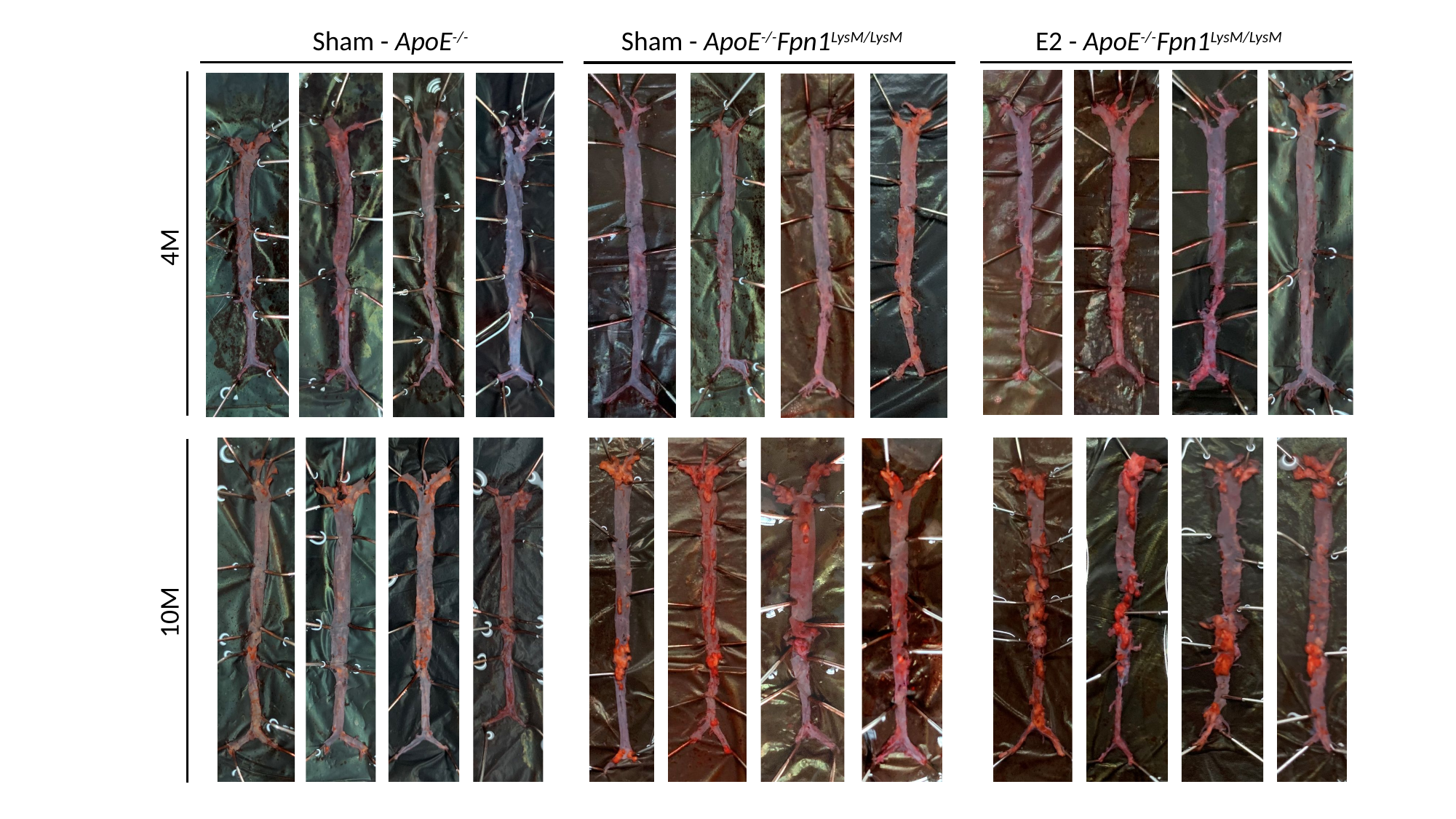

Sham - ApoE-/-
E2 - ApoE-/-Fpn1LysM/LysM
Sham - ApoE-/-Fpn1LysM/LysM
4M
10M

Supplement: Figure 3—source data 1. [file elife-80494-fig3-data1.zip › Fig3/Fig3B/Fig3B.pptx]

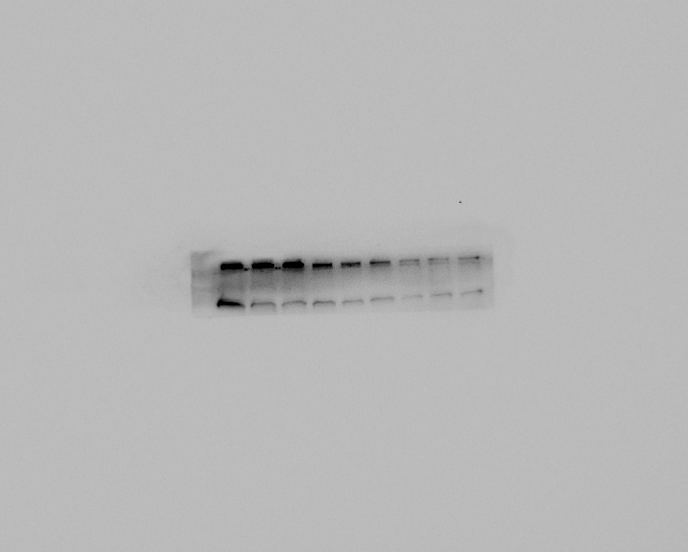

Supplement: Figure 3—source data 1. [file elife-80494-fig3-data1.zip › Fig3/Fig3E/ABCA1/30.tif]

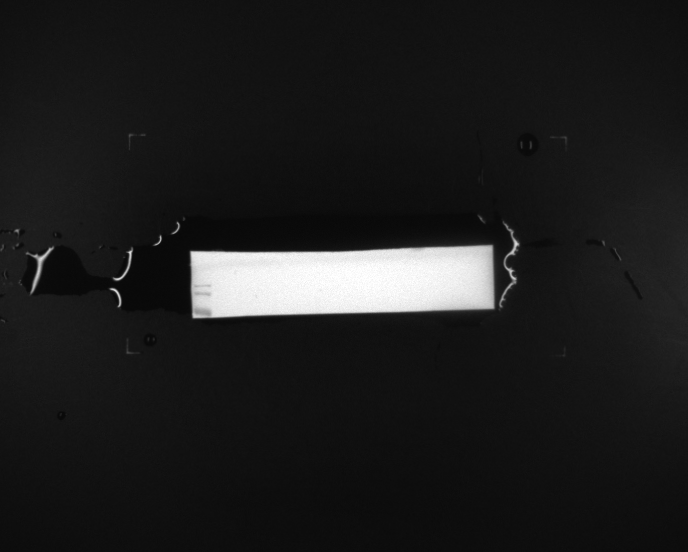

Supplement: Figure 3—source data 1. [file elife-80494-fig3-data1.zip › Fig3/Fig3E/ABCA1/mk.tif]

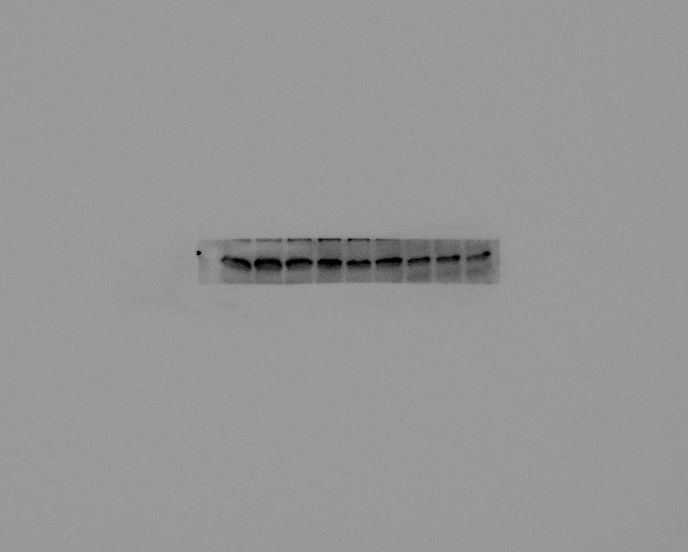

Supplement: Figure 3—source data 1. [file elife-80494-fig3-data1.zip › Fig3/Fig3E/ERa┴/10.tif]

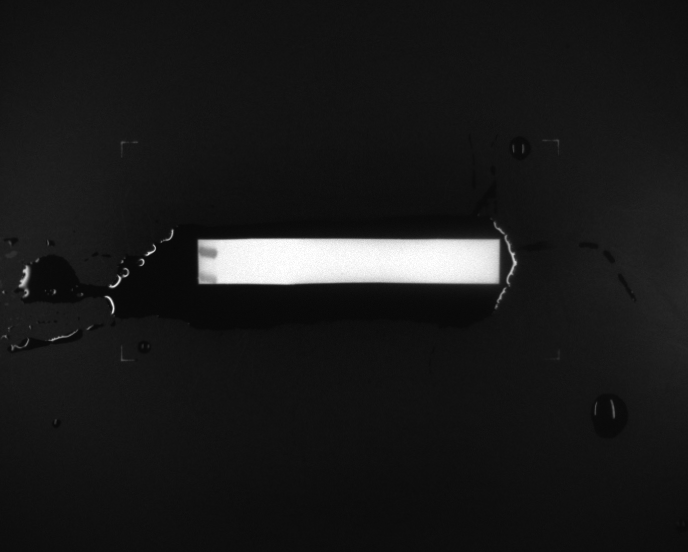

Supplement: Figure 3—source data 1. [file elife-80494-fig3-data1.zip › Fig3/Fig3E/ERa┴/m.tif]

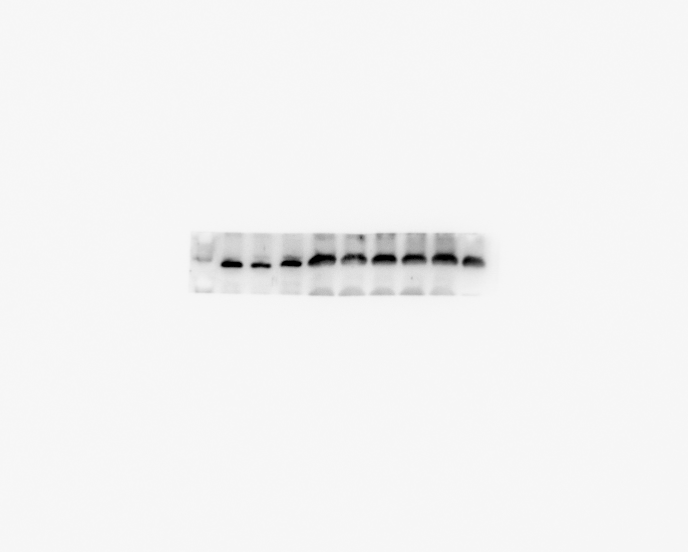

Supplement: Figure 3—source data 1. [file elife-80494-fig3-data1.zip › Fig3/Fig3E/Ftl/1.tif]

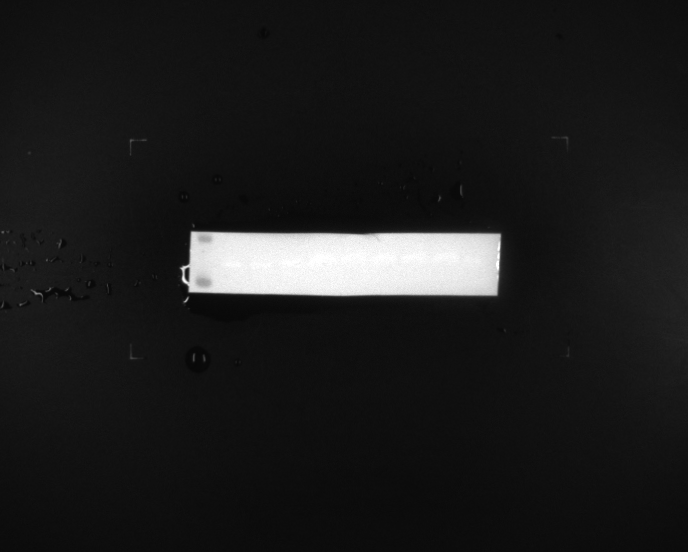

Supplement: Figure 3—source data 1. [file elife-80494-fig3-data1.zip › Fig3/Fig3E/Ftl/mk.tif]

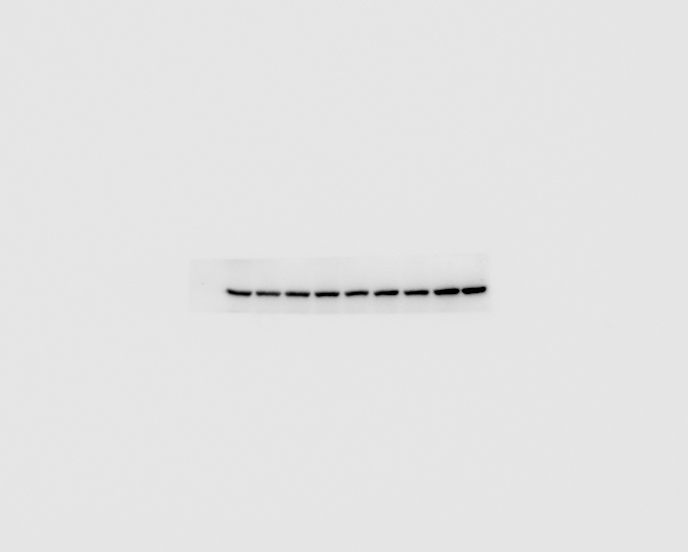

Supplement: Figure 3—source data 1. [file elife-80494-fig3-data1.zip › Fig3/Fig3E/GAPDH/10.tif]

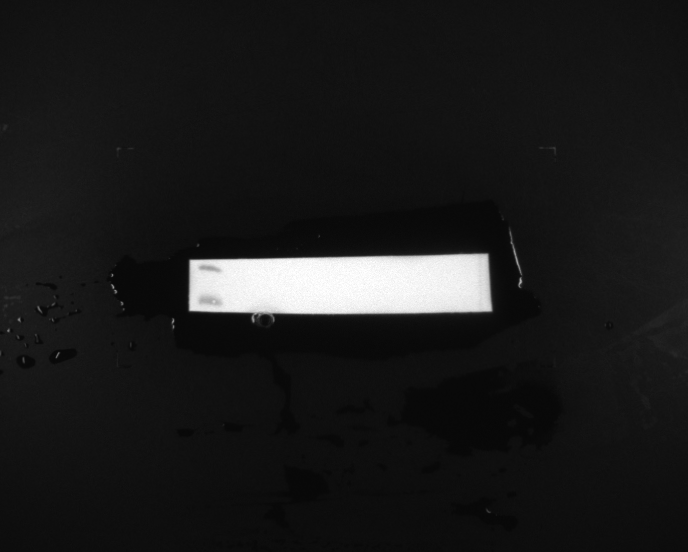

Supplement: Figure 3—source data 1. [file elife-80494-fig3-data1.zip › Fig3/Fig3E/GAPDH/MK.tif]

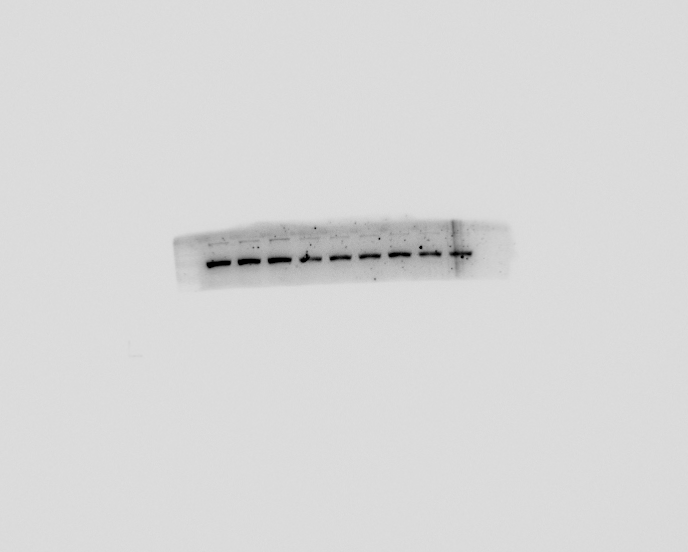

Supplement: Figure 3—source data 1. [file elife-80494-fig3-data1.zip › Fig3/Fig3E/Tfr1/10.tif]

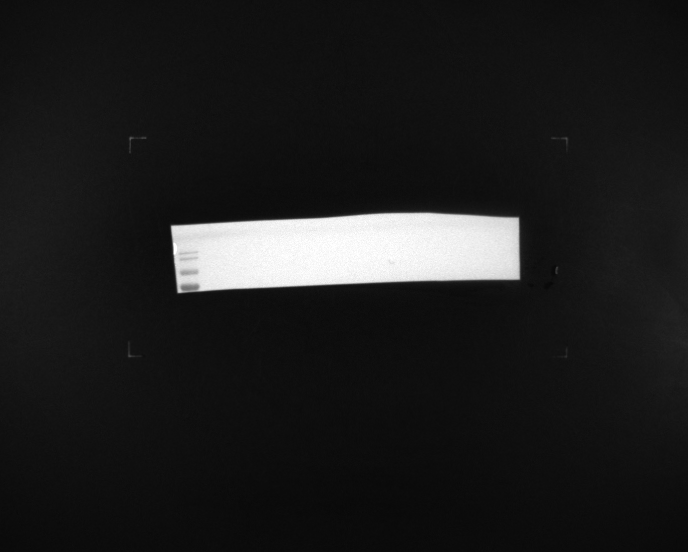

Supplement: Figure 3—source data 1. [file elife-80494-fig3-data1.zip › Fig3/Fig3E/Tfr1/MK.tif]

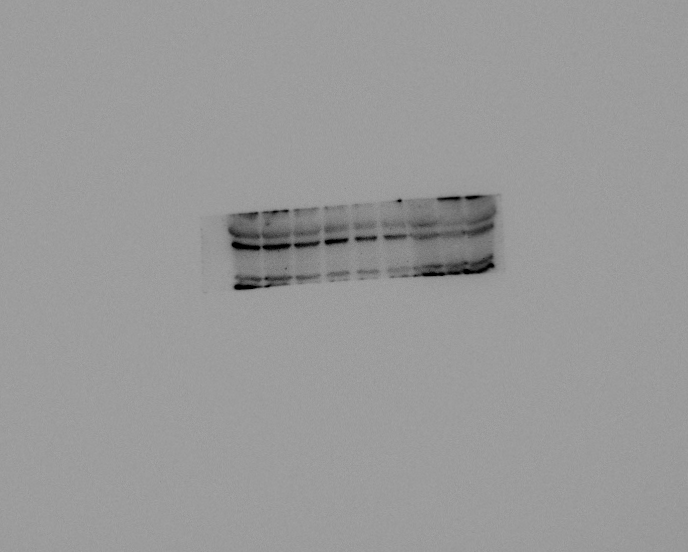

Supplement: Figure 3—source data 1. [file elife-80494-fig3-data1.zip › Fig3/Fig3E/VEGF/10.tif]

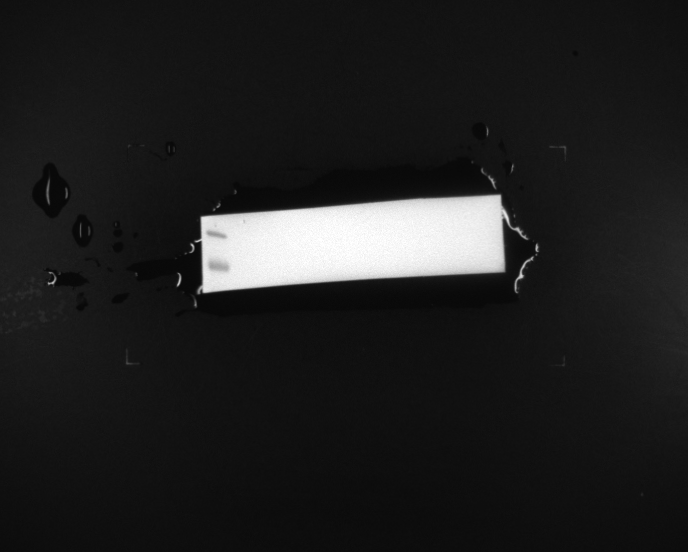

Supplement: Figure 3—source data 1. [file elife-80494-fig3-data1.zip › Fig3/Fig3E/VEGF/MK.tif]

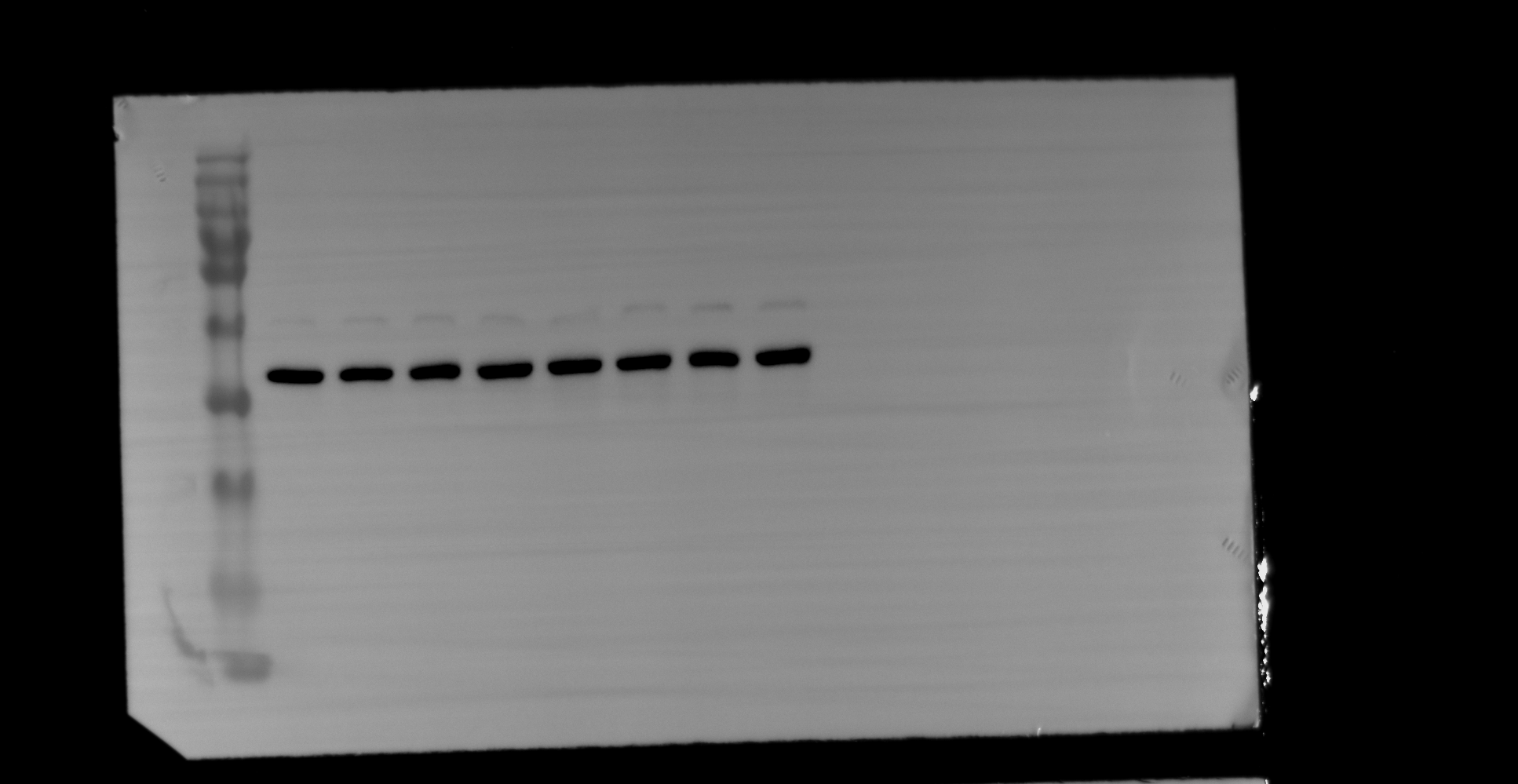

Supplement: Figure 3—figure supplement 1—source data 1. [file elife-80494-fig3-figsupp1-data1.zip › FigS2/FigS2A/ACTIN.tif]

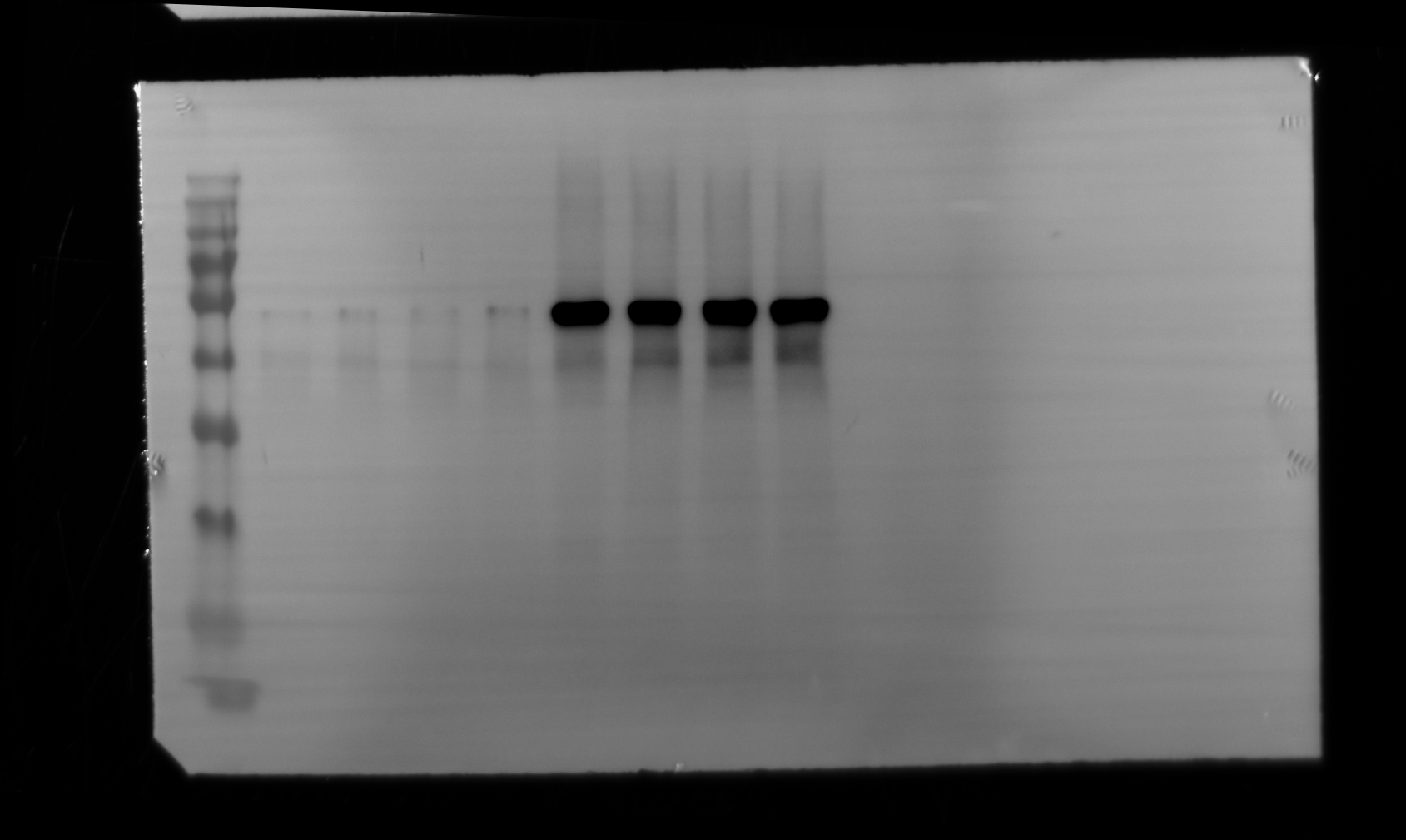

Supplement: Figure 3—figure supplement 1—source data 1. [file elife-80494-fig3-figsupp1-data1.zip › FigS2/FigS2A/FPN1.tif]

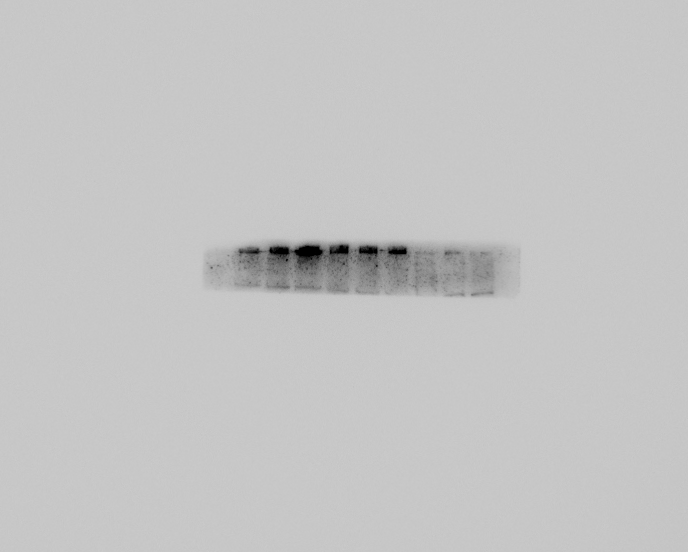

Supplement: Figure 3—figure supplement 1—source data 1. [file elife-80494-fig3-figsupp1-data1.zip › FigS2/FigS2D/ABCA1/30.tif]

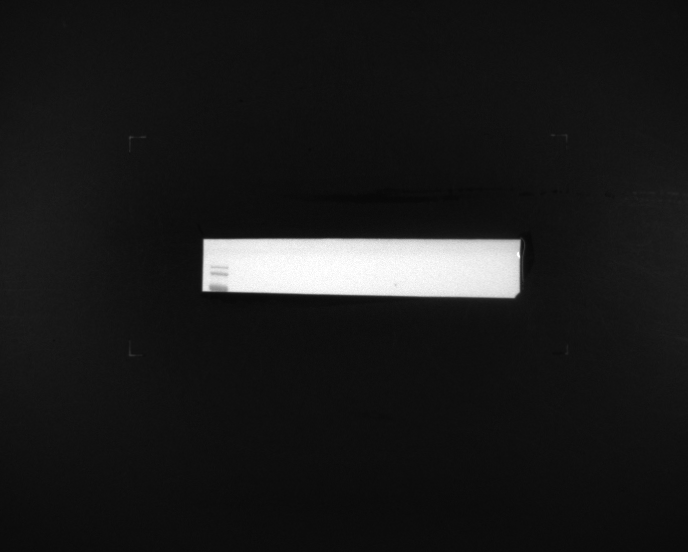

Supplement: Figure 3—figure supplement 1—source data 1. [file elife-80494-fig3-figsupp1-data1.zip › FigS2/FigS2D/ABCA1/mk.tif]

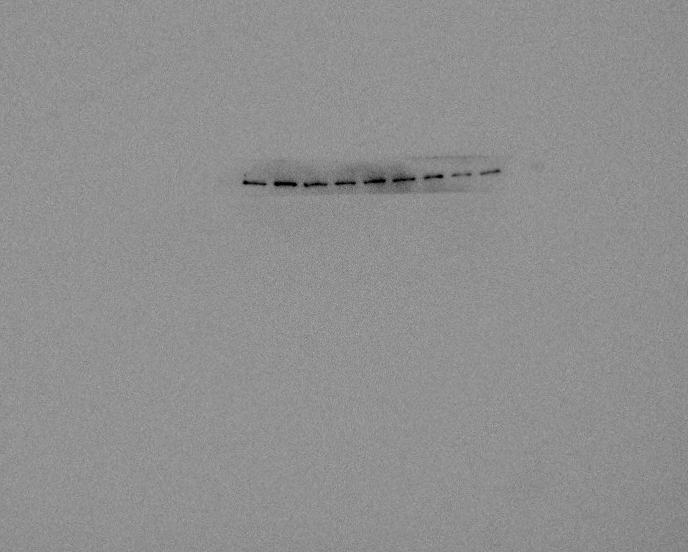

Supplement: Figure 3—figure supplement 1—source data 1. [file elife-80494-fig3-figsupp1-data1.zip › FigS2/FigS2D/ERa┴/10'.tif]

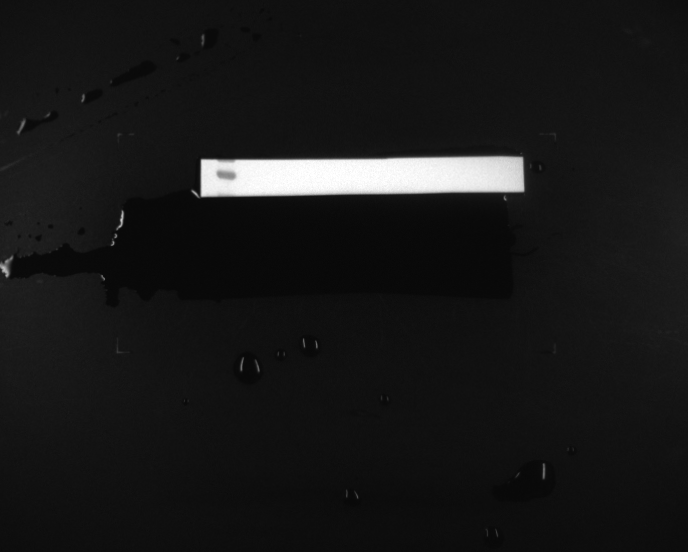

Supplement: Figure 3—figure supplement 1—source data 1. [file elife-80494-fig3-figsupp1-data1.zip › FigS2/FigS2D/ERa┴/mk.tif]

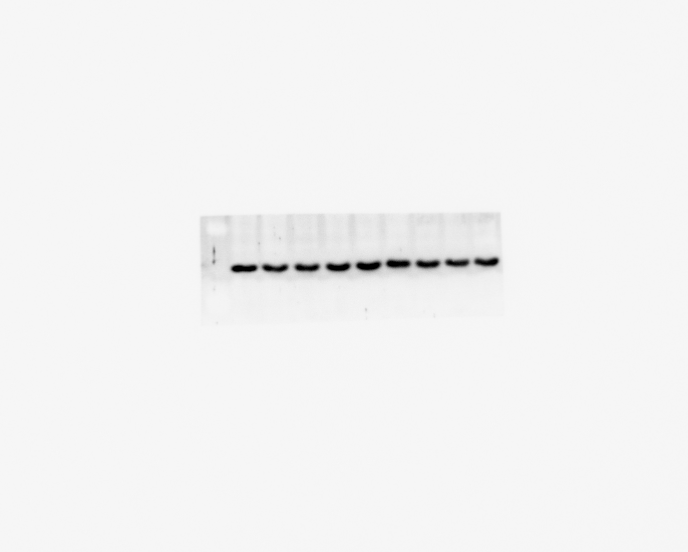

Supplement: Figure 3—figure supplement 1—source data 1. [file elife-80494-fig3-figsupp1-data1.zip › FigS2/FigS2D/Ftl/1.tif]

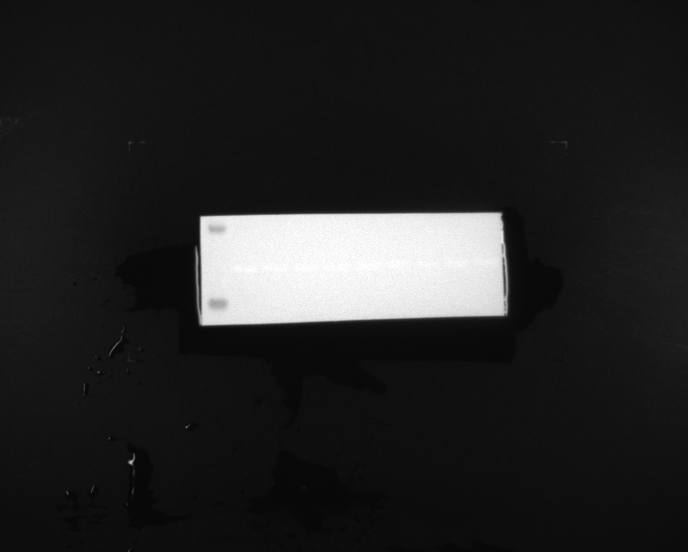

Supplement: Figure 3—figure supplement 1—source data 1. [file elife-80494-fig3-figsupp1-data1.zip › FigS2/FigS2D/Ftl/MK.tif]

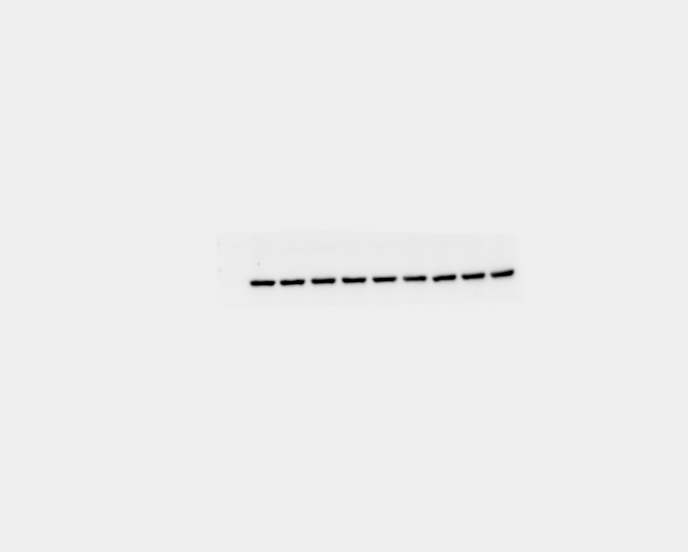

Supplement: Figure 3—figure supplement 1—source data 1. [file elife-80494-fig3-figsupp1-data1.zip › FigS2/FigS2D/GD/10.tif]

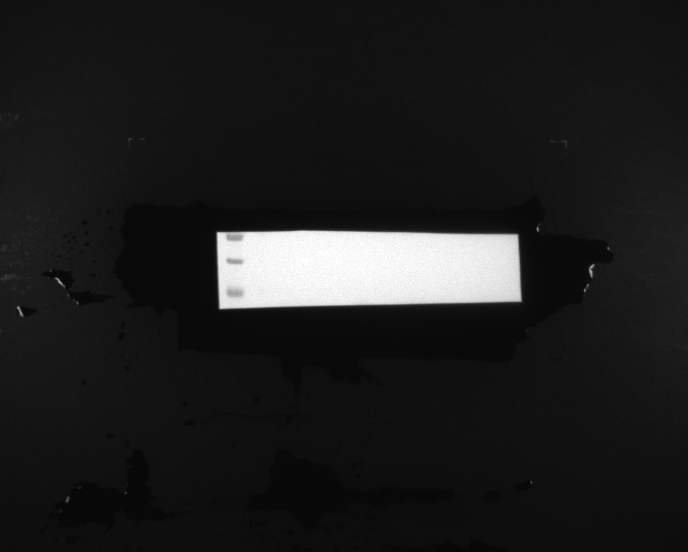

Supplement: Figure 3—figure supplement 1—source data 1. [file elife-80494-fig3-figsupp1-data1.zip › FigS2/FigS2D/GD/MK.tif]

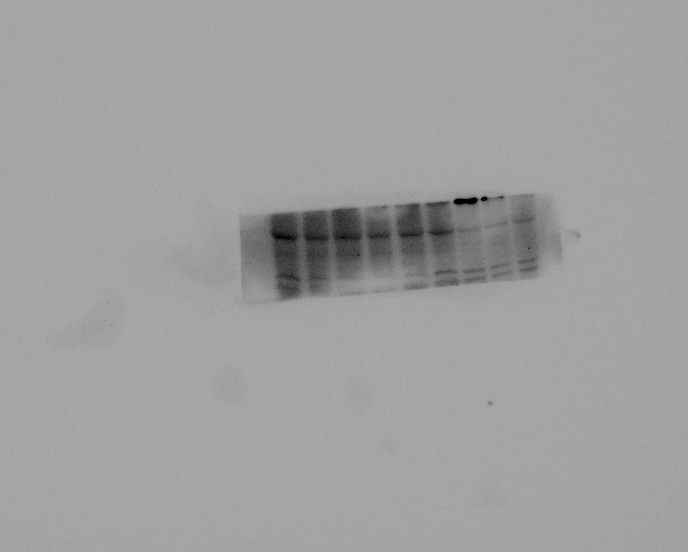

Supplement: Figure 3—figure supplement 1—source data 1. [file elife-80494-fig3-figsupp1-data1.zip › FigS2/FigS2D/VEGF/60'.tif]

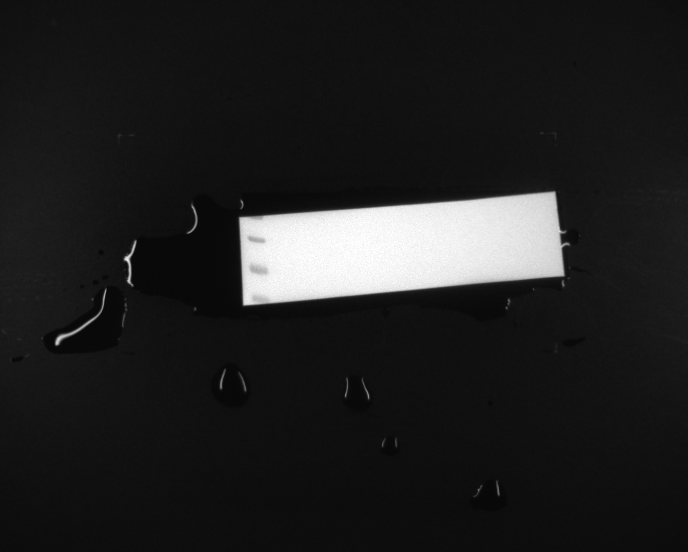

Supplement: Figure 3—figure supplement 1—source data 1. [file elife-80494-fig3-figsupp1-data1.zip › FigS2/FigS2D/VEGF/mk.tif]

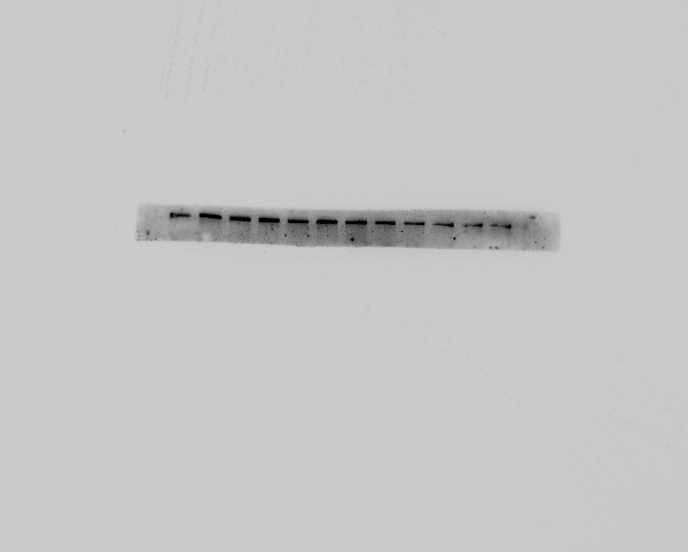

Supplement: Figure 4—source data 1. [file elife-80494-fig4-data1.zip › Figure 4 - Source data 1/Fig4A/ABCA1/60.tif]

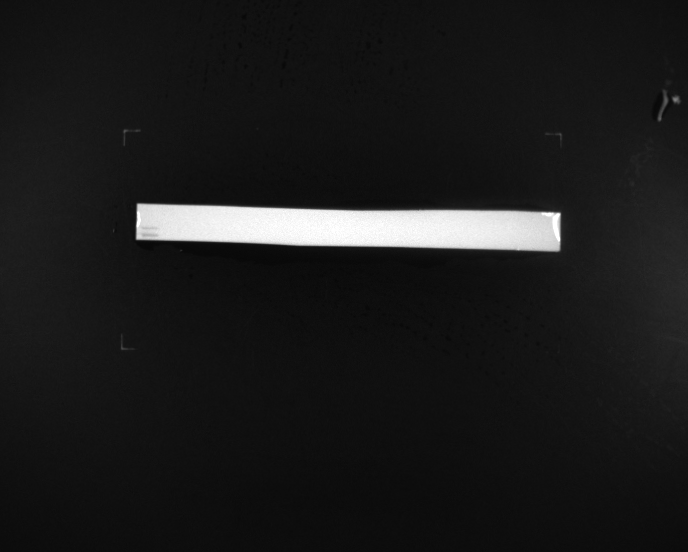

Supplement: Figure 4—source data 1. [file elife-80494-fig4-data1.zip › Figure 4 - Source data 1/Fig4A/ABCA1/MK.tif]

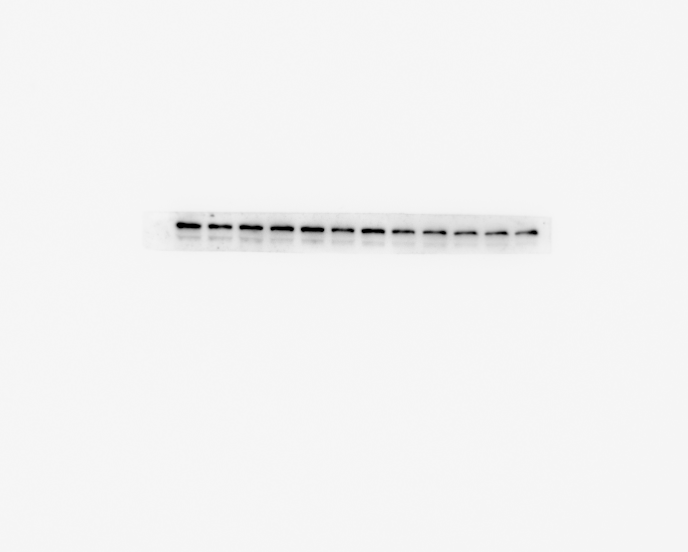

Supplement: Figure 4—source data 1. [file elife-80494-fig4-data1.zip › Figure 4 - Source data 1/Fig4A/ERa┴/10'.tif]

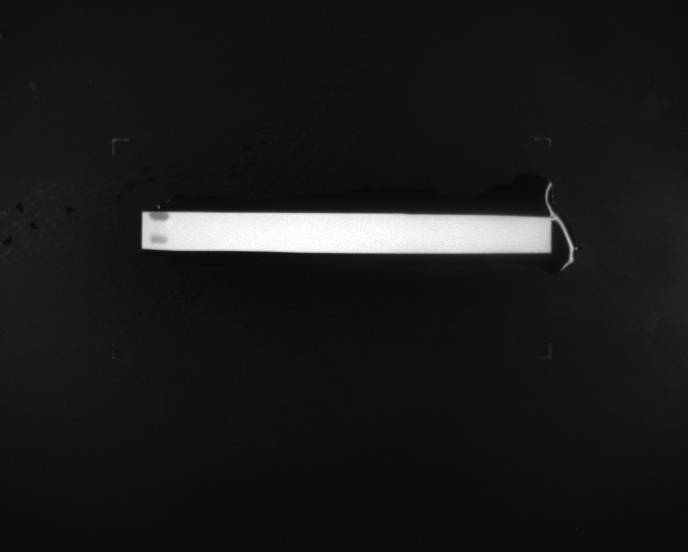

Supplement: Figure 4—source data 1. [file elife-80494-fig4-data1.zip › Figure 4 - Source data 1/Fig4A/ERa┴/MK'.tif]

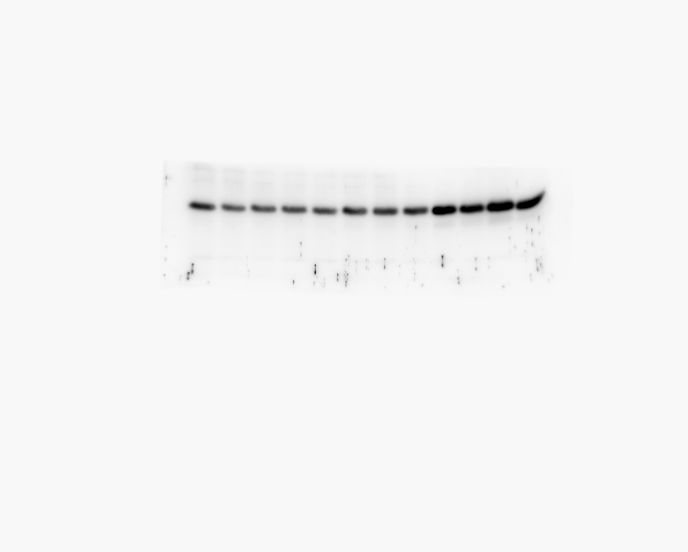

Supplement: Figure 4—source data 1. [file elife-80494-fig4-data1.zip › Figure 4 - Source data 1/Fig4A/Ftl/10.tif]

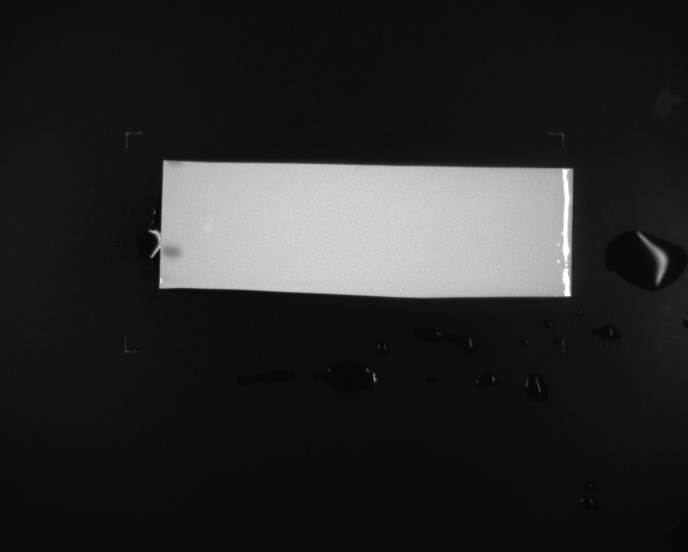

Supplement: Figure 4—source data 1. [file elife-80494-fig4-data1.zip › Figure 4 - Source data 1/Fig4A/Ftl/MK.tif]

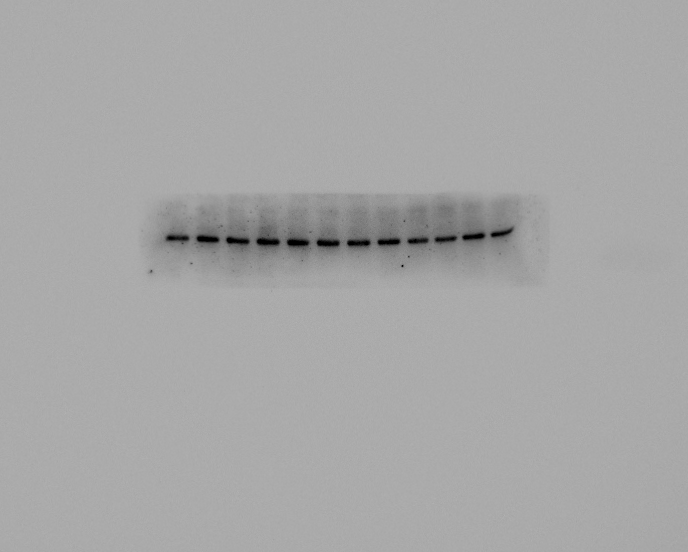

Supplement: Figure 4—source data 1. [file elife-80494-fig4-data1.zip › Figure 4 - Source data 1/Fig4A/GAPDH/60.tif]

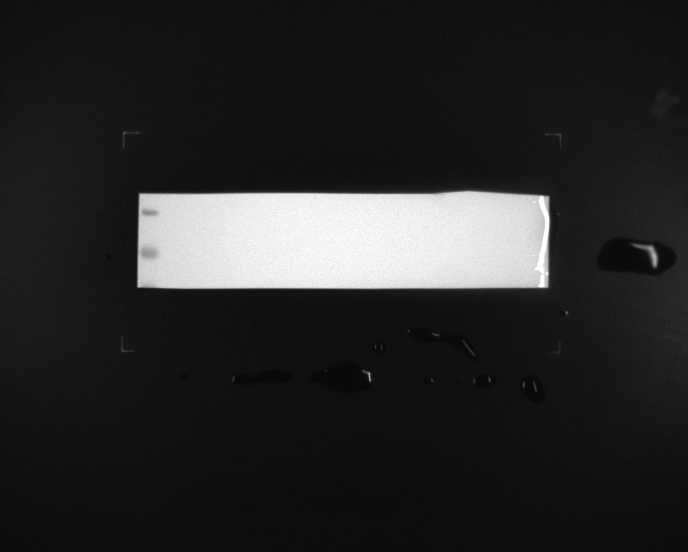

Supplement: Figure 4—source data 1. [file elife-80494-fig4-data1.zip › Figure 4 - Source data 1/Fig4A/GAPDH/MK.tif]

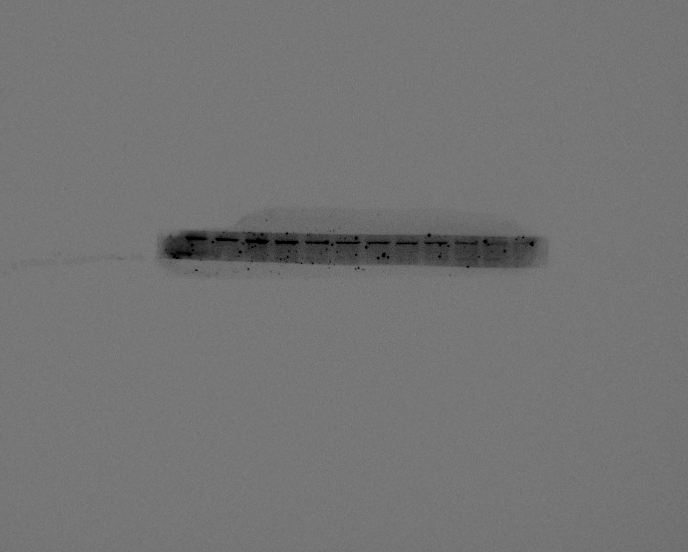

Supplement: Figure 4—source data 1. [file elife-80494-fig4-data1.zip › Figure 4 - Source data 1/Fig4B/ERa┴/30.tif]

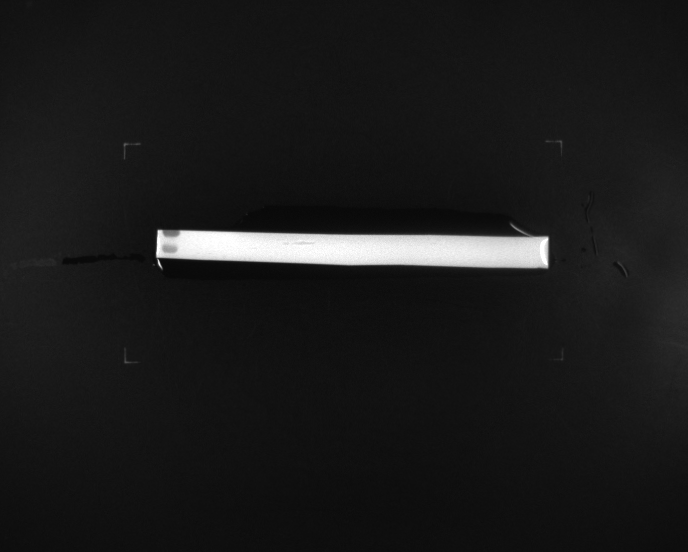

Supplement: Figure 4—source data 1. [file elife-80494-fig4-data1.zip › Figure 4 - Source data 1/Fig4B/ERa┴/MK.tif]

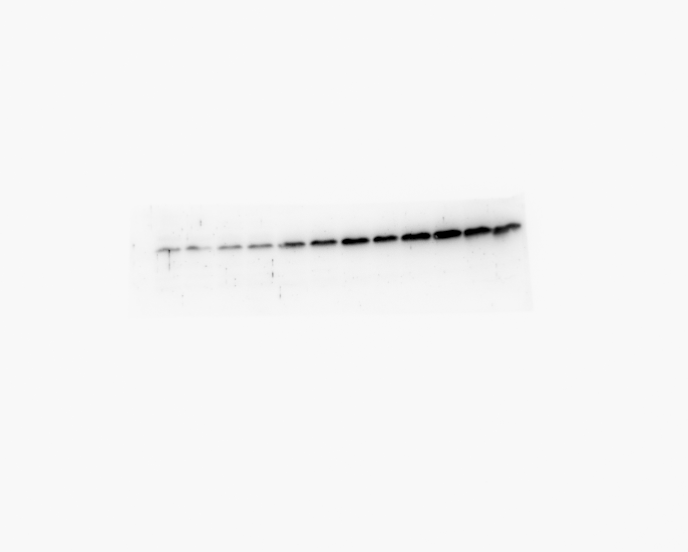

Supplement: Figure 4—source data 1. [file elife-80494-fig4-data1.zip › Figure 4 - Source data 1/Fig4B/Ftl/3.tif]

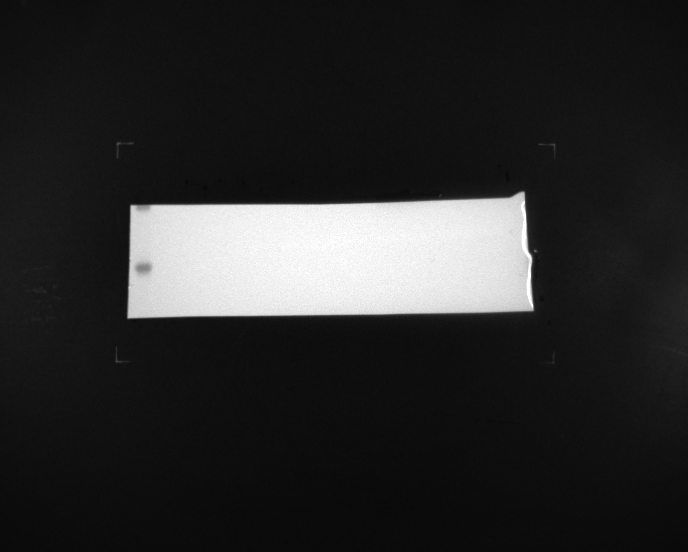

Supplement: Figure 4—source data 1. [file elife-80494-fig4-data1.zip › Figure 4 - Source data 1/Fig4B/Ftl/MK.tif]

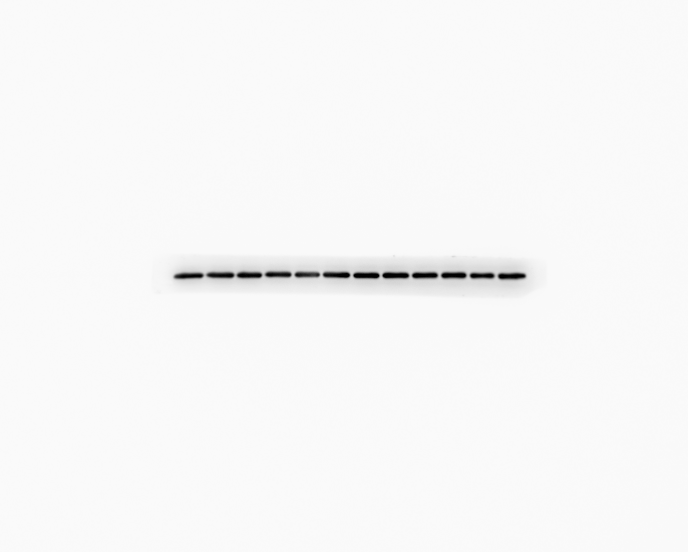

Supplement: Figure 4—source data 1. [file elife-80494-fig4-data1.zip › Figure 4 - Source data 1/Fig4B/GAPDH/10.tif]

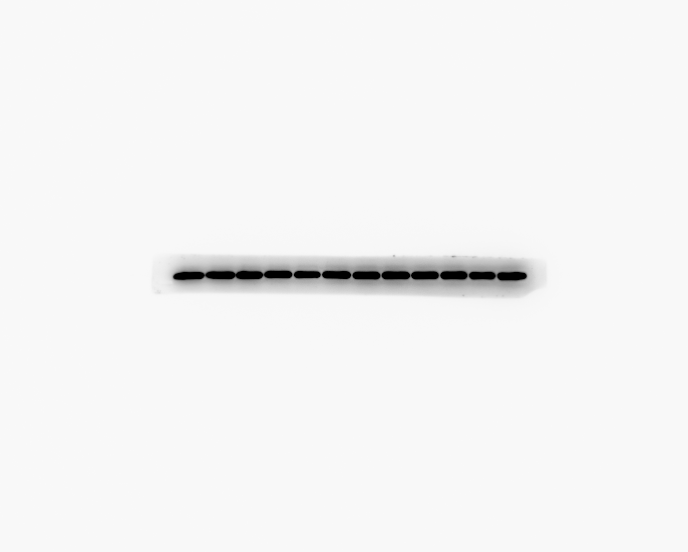

Supplement: Figure 4—source data 1. [file elife-80494-fig4-data1.zip › Figure 4 - Source data 1/Fig4B/GAPDH/30.tif]

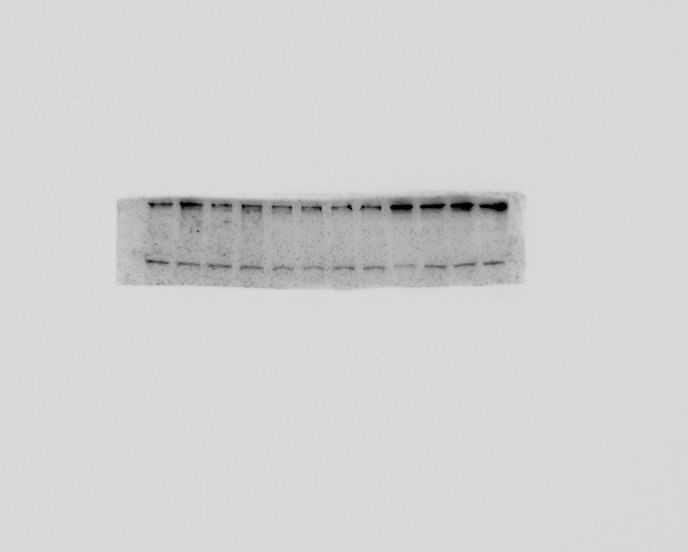

Supplement: Figure 4—source data 1. [file elife-80494-fig4-data1.zip › Figure 4 - Source data 1/Fig4C/ABCA1/30.tif]

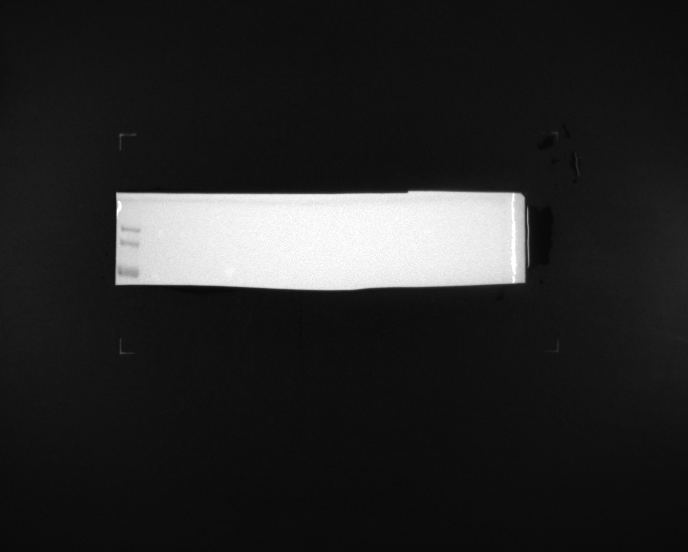

Supplement: Figure 4—source data 1. [file elife-80494-fig4-data1.zip › Figure 4 - Source data 1/Fig4C/ABCA1/mk.tif]

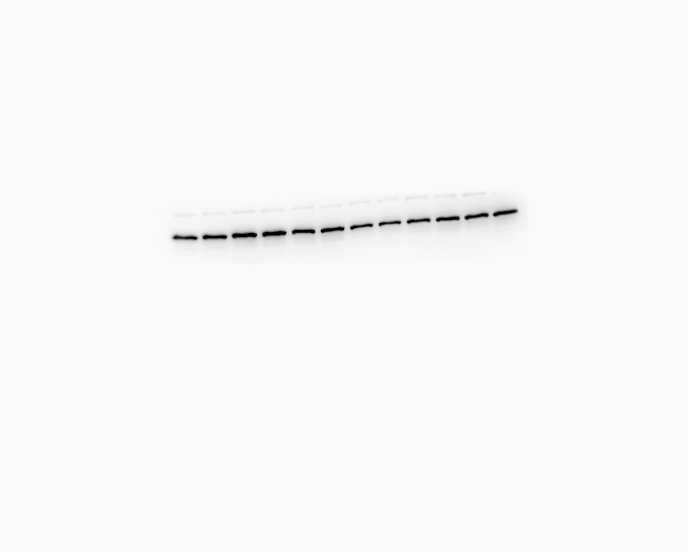

Supplement: Figure 4—source data 1. [file elife-80494-fig4-data1.zip › Figure 4 - Source data 1/Fig4C/ERa┴/10.tif]

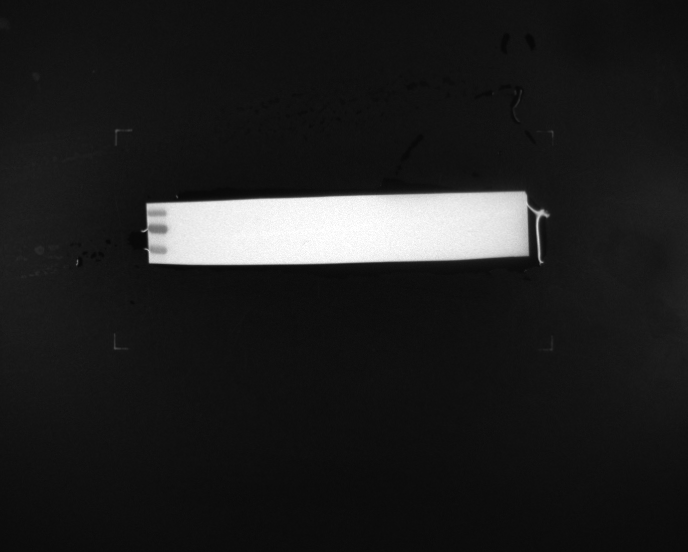

Supplement: Figure 4—source data 1. [file elife-80494-fig4-data1.zip › Figure 4 - Source data 1/Fig4C/ERa┴/MK.tif]

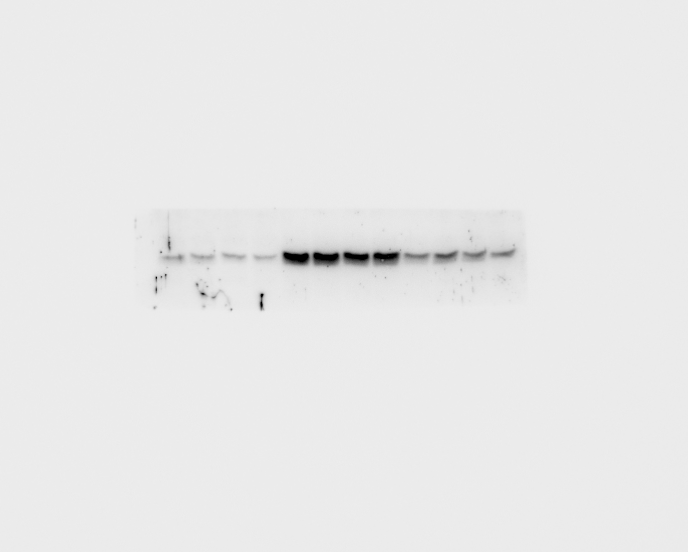

Supplement: Figure 4—source data 1. [file elife-80494-fig4-data1.zip › Figure 4 - Source data 1/Fig4C/Ftl/10.tif]

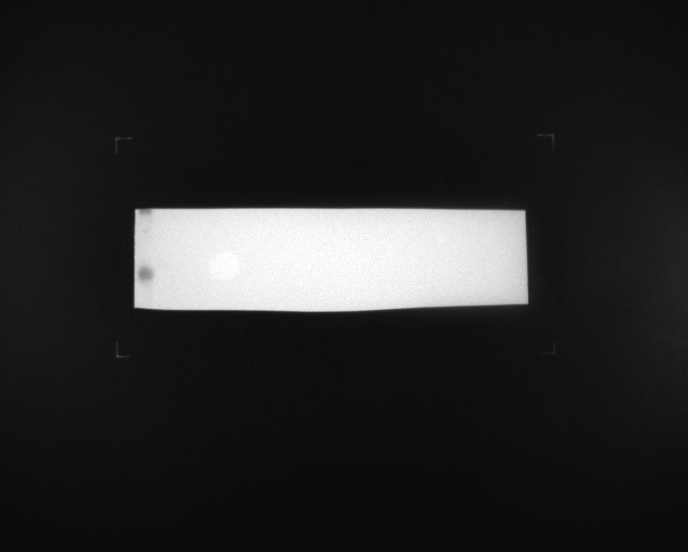

Supplement: Figure 4—source data 1. [file elife-80494-fig4-data1.zip › Figure 4 - Source data 1/Fig4C/Ftl/mk.tif]

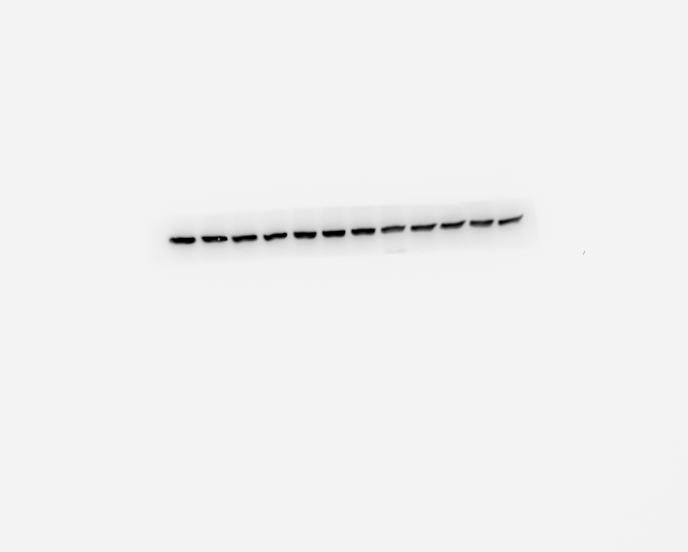

Supplement: Figure 4—source data 1. [file elife-80494-fig4-data1.zip › Figure 4 - Source data 1/Fig4C/GAPDH/60.tif]

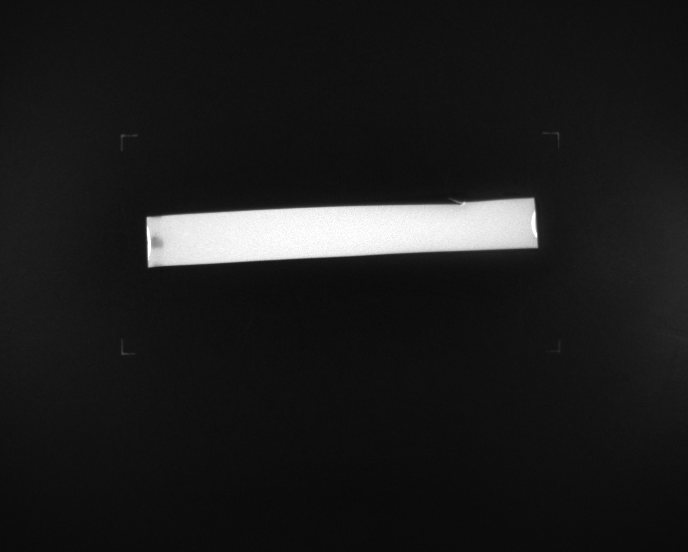

Supplement: Figure 4—source data 1. [file elife-80494-fig4-data1.zip › Figure 4 - Source data 1/Fig4C/GAPDH/mk.tif]

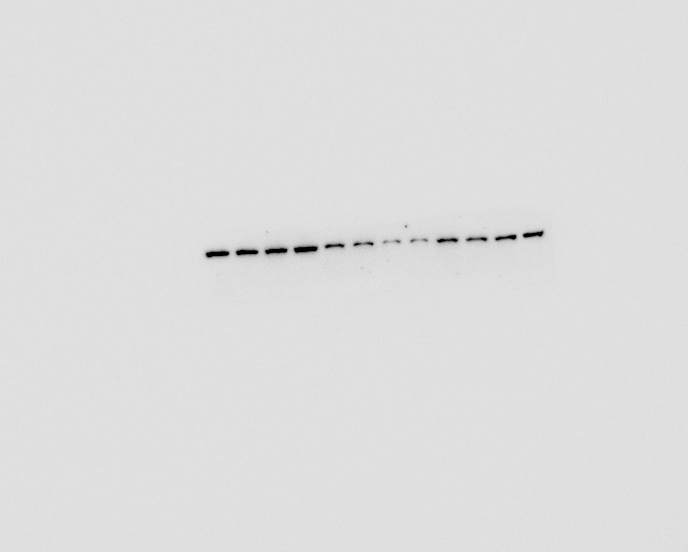

Supplement: Figure 4—source data 1. [file elife-80494-fig4-data1.zip › Figure 4 - Source data 1/Fig4E/ERa┴/1.tif]

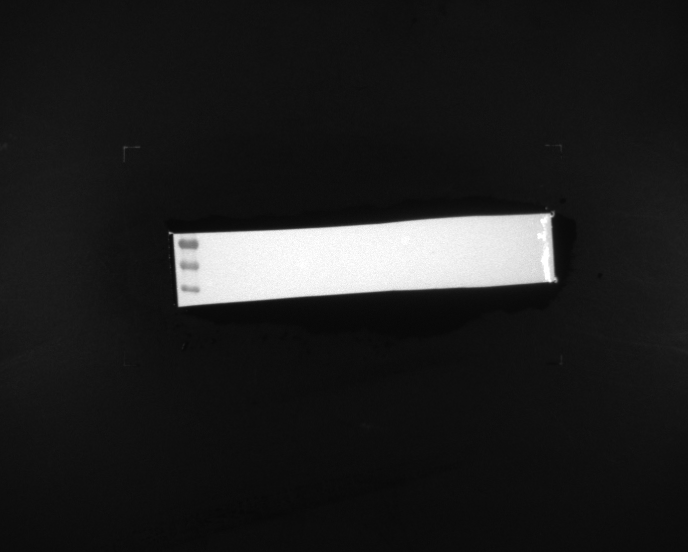

Supplement: Figure 4—source data 1. [file elife-80494-fig4-data1.zip › Figure 4 - Source data 1/Fig4E/ERa┴/MK.tif]

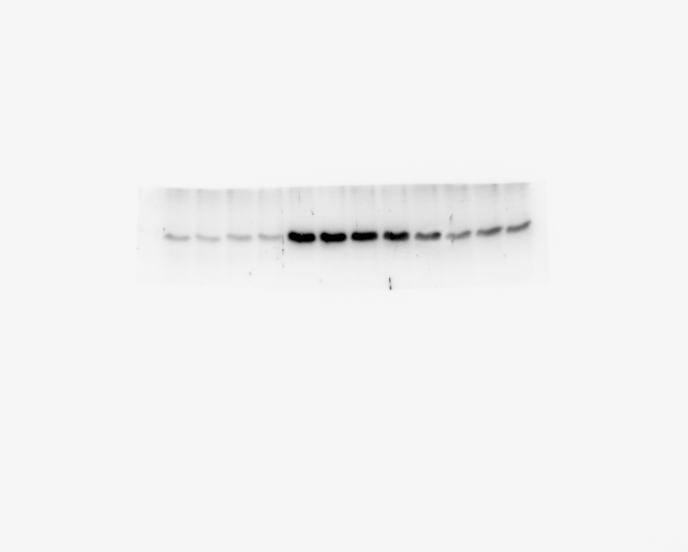

Supplement: Figure 4—source data 1. [file elife-80494-fig4-data1.zip › Figure 4 - Source data 1/Fig4E/Ftl/3.tif]

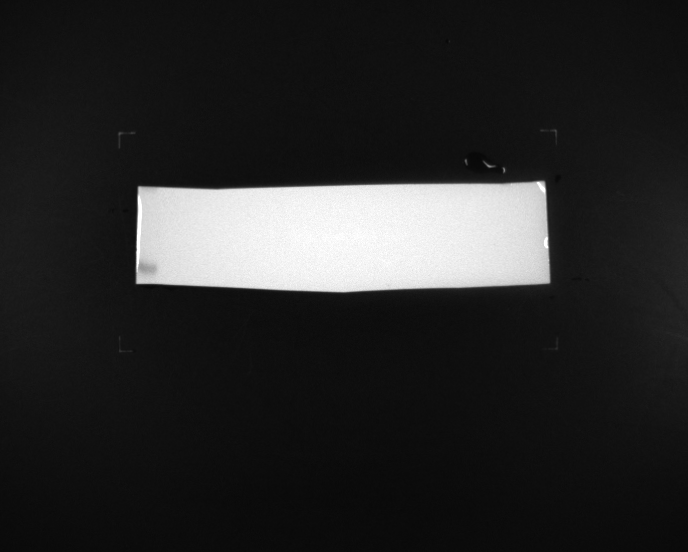

Supplement: Figure 4—source data 1. [file elife-80494-fig4-data1.zip › Figure 4 - Source data 1/Fig4E/Ftl/MK.tif]

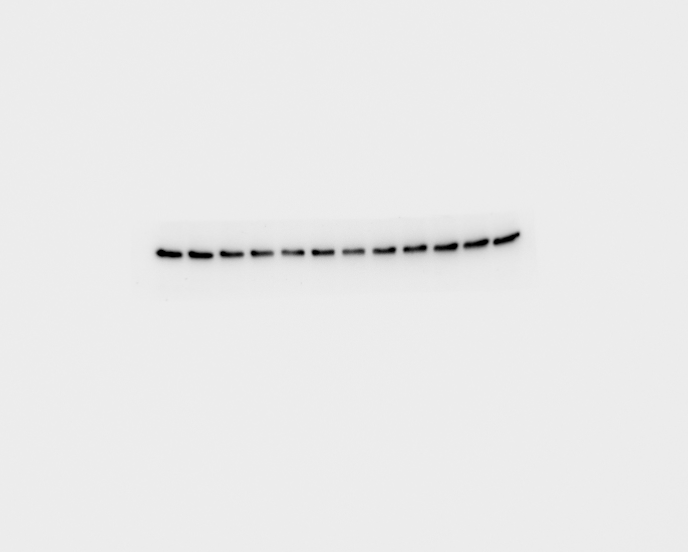

Supplement: Figure 4—source data 1. [file elife-80494-fig4-data1.zip › Figure 4 - Source data 1/Fig4E/GAPDH/10.tif]

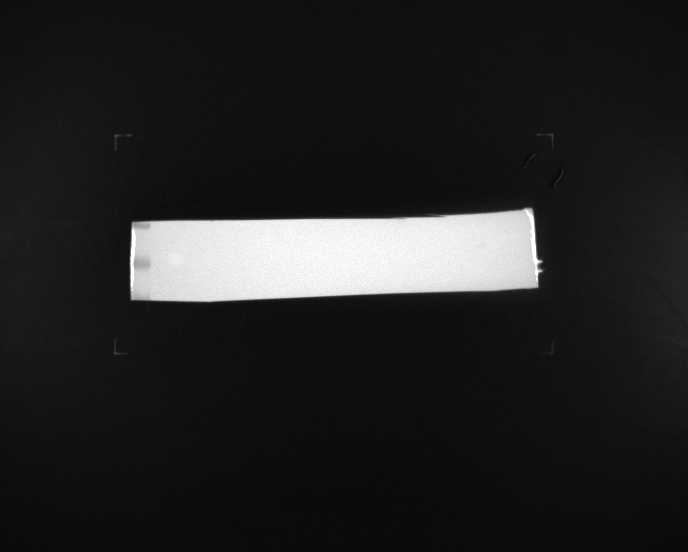

Supplement: Figure 4—source data 1. [file elife-80494-fig4-data1.zip › Figure 4 - Source data 1/Fig4E/GAPDH/MK.tif]

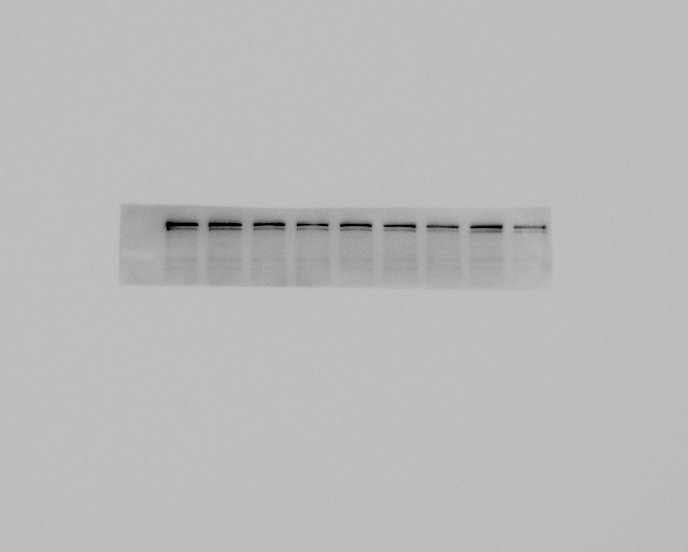

Supplement: Figure 4—source data 1. [file elife-80494-fig4-data1.zip › Figure 4 - Source data 1/Fig4F/ERa┴/30.tif]

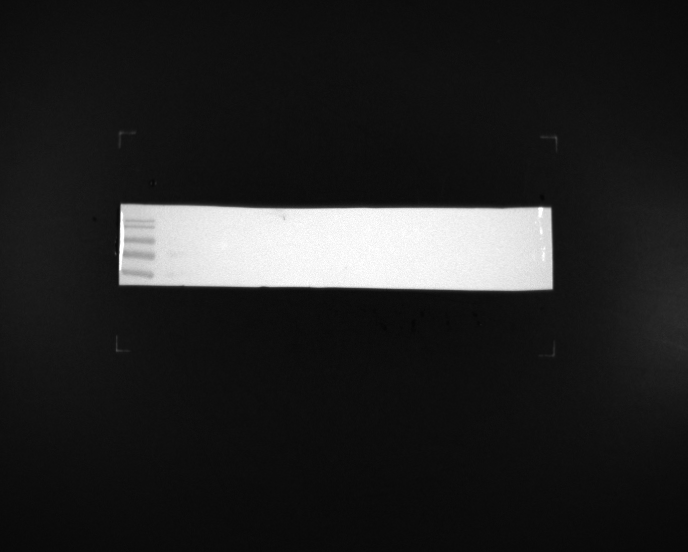

Supplement: Figure 4—source data 1. [file elife-80494-fig4-data1.zip › Figure 4 - Source data 1/Fig4F/ERa┴/mk.tif]

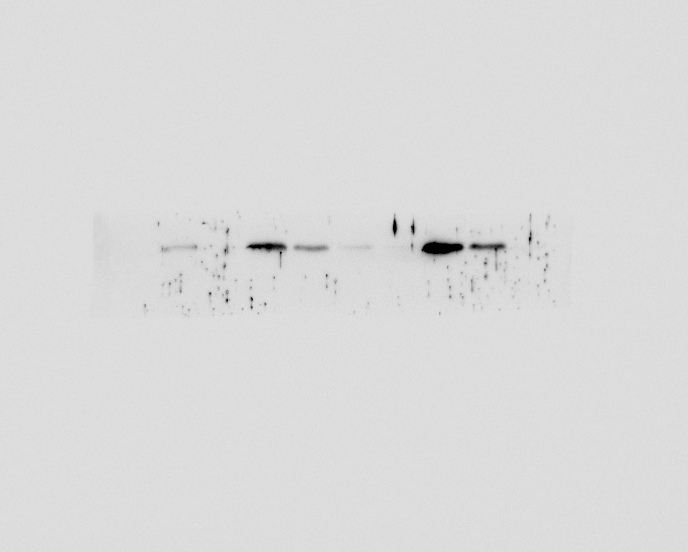

Supplement: Figure 4—source data 1. [file elife-80494-fig4-data1.zip › Figure 4 - Source data 1/Fig4F/Ftl/1.tif]

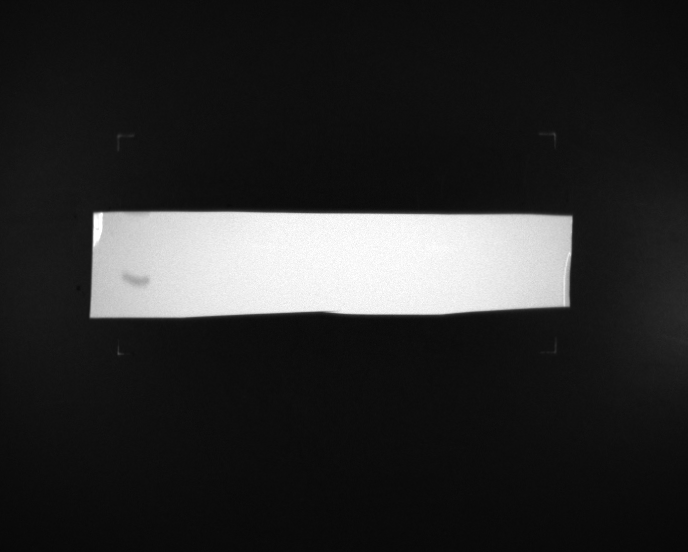

Supplement: Figure 4—source data 1. [file elife-80494-fig4-data1.zip › Figure 4 - Source data 1/Fig4F/Ftl/mk.tif]

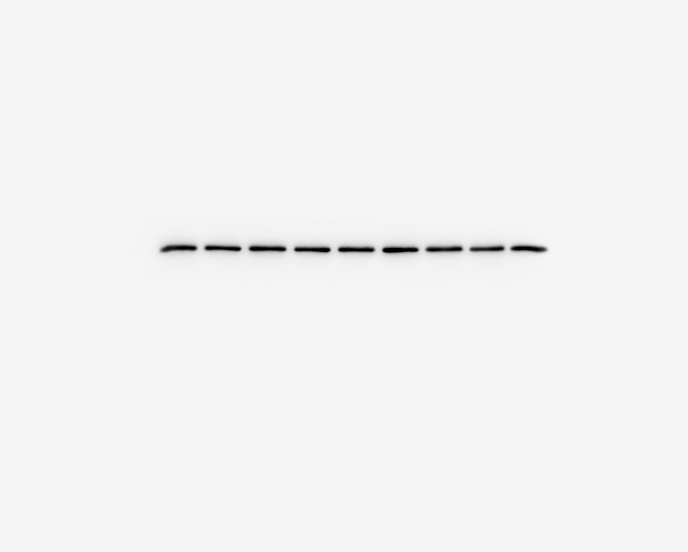

Supplement: Figure 4—source data 1. [file elife-80494-fig4-data1.zip › Figure 4 - Source data 1/Fig4F/GAPDH/1.tif]

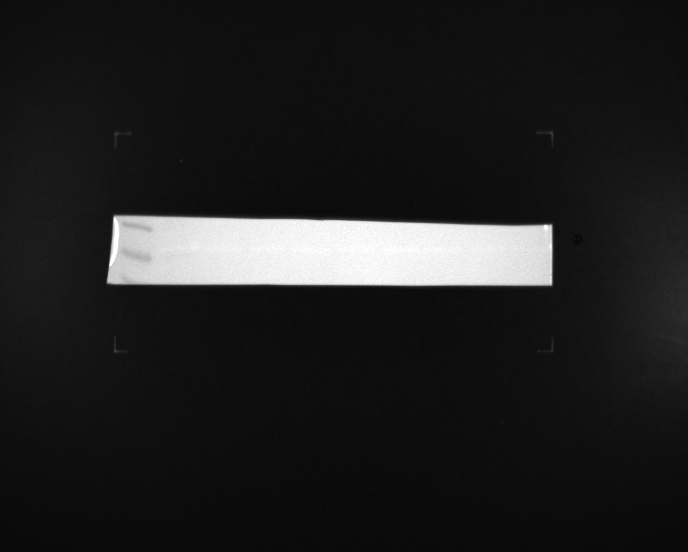

Supplement: Figure 4—source data 1. [file elife-80494-fig4-data1.zip › Figure 4 - Source data 1/Fig4F/GAPDH/mk.tif]

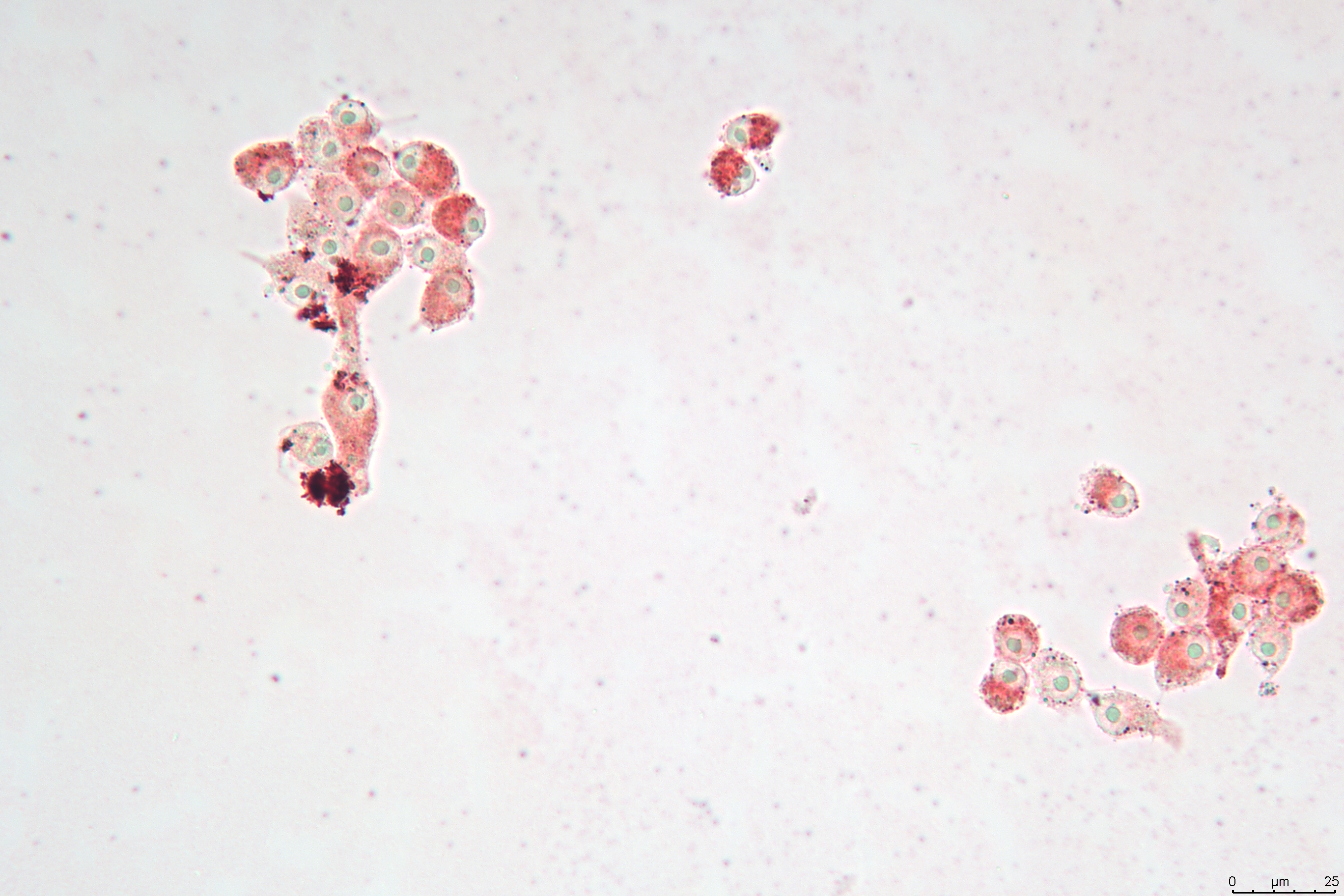

Supplement: Figure 4—source data 2. [file elife-80494-fig4-data2.zip › Figure 4G -2/Project_ef-40-2.tif]

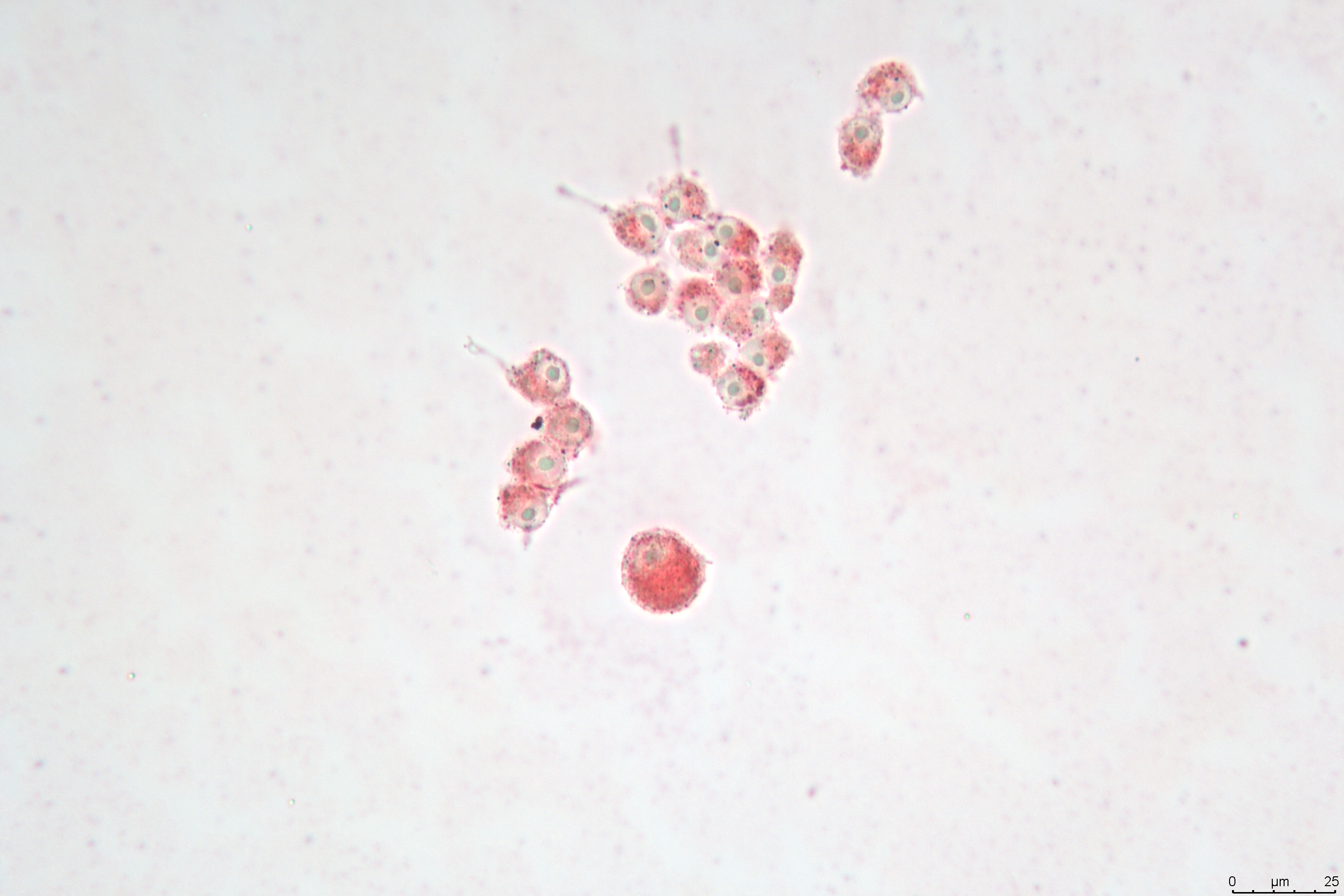

Supplement: Figure 4—source data 2. [file elife-80494-fig4-data2.zip › Figure 4G -2/Project_ef-40-3.tif]

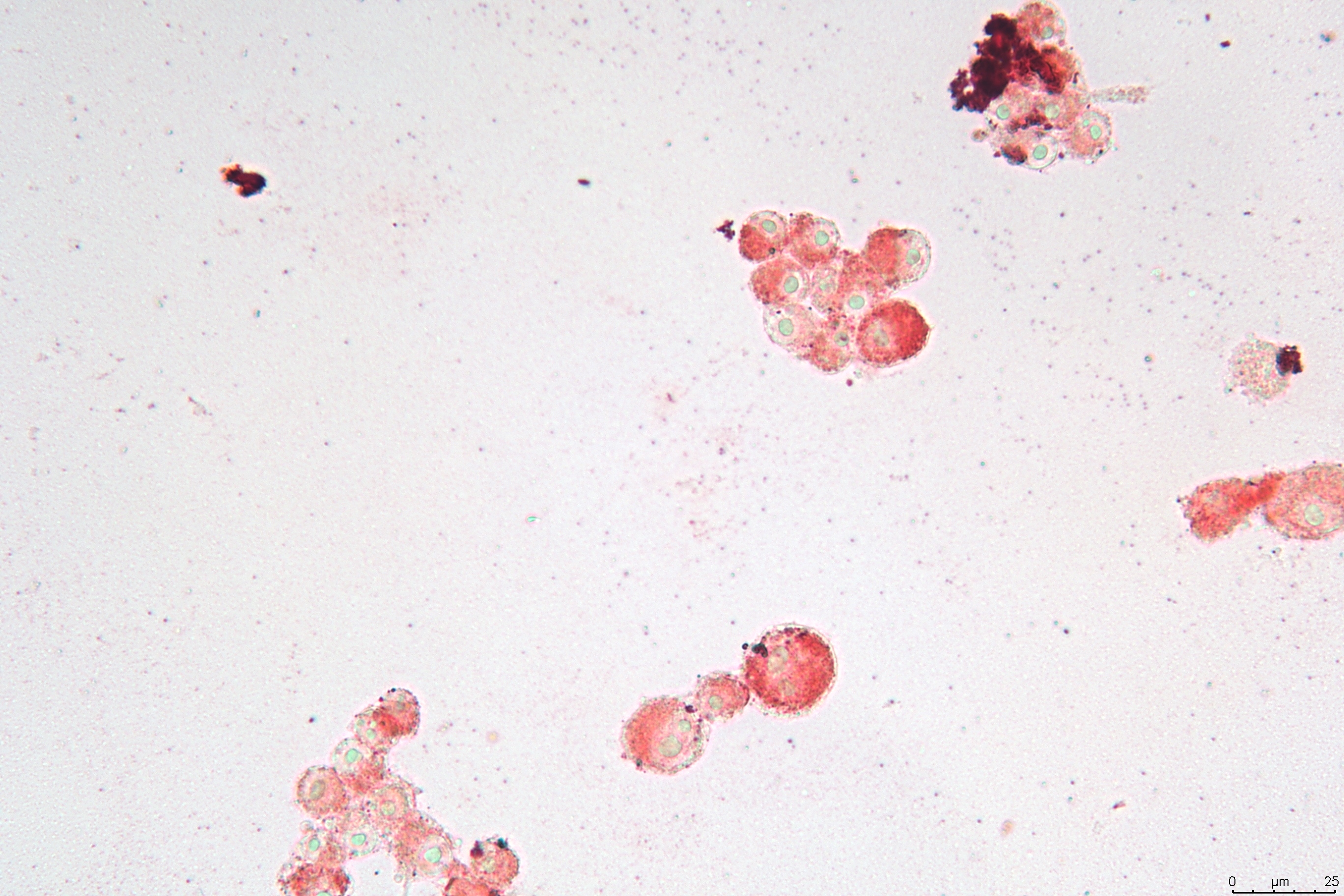

Supplement: Figure 4—source data 2. [file elife-80494-fig4-data2.zip › Figure 4G -2/Project_ef-40-4.tif]

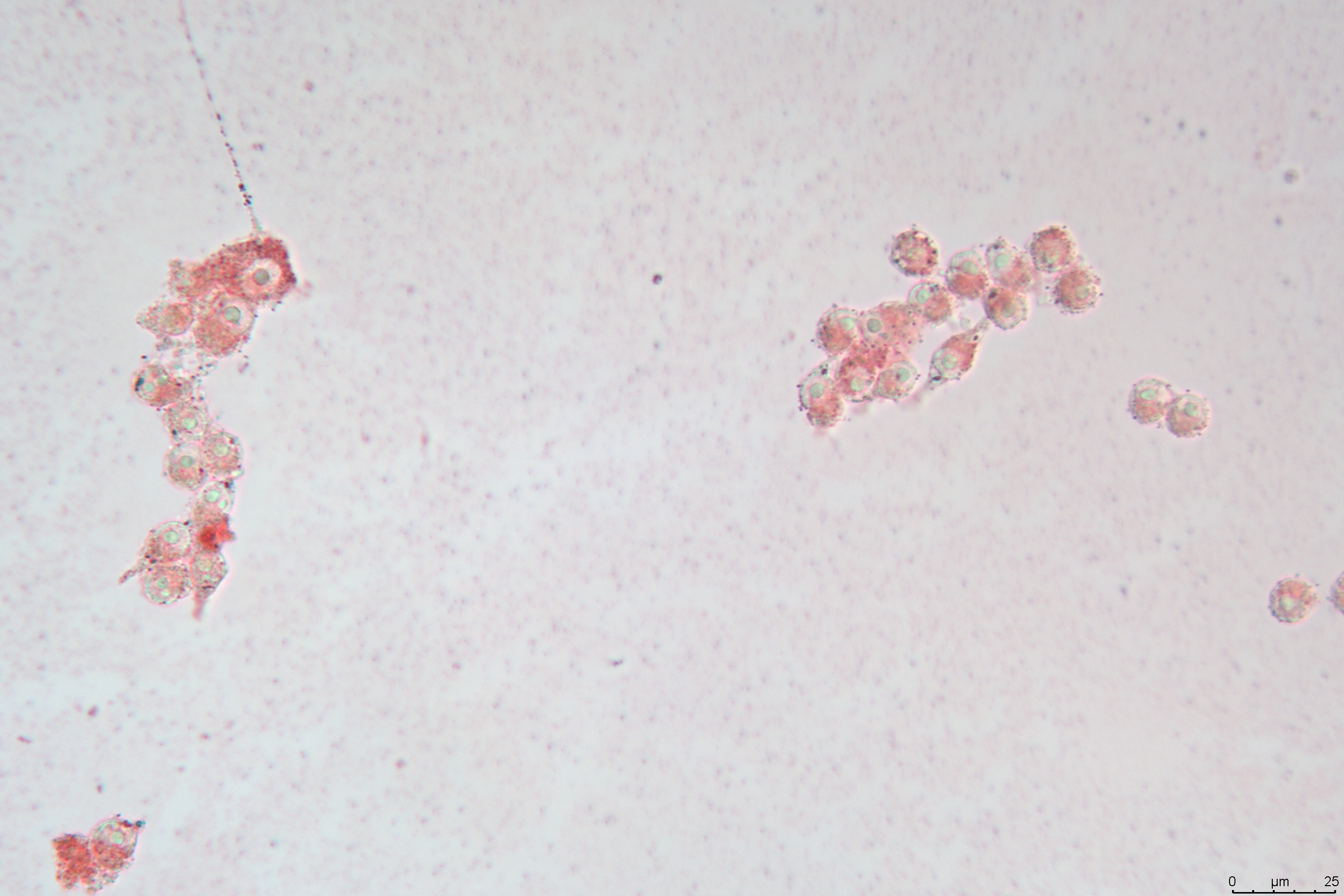

Supplement: Figure 4—source data 2. [file elife-80494-fig4-data2.zip › Figure 4G -2/Project_f-40-1.tif]

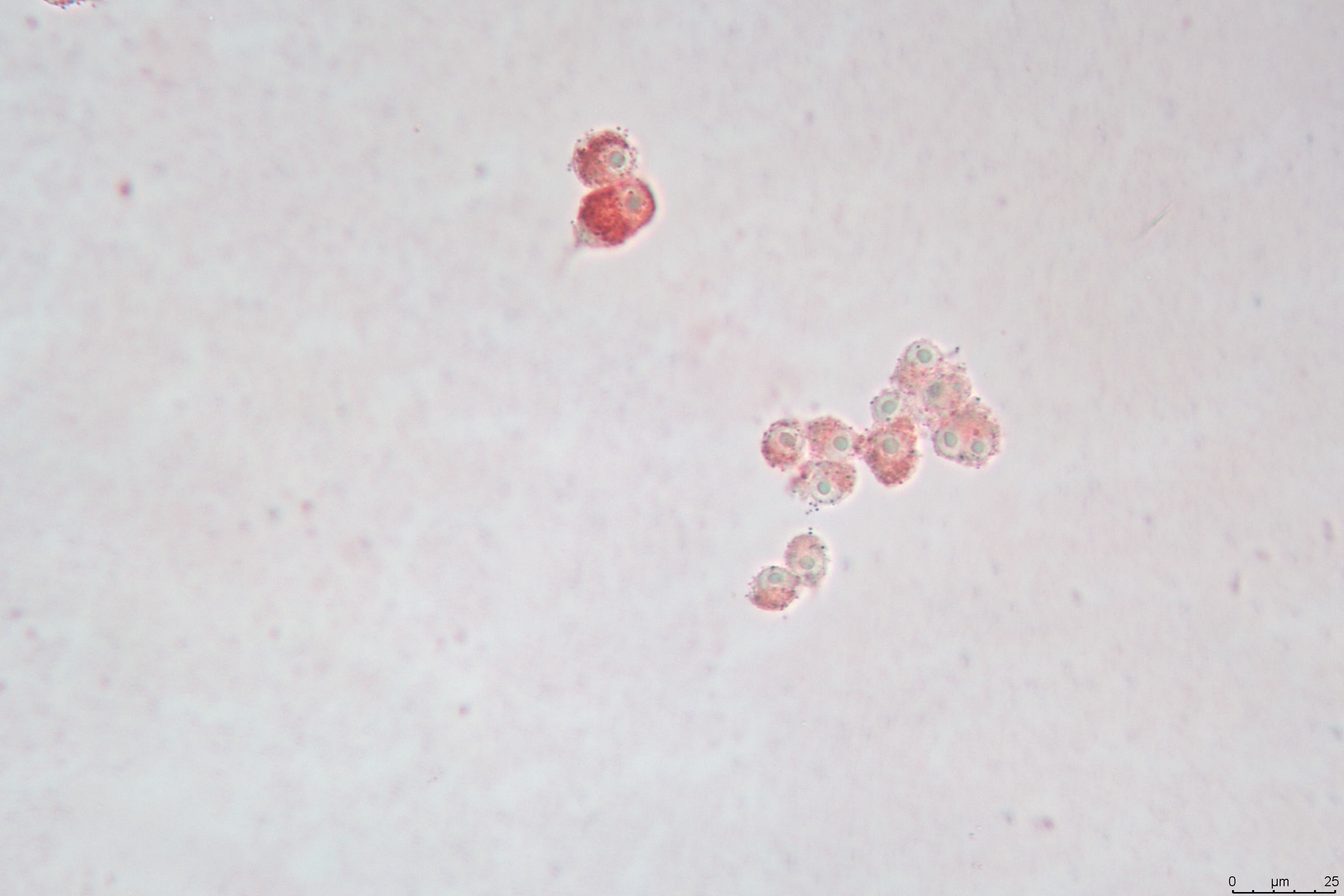

Supplement: Figure 4—source data 2. [file elife-80494-fig4-data2.zip › Figure 4G -2/Project_f-40-2.tif]

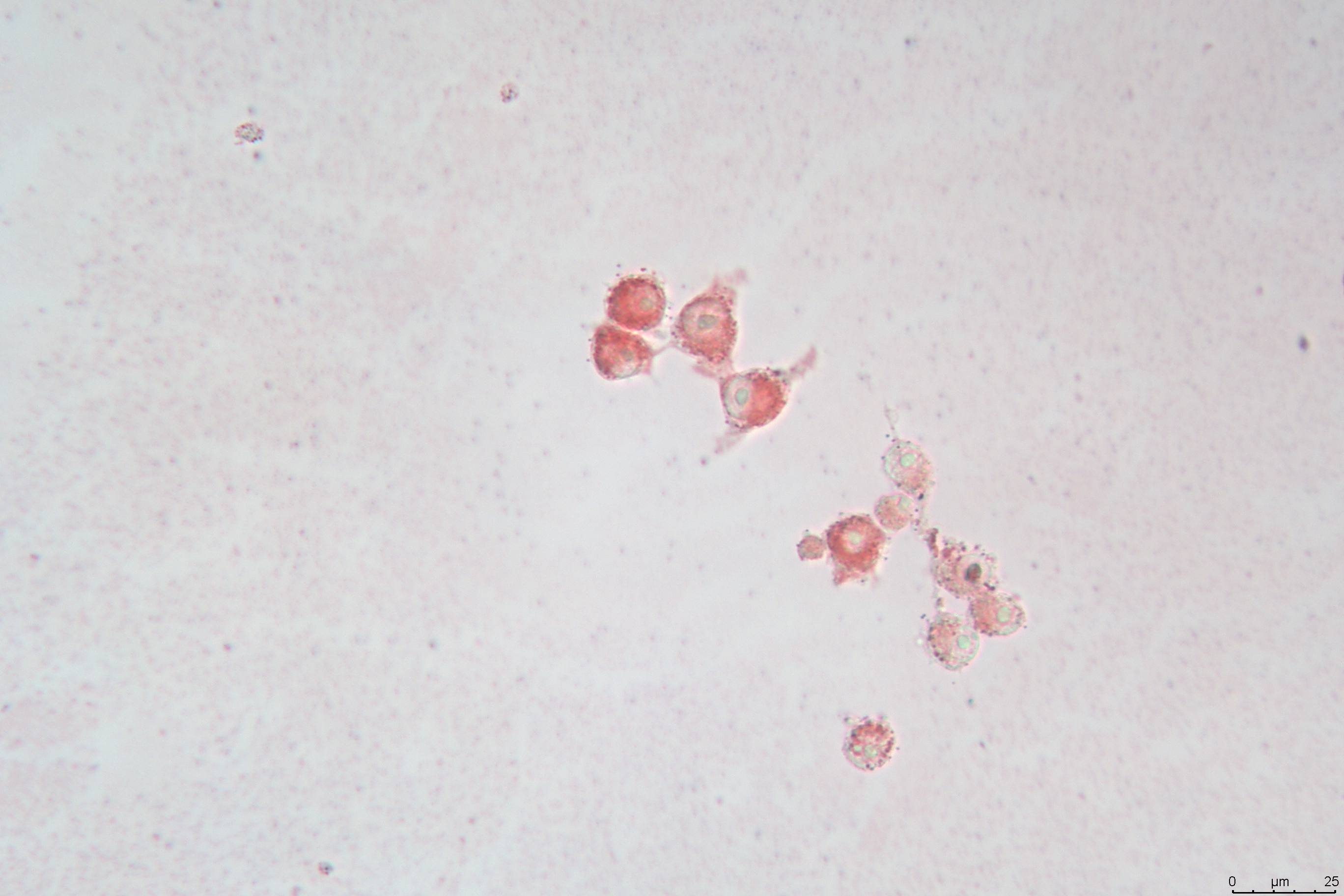

Supplement: Figure 4—source data 2. [file elife-80494-fig4-data2.zip › Figure 4G -2/Project_f-40-3.tif]

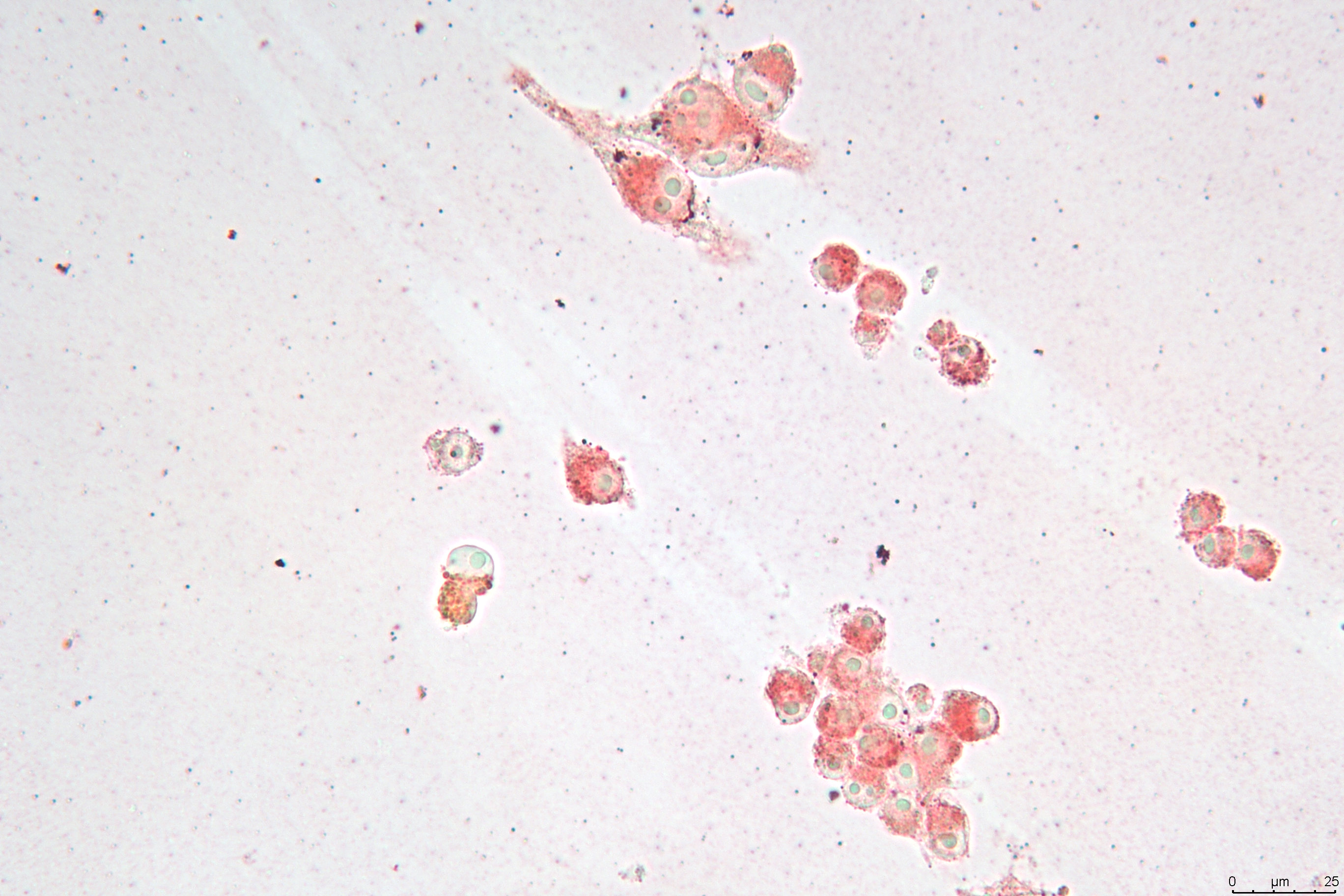

Supplement: Figure 4—source data 2. [file elife-80494-fig4-data2.zip › Figure 4G -2/Project_f-40-4.tif]
