# Supplementary material for: Hormone replacement therapy for postmenopausal atherosclerosis is offset by late age iron deposition
Source: eLife. 2023 Aug 10;12:e80494. doi: 10.7554/eLife.80494 (PMC10414966; doi:10.7554/eLife.80494)
Supplement: Table 1—source data 1. [file elife-80494-table1-data1.zip › Table S1/Patients.docx]

|  | EPM | LPM | p value |
| --- | --- | --- | --- |
| Age | 59.3±3.7 | 77.5±4.5 | ＜0.0001 |
| Risk factors |  |  |  |
| Smoking history | 2 | 1 | 0.232 |
| Hypercholesterolemia | 4 | 8 | 1.000 |
| Hypertension | 7 | 8 | 0.334 |
| Coronary artery disease | 4 | 6 | 0.241 |
| Cerebral infarction | 4 | 5 | 0.548 |
| Cholesterol lowering drug usage | 5 | 8 | 1.000 |
| Symptoms |  |  |  |
| Chest distress | 5 | 6 | 0.548 |
| Dizzy | 6 | 8 | 0.081 |
| Plaque type |  |  |  |
| Stable plaque | 5 | 3 | 0.207 |
| Vulnerable plaque | 5 | 7 | 0.207 |
