# Supplementary figures and images for: Hormone replacement therapy for postmenopausal atherosclerosis is offset by late age iron deposition (part 2 of 2)
Source: eLife. 2023 Aug 10;12:e80494. doi: 10.7554/eLife.80494 (PMC10414966; doi:10.7554/eLife.80494)

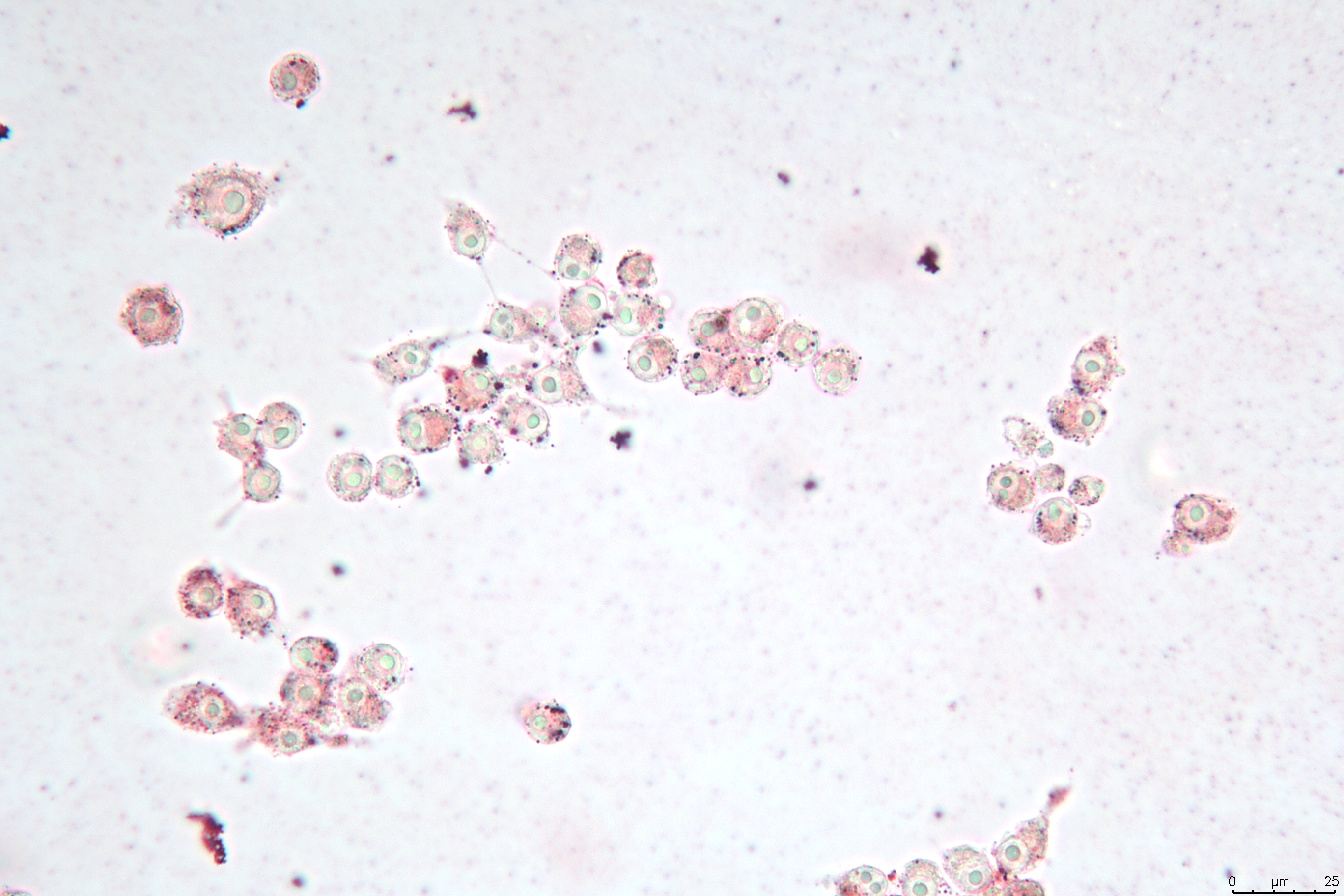

Supplement: Figure 4—source data 3. [file elife-80494-fig4-data3.zip › Figure 4G-1/Project_c-40-1.tif]

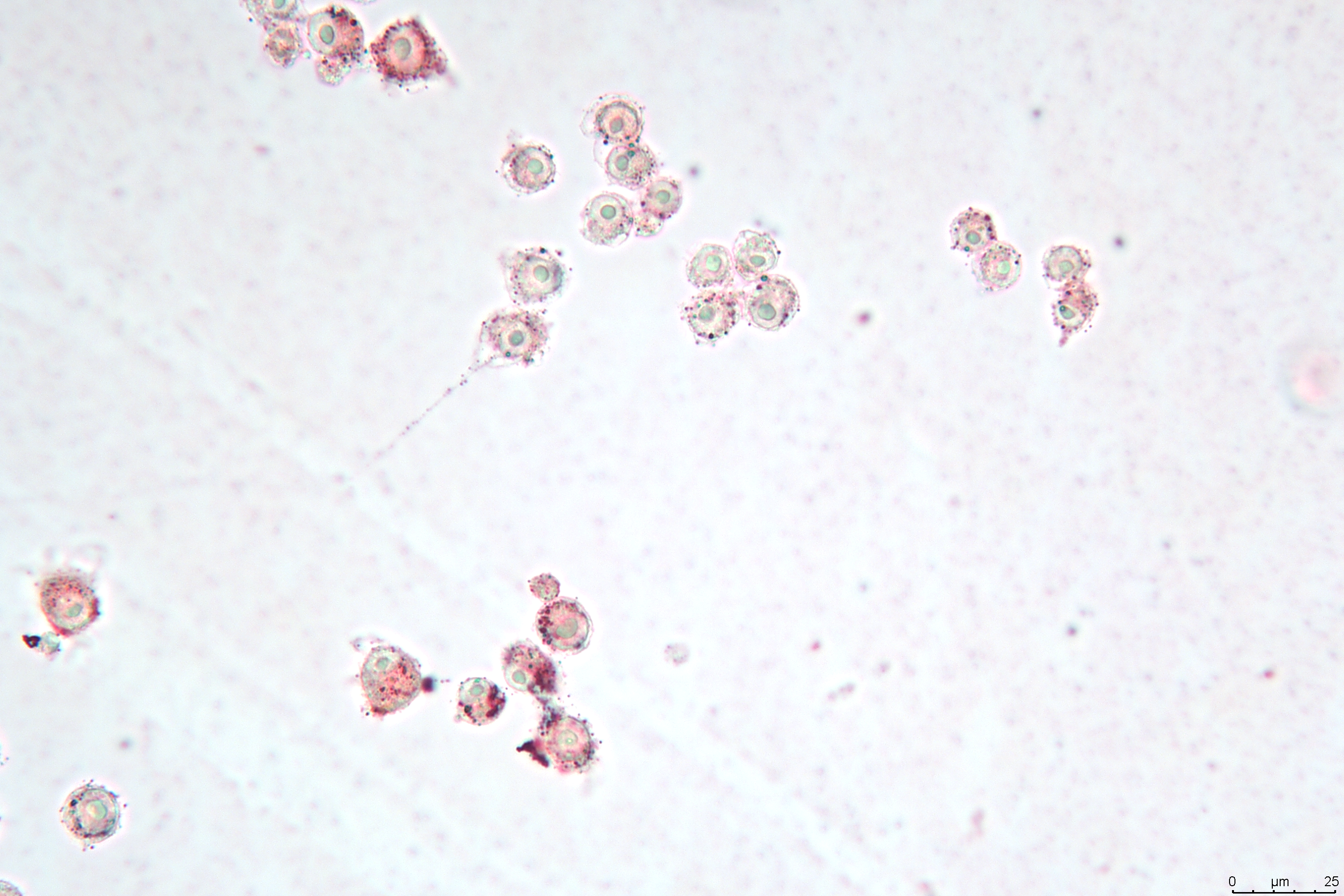

Supplement: Figure 4—source data 3. [file elife-80494-fig4-data3.zip › Figure 4G-1/Project_c-40-2.tif]

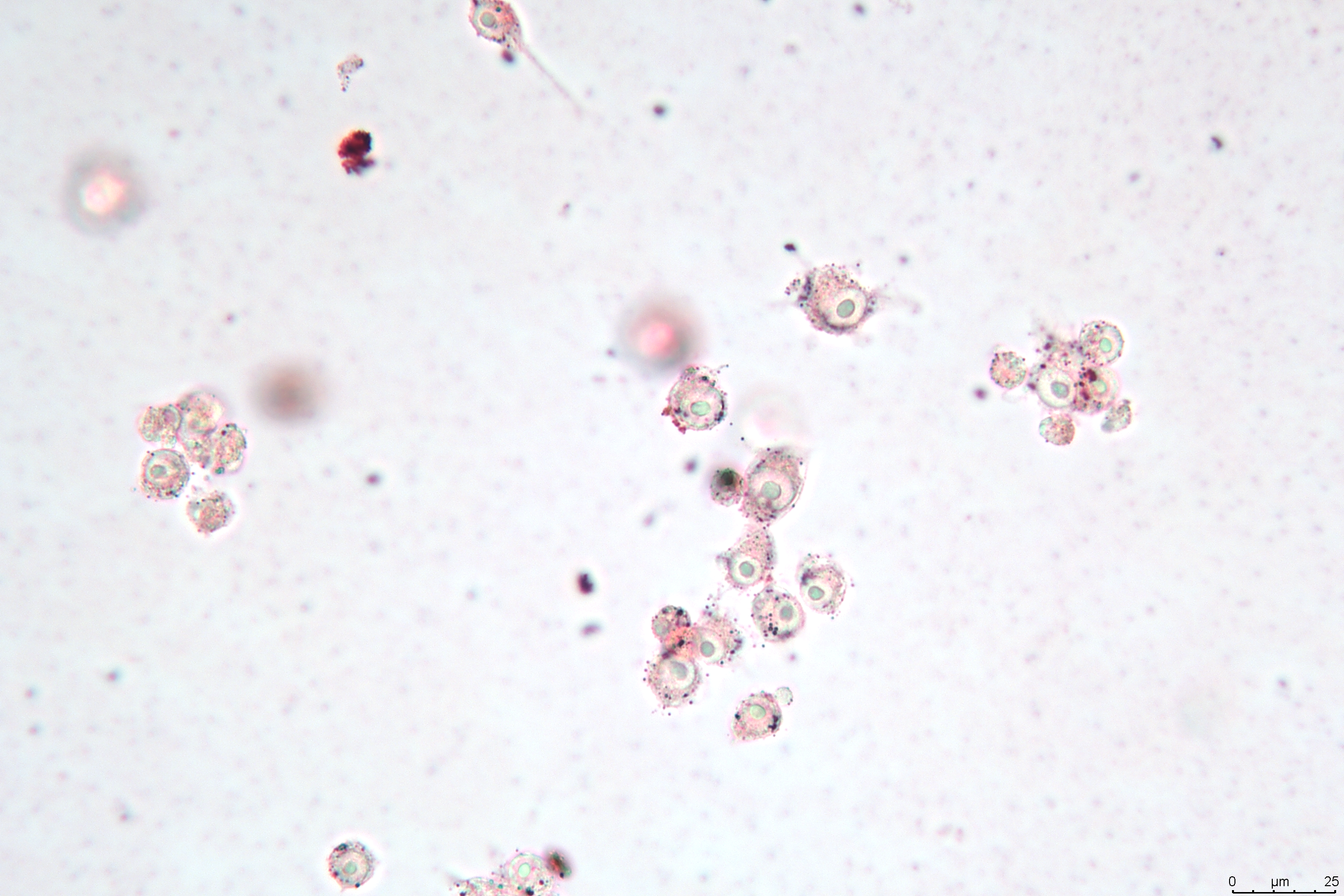

Supplement: Figure 4—source data 3. [file elife-80494-fig4-data3.zip › Figure 4G-1/Project_c-40-3.tif]

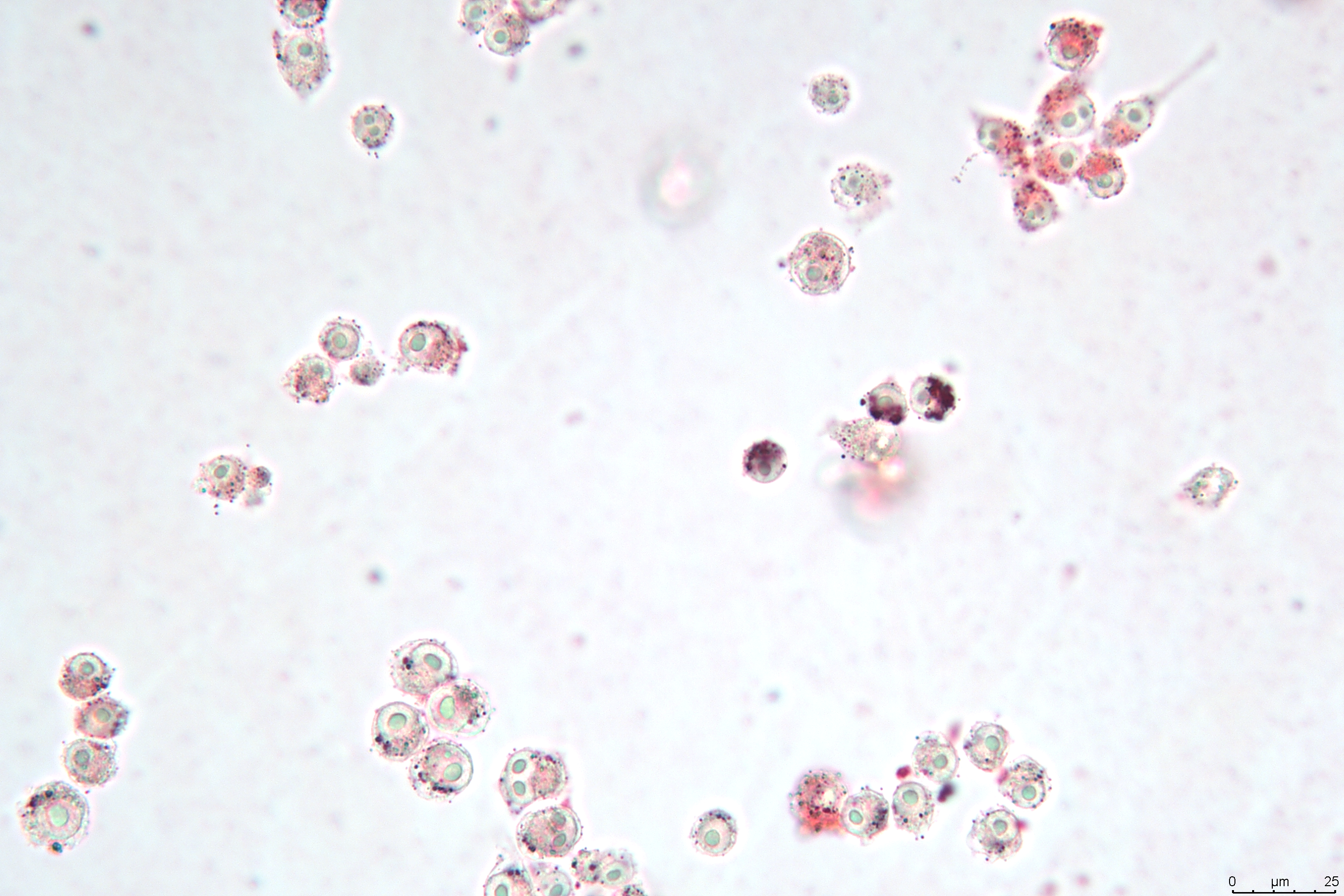

Supplement: Figure 4—source data 3. [file elife-80494-fig4-data3.zip › Figure 4G-1/Project_c-40-4.tif]

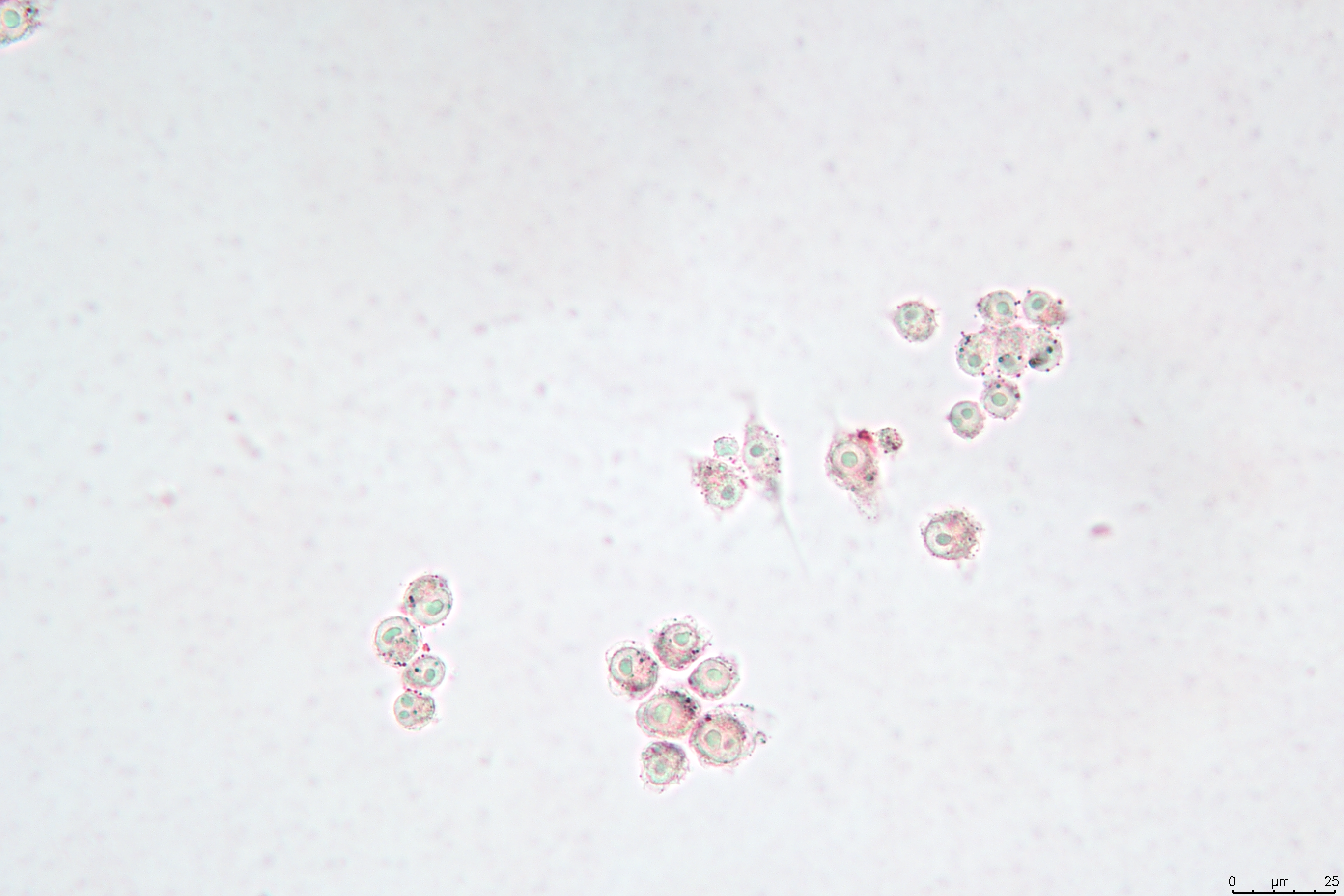

Supplement: Figure 4—source data 3. [file elife-80494-fig4-data3.zip › Figure 4G-1/Project_e-40-1.tif]

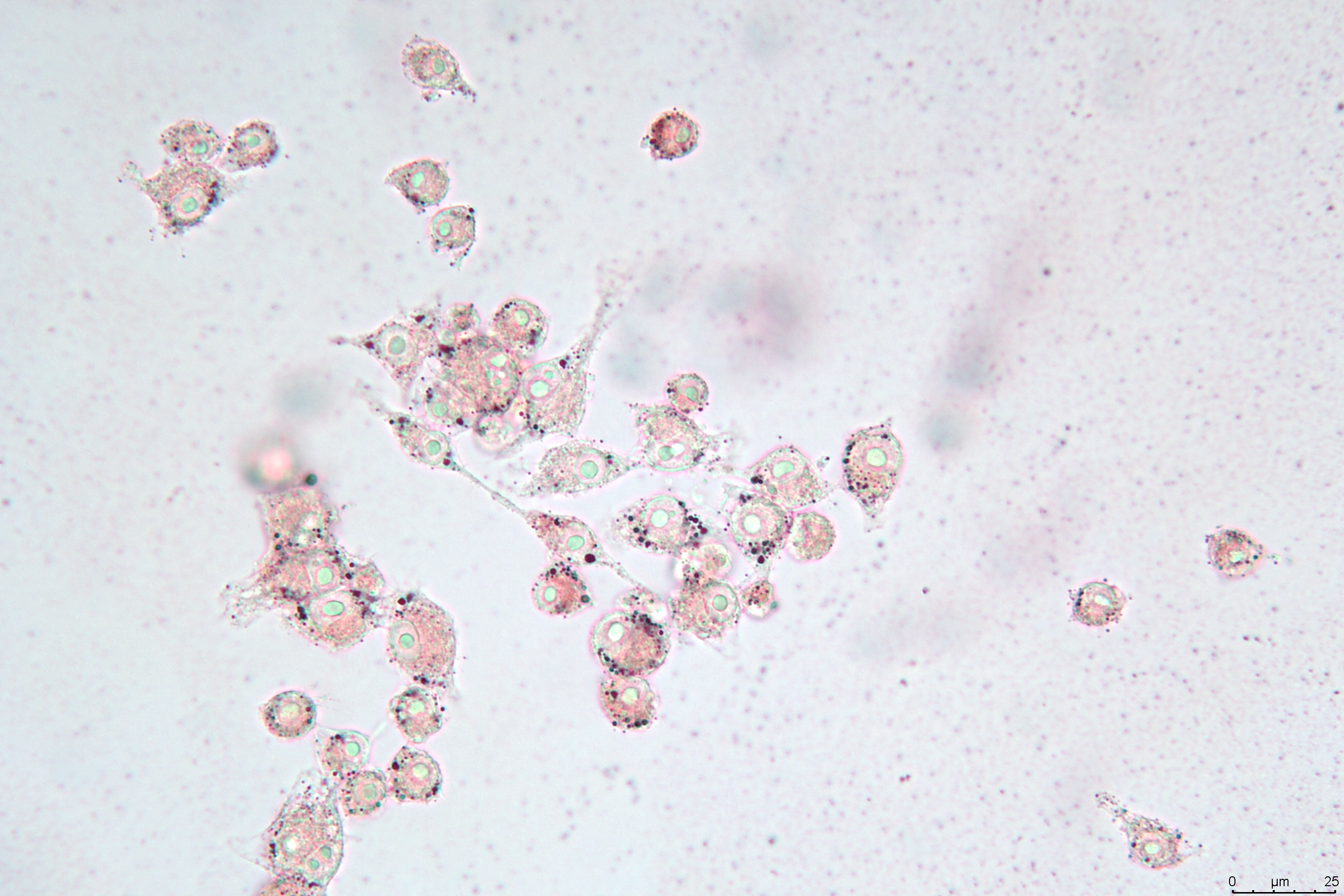

Supplement: Figure 4—source data 3. [file elife-80494-fig4-data3.zip › Figure 4G-1/Project_e-40-2.tif]

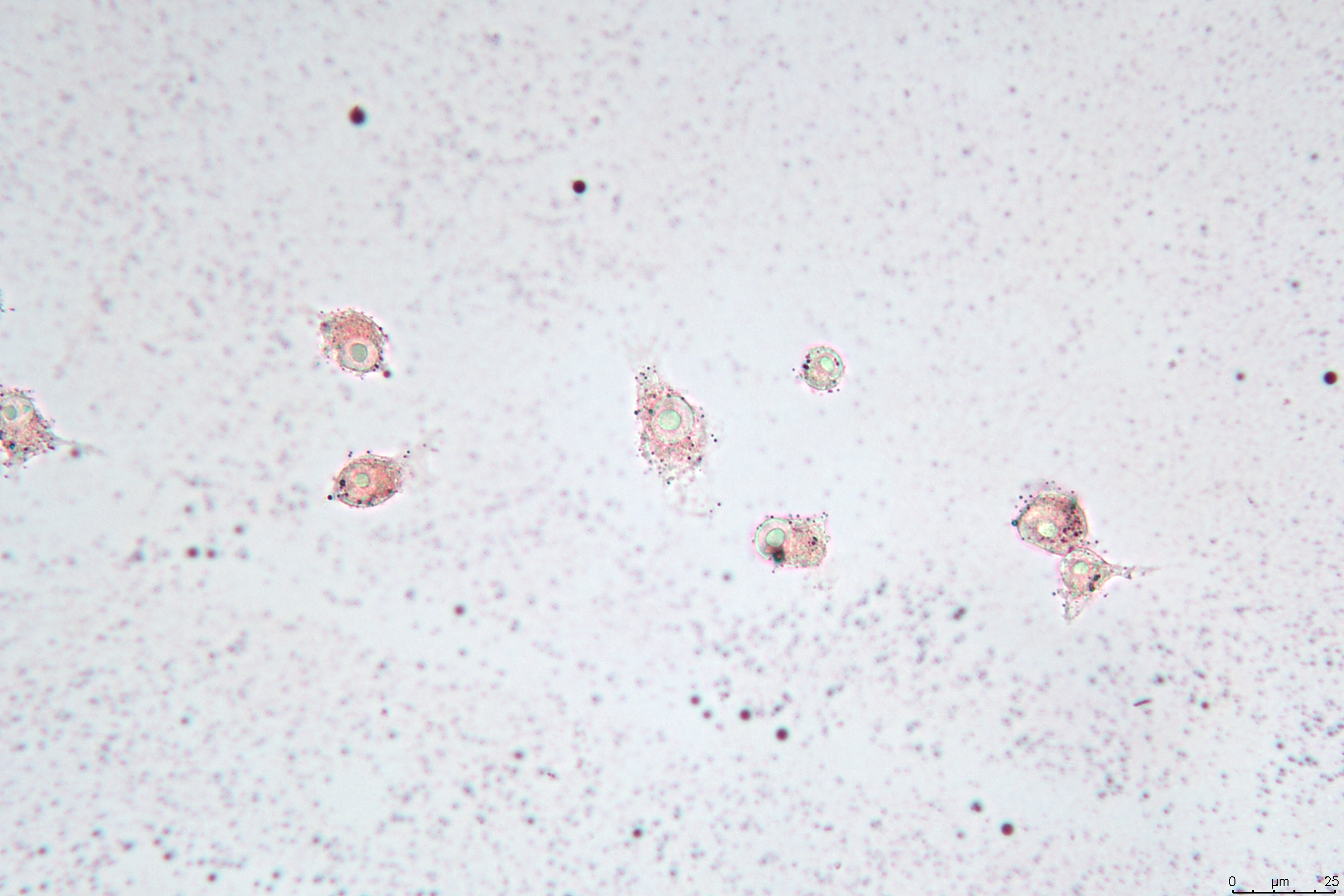

Supplement: Figure 4—source data 3. [file elife-80494-fig4-data3.zip › Figure 4G-1/Project_e-40-3.tif]

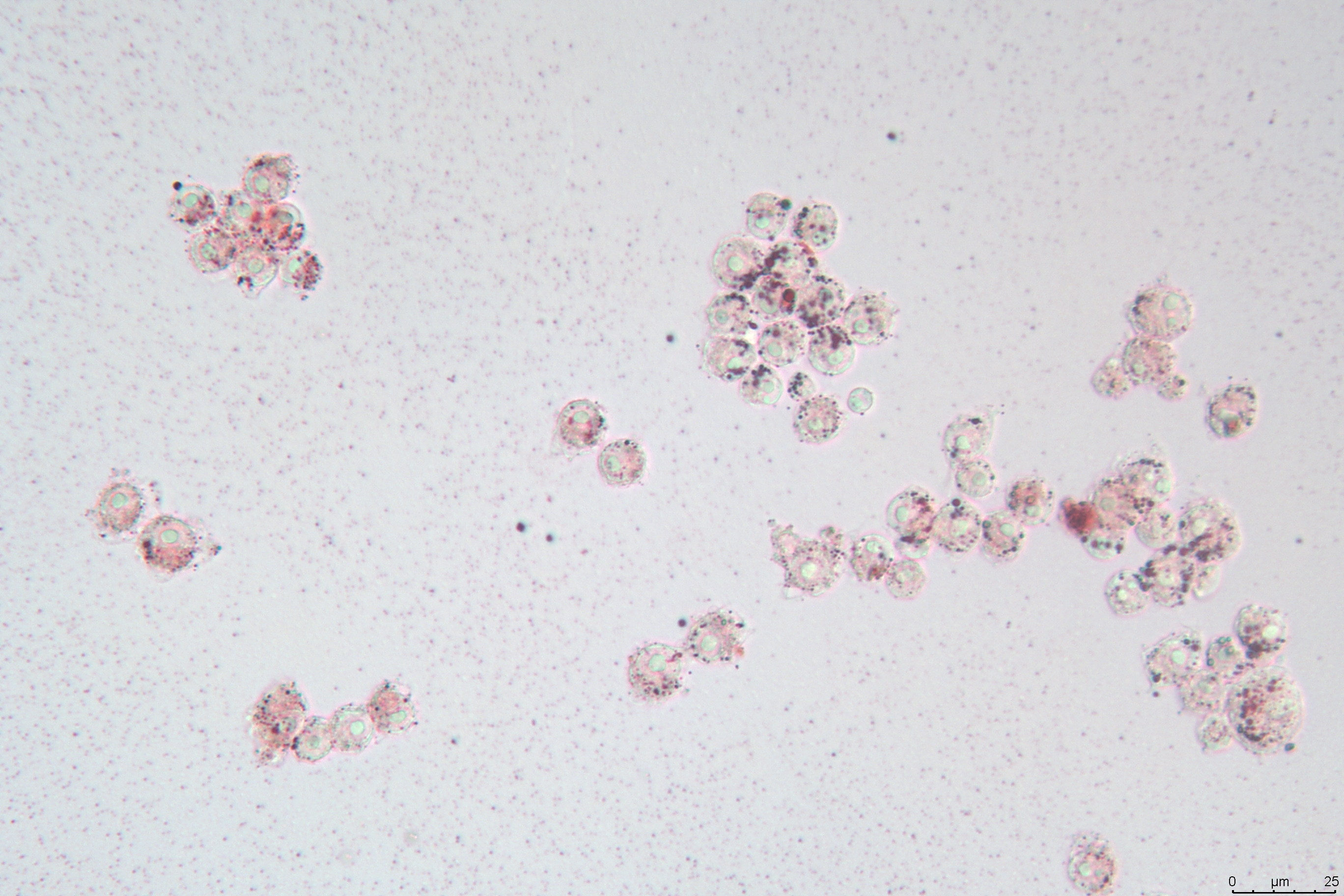

Supplement: Figure 4—source data 3. [file elife-80494-fig4-data3.zip › Figure 4G-1/Project_e-40-4.tif]

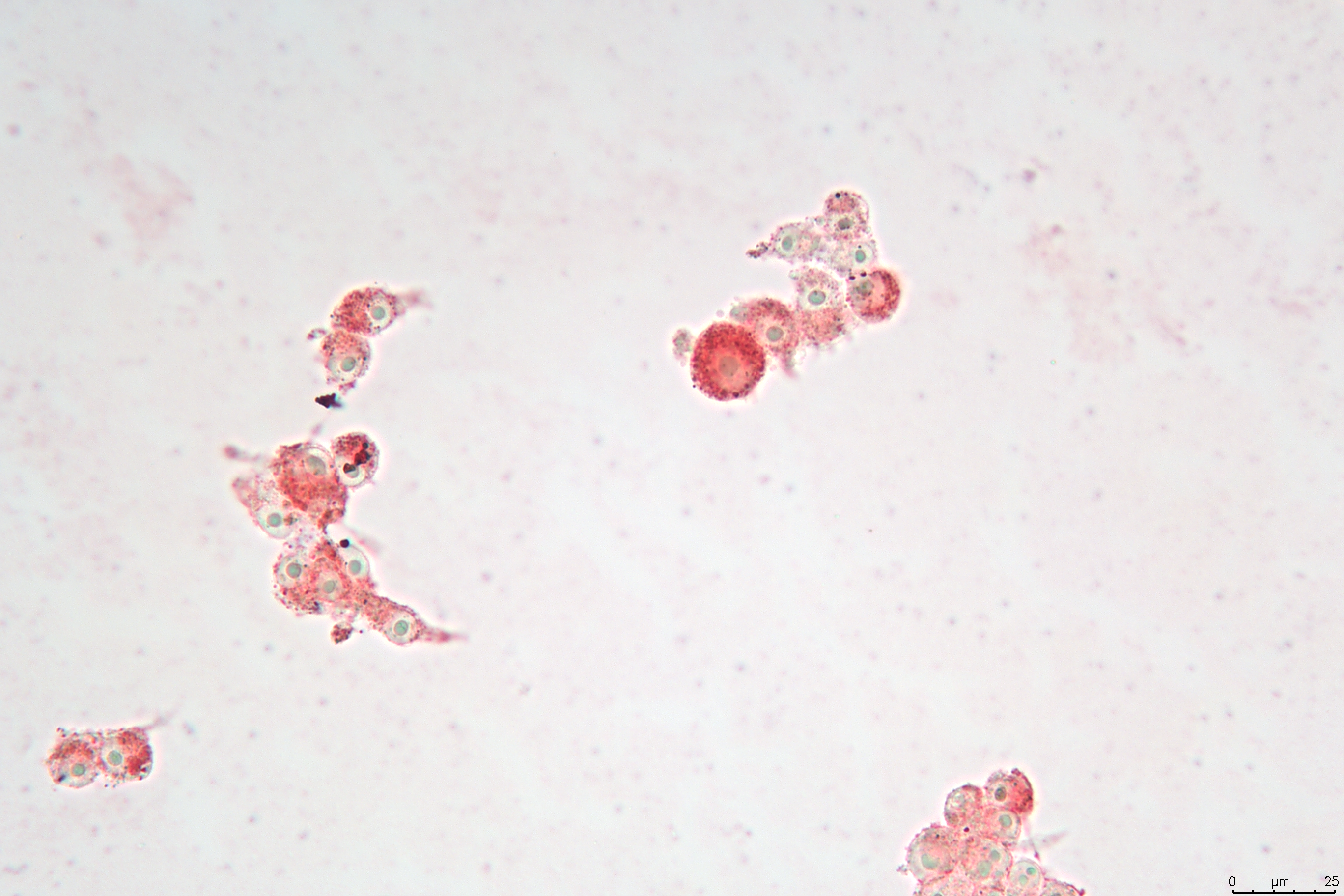

Supplement: Figure 4—source data 3. [file elife-80494-fig4-data3.zip › Figure 4G-1/Project_ef-40-1.tif]

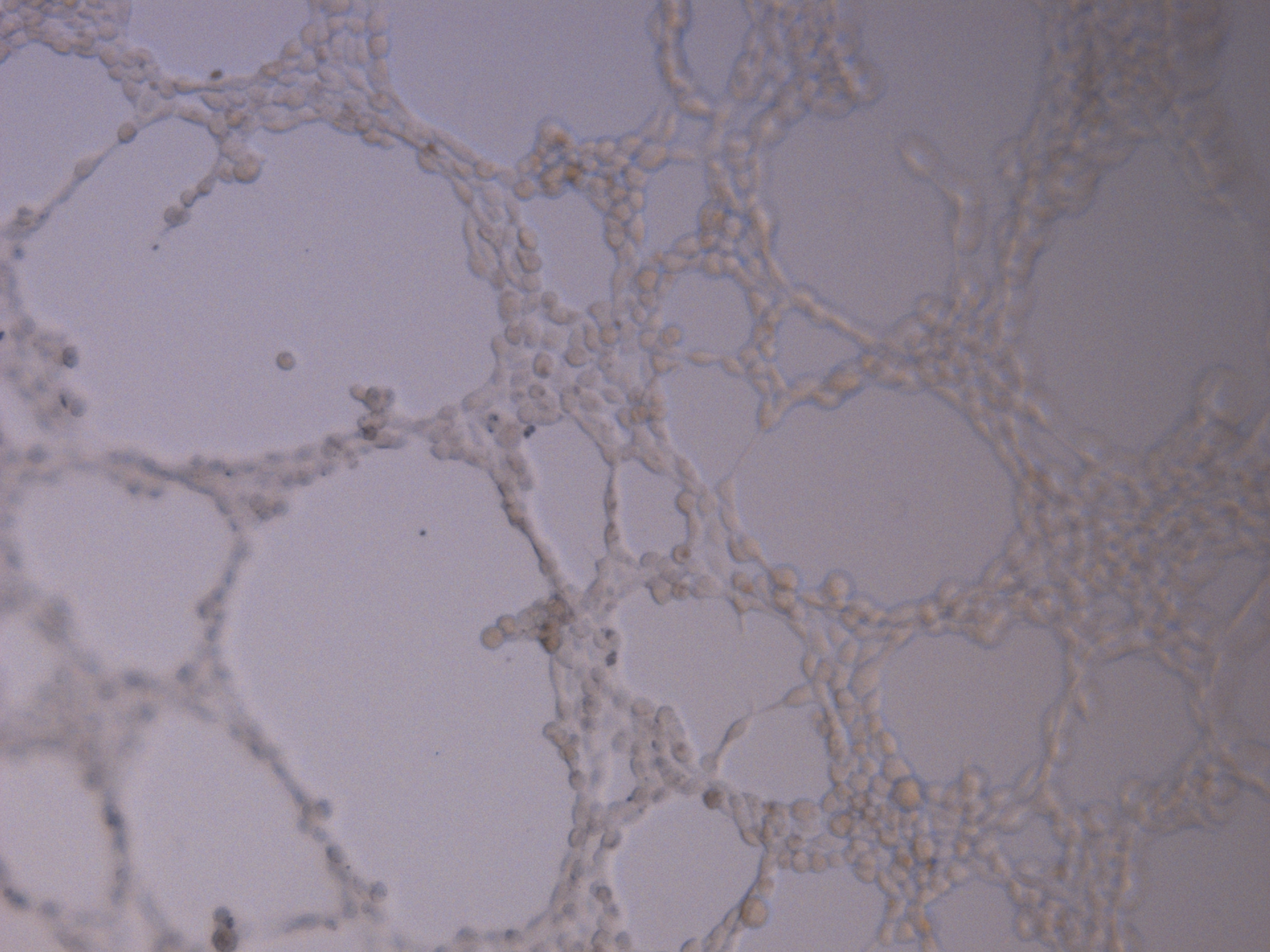

Supplement: Figure 4—source data 4. [file elife-80494-fig4-data4.zip › Fig4H/E2+FAC-1.tif]

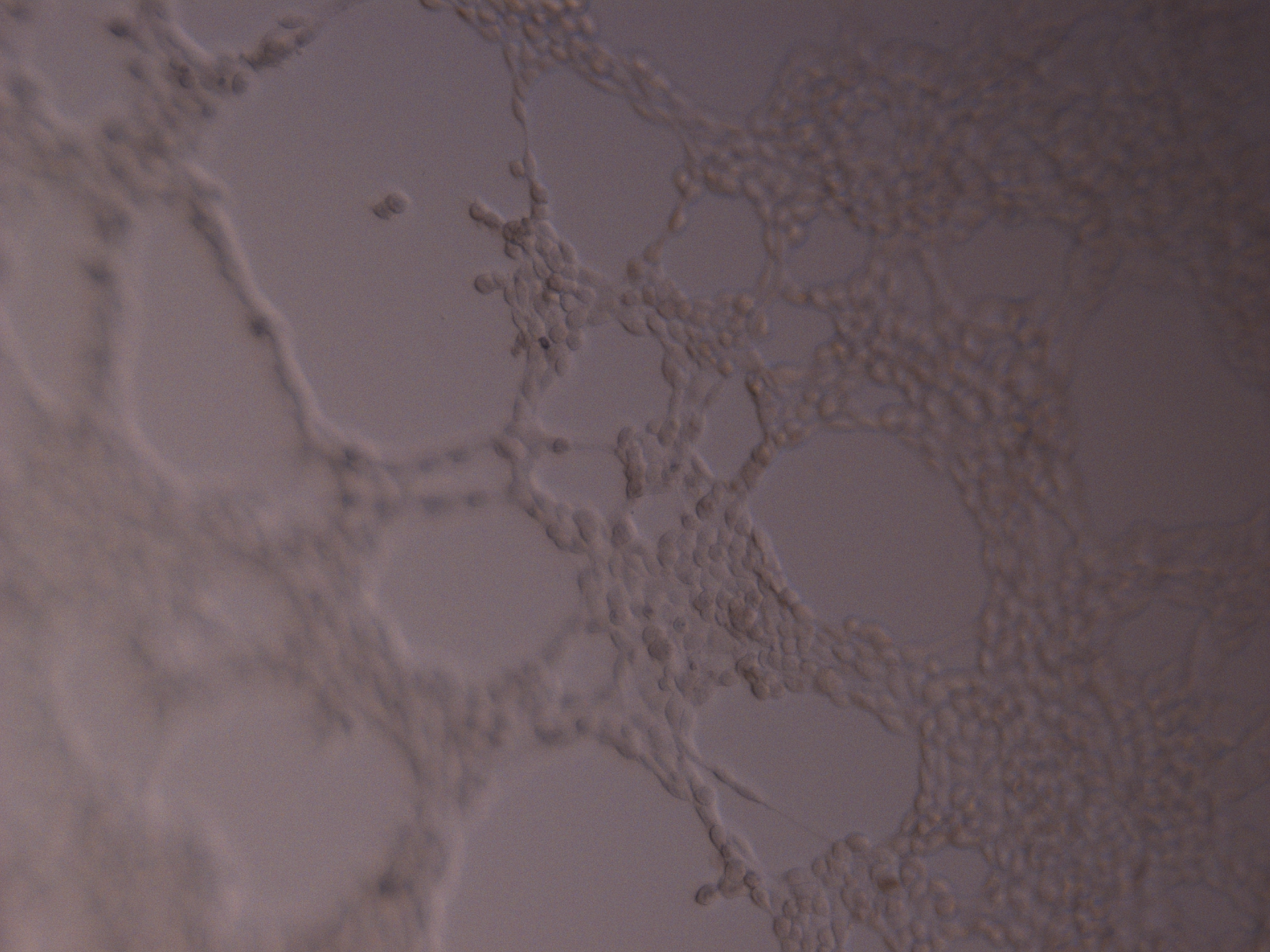

Supplement: Figure 4—source data 4. [file elife-80494-fig4-data4.zip › Fig4H/E2+FAC-2.tif]

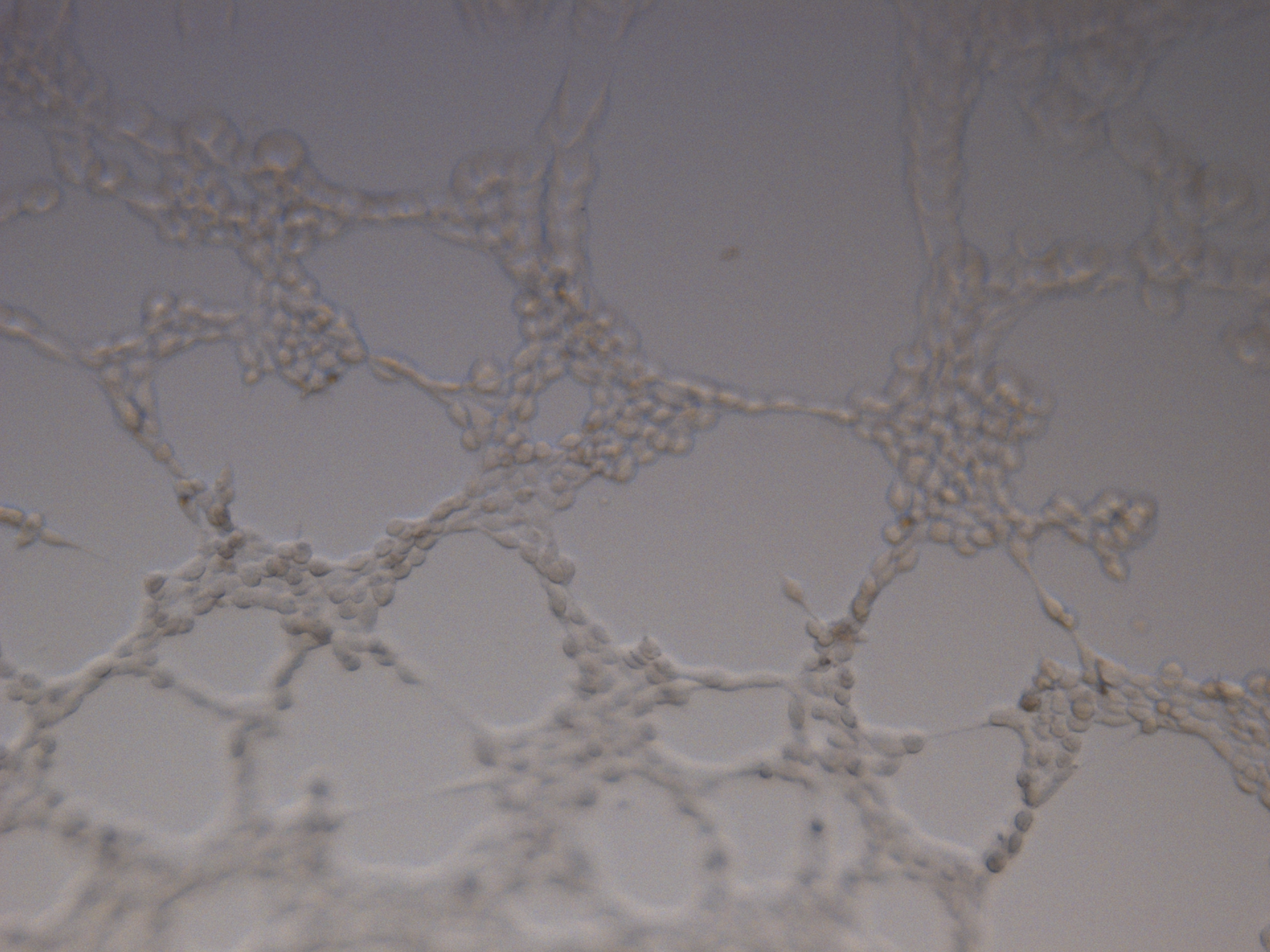

Supplement: Figure 4—source data 4. [file elife-80494-fig4-data4.zip › Fig4H/E2+FAC-3.tif]

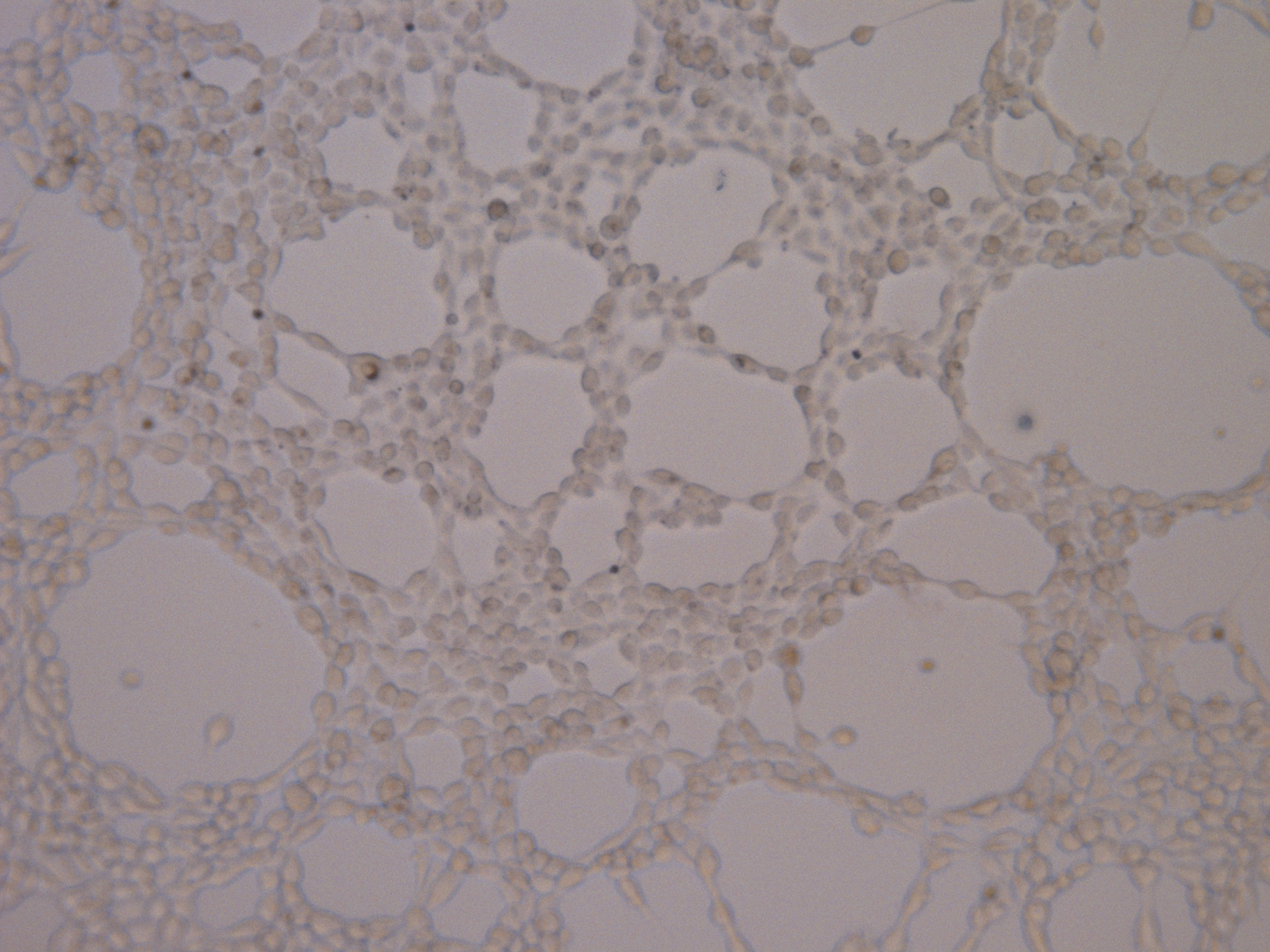

Supplement: Figure 4—source data 4. [file elife-80494-fig4-data4.zip › Fig4H/E2-1.tif]

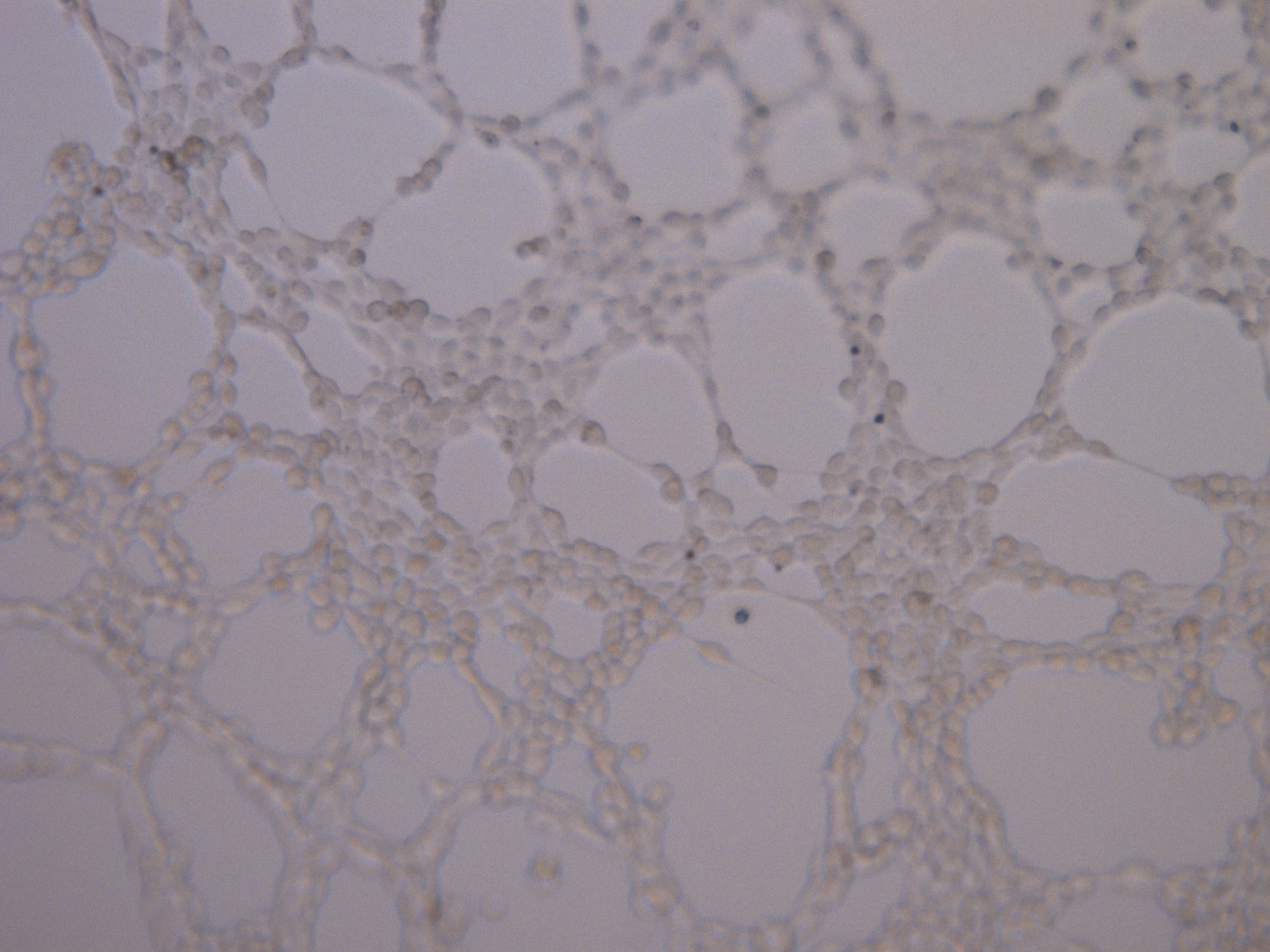

Supplement: Figure 4—source data 4. [file elife-80494-fig4-data4.zip › Fig4H/E2-2.tif]

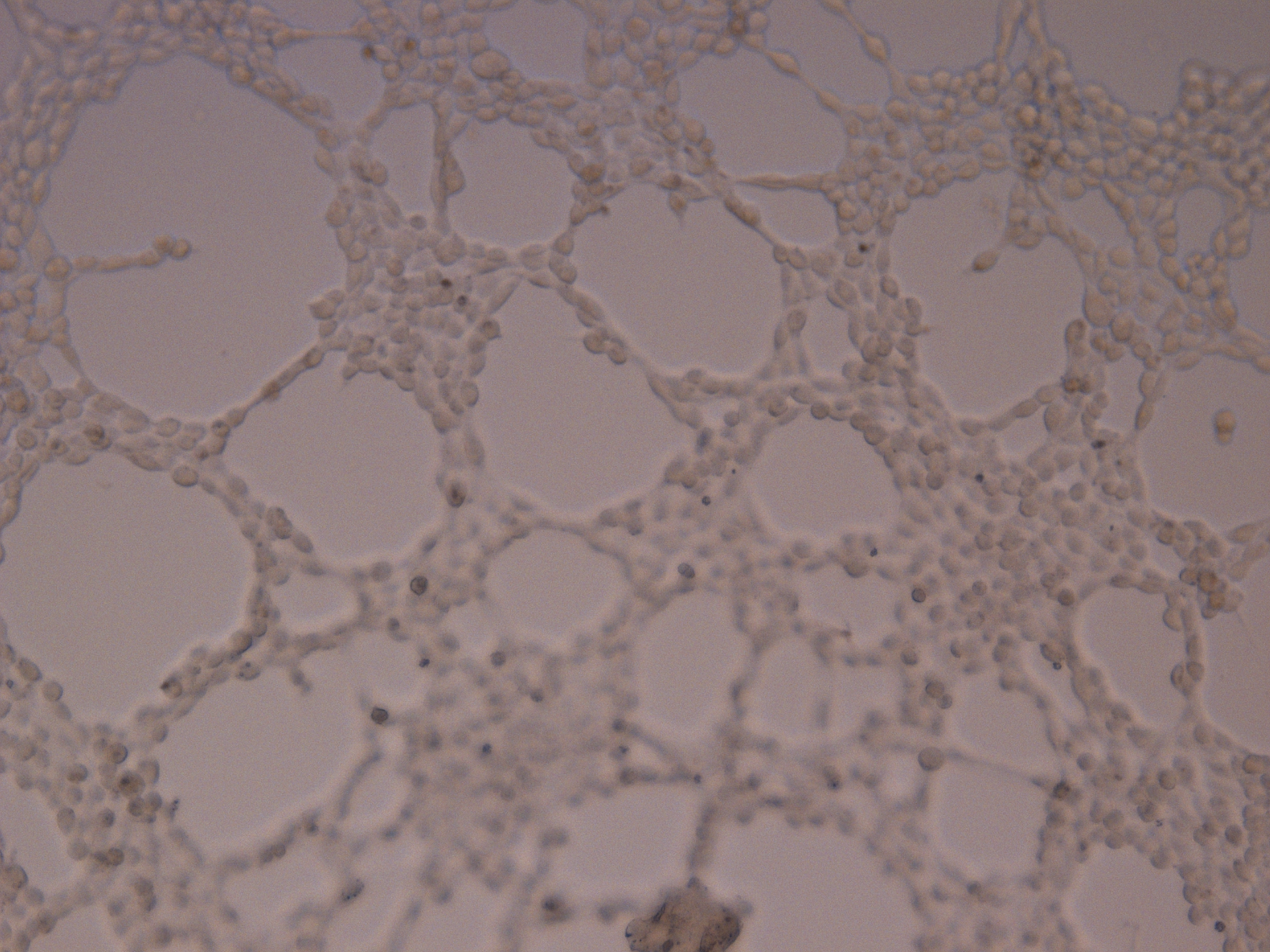

Supplement: Figure 4—source data 4. [file elife-80494-fig4-data4.zip › Fig4H/E2-3.tif]

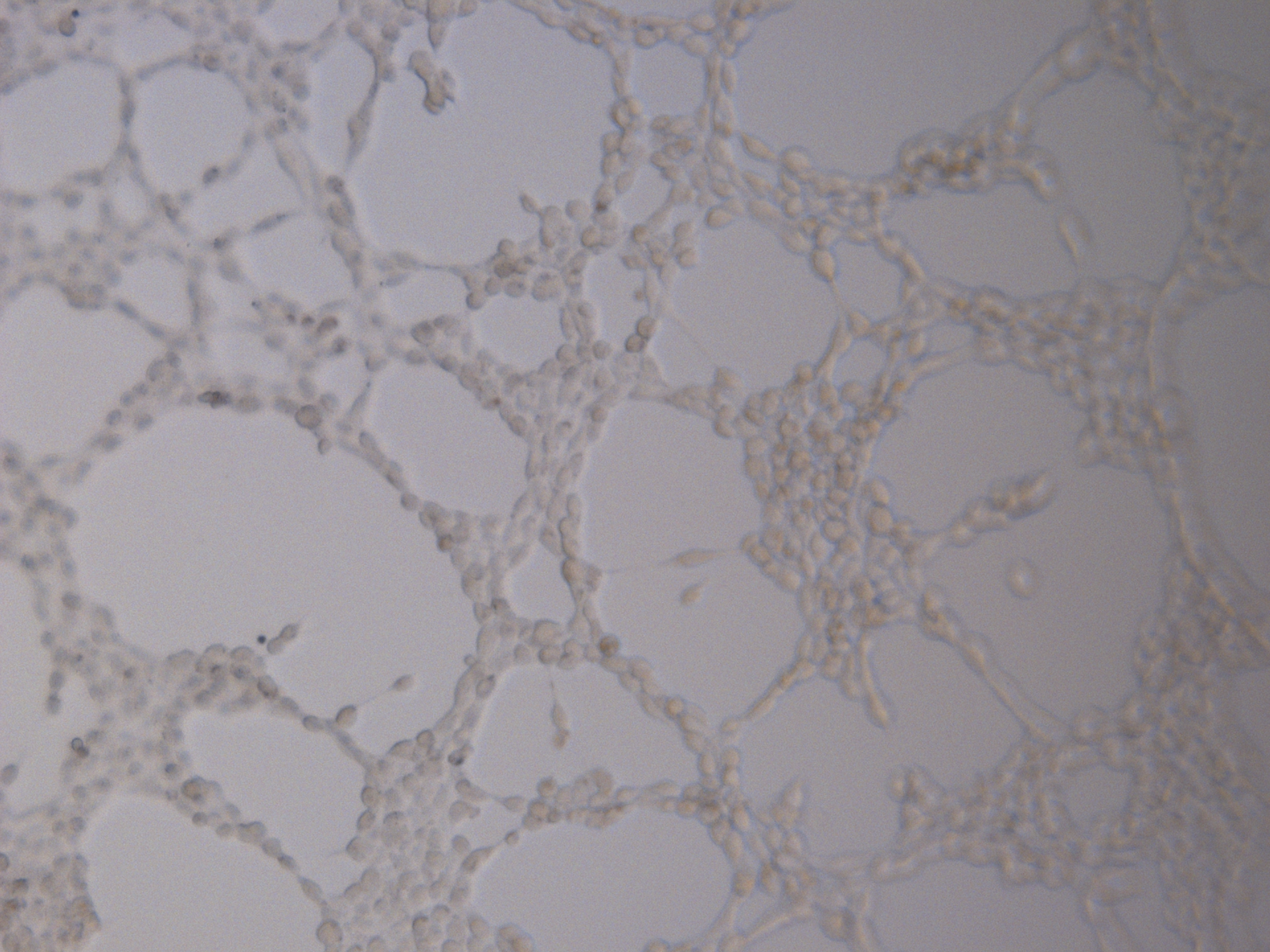

Supplement: Figure 4—source data 4. [file elife-80494-fig4-data4.zip › Fig4H/FAC-1.tif]

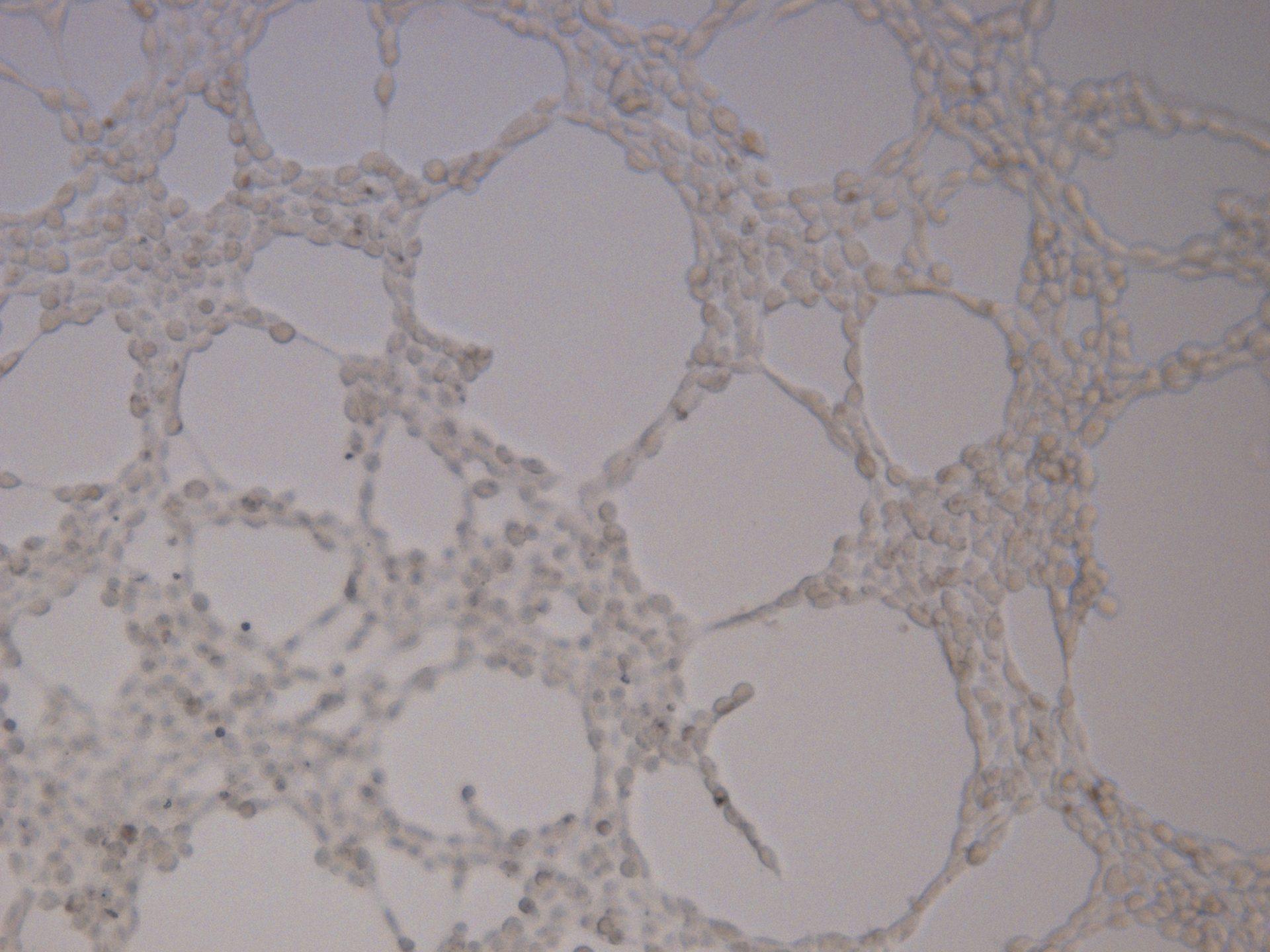

Supplement: Figure 4—source data 4. [file elife-80494-fig4-data4.zip › Fig4H/FAC-2.tif]

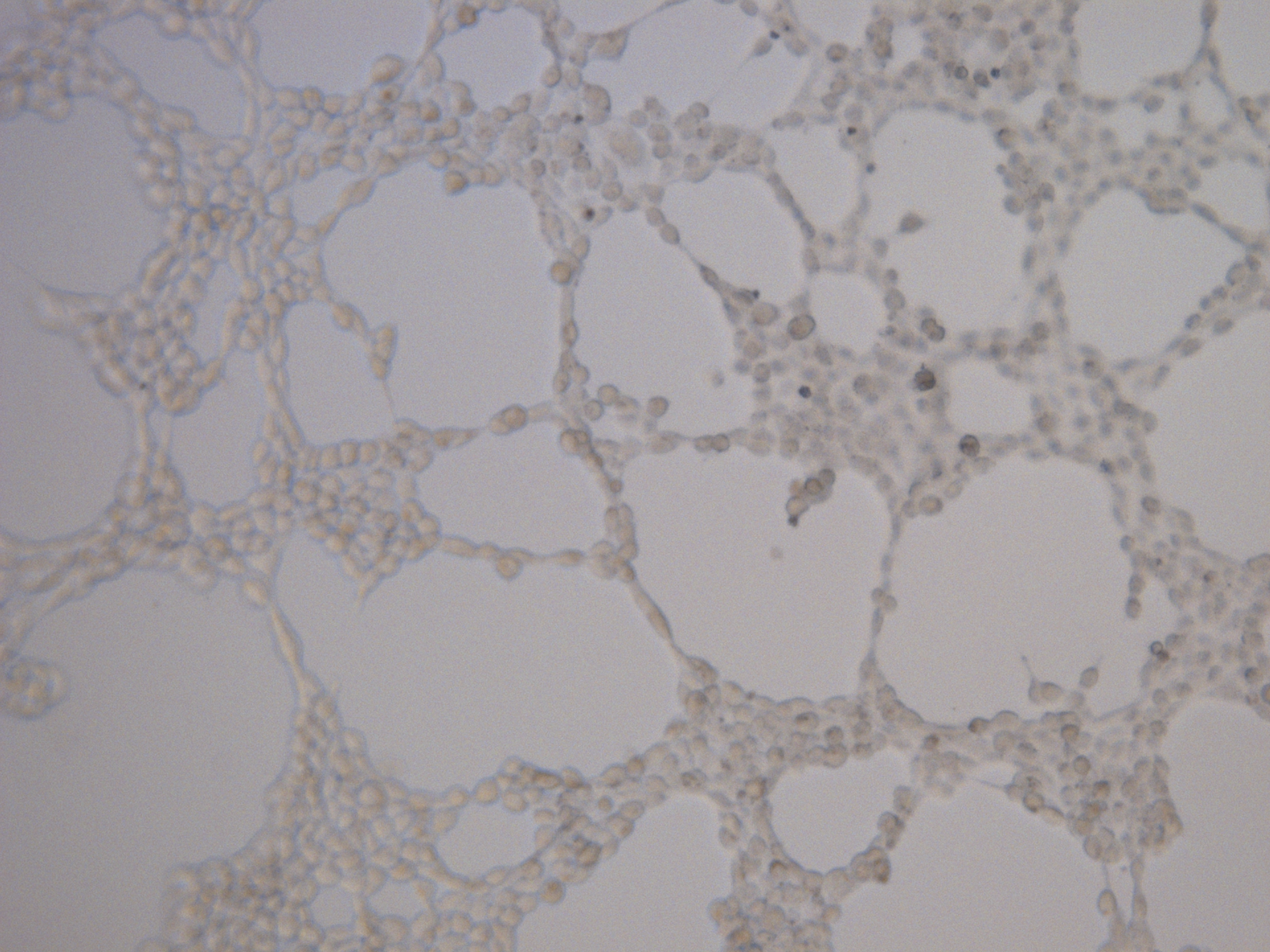

Supplement: Figure 4—source data 4. [file elife-80494-fig4-data4.zip › Fig4H/FAC-3.tif]

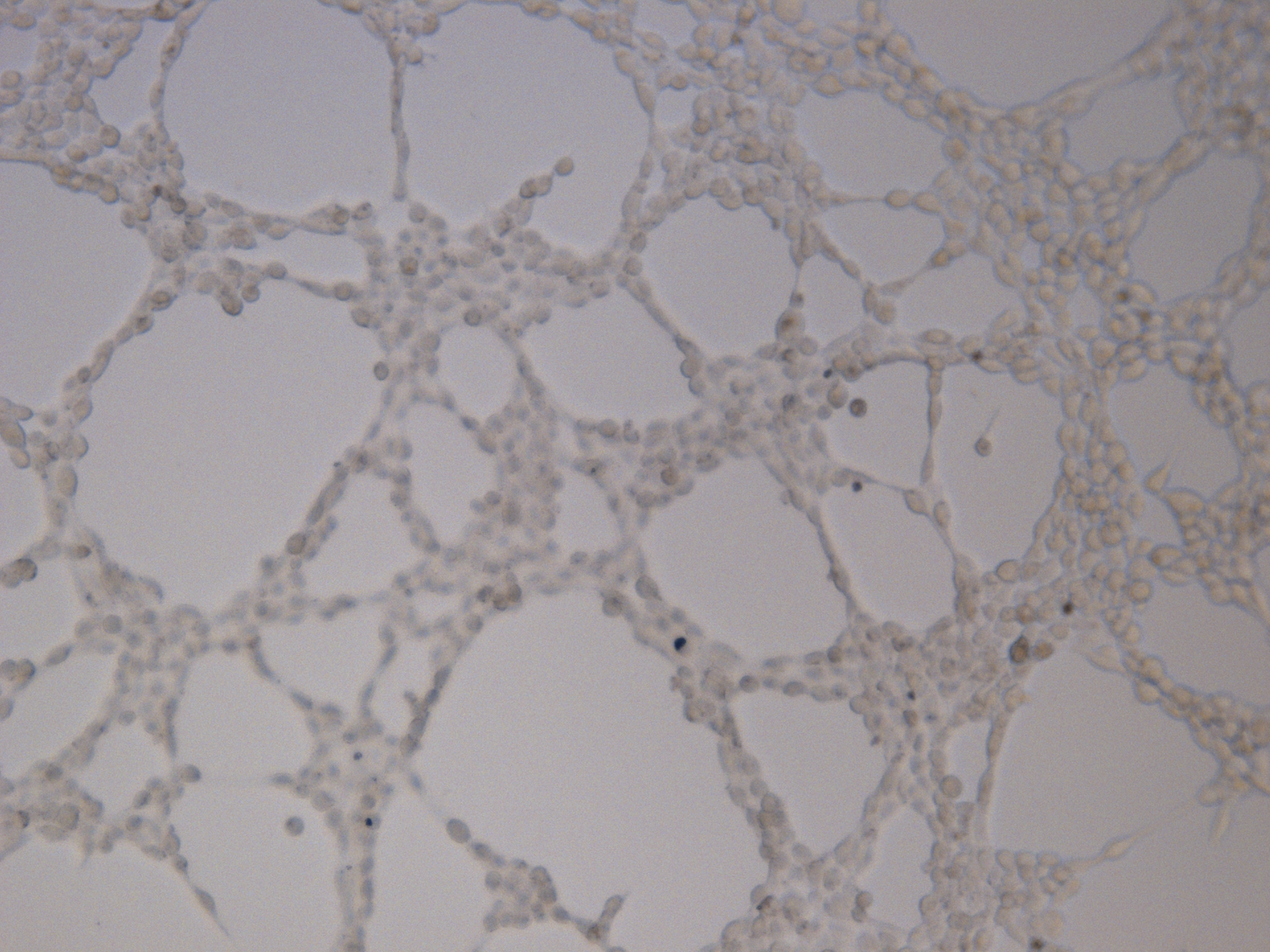

Supplement: Figure 4—source data 4. [file elife-80494-fig4-data4.zip › Fig4H/SHAM-1.tif]

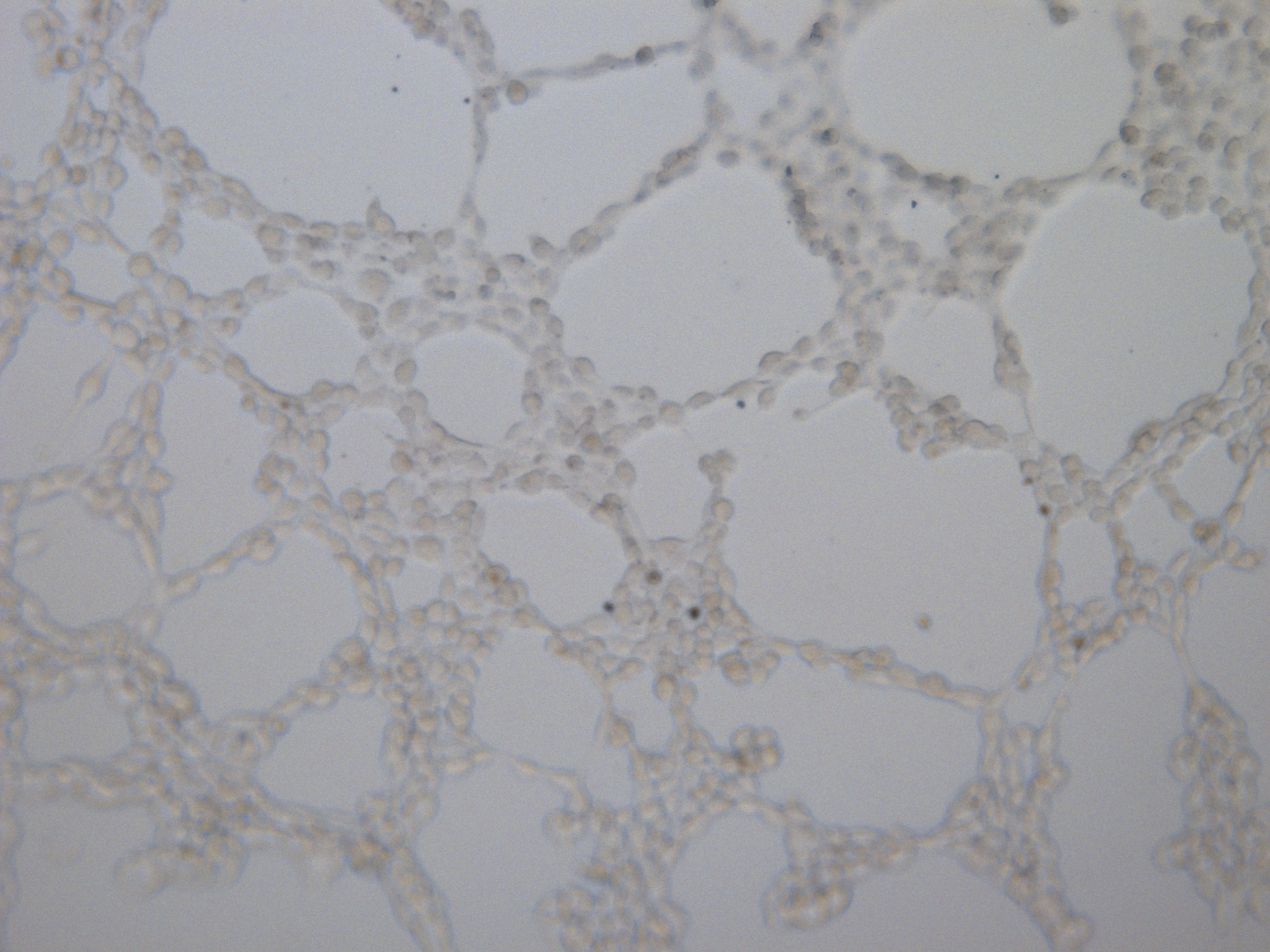

Supplement: Figure 4—source data 4. [file elife-80494-fig4-data4.zip › Fig4H/SHAM-2.tif]

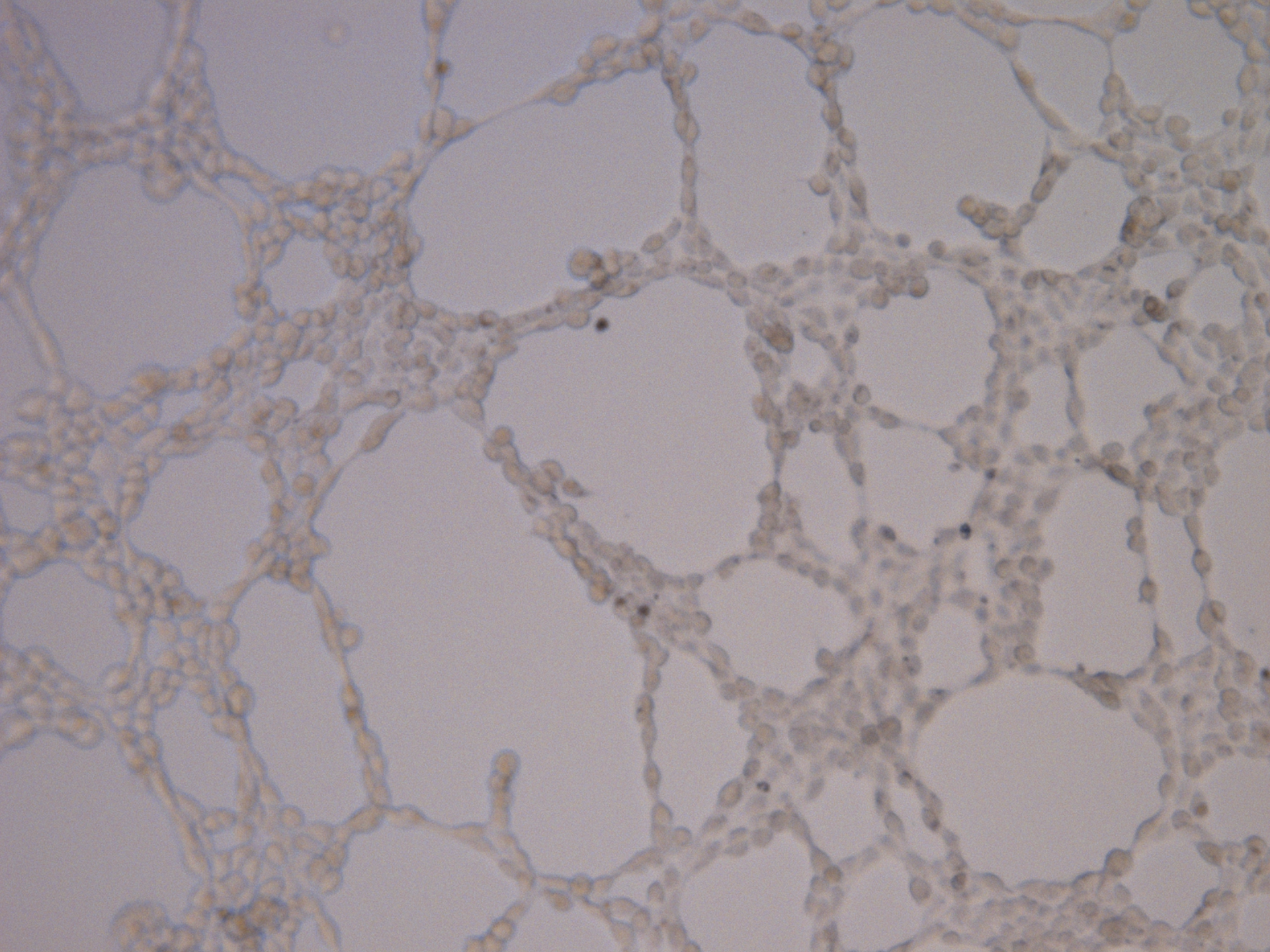

Supplement: Figure 4—source data 4. [file elife-80494-fig4-data4.zip › Fig4H/SHAM-3.tif]

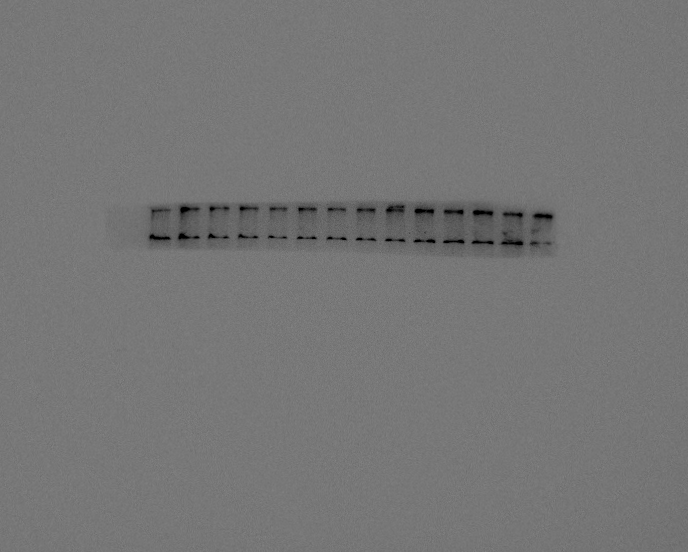

Supplement: Figure 5—source data 1. [file elife-80494-fig5-data1.zip › Fig5/Fig5A/ABCA1/10.tif]

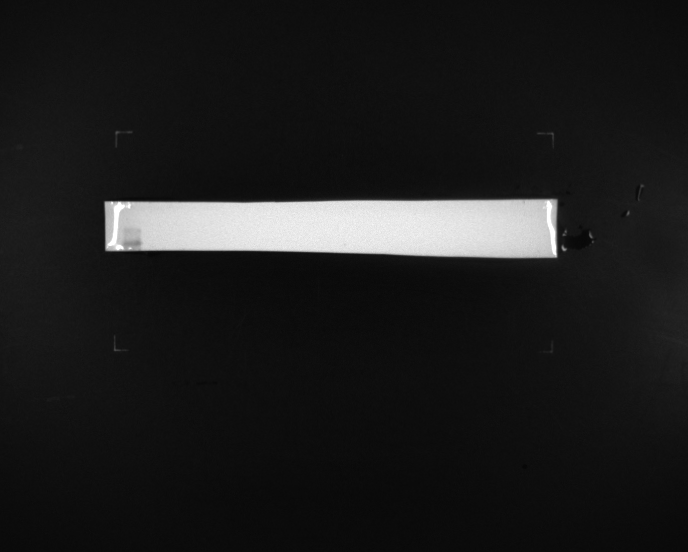

Supplement: Figure 5—source data 1. [file elife-80494-fig5-data1.zip › Fig5/Fig5A/ABCA1/MK.tif]

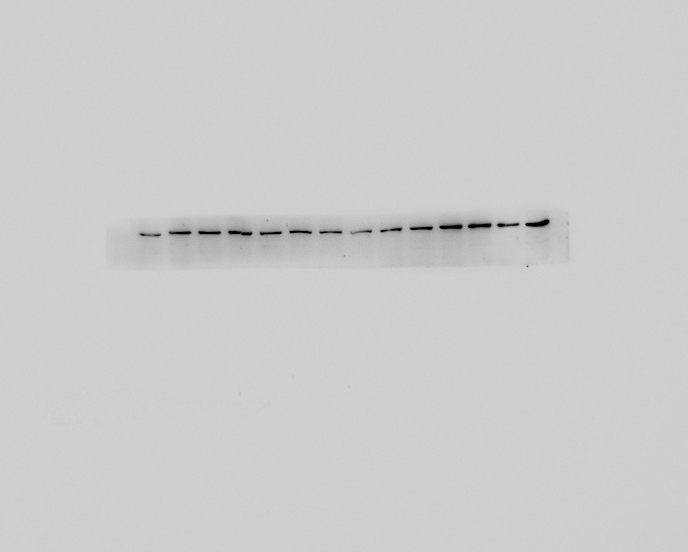

Supplement: Figure 5—source data 1. [file elife-80494-fig5-data1.zip › Fig5/Fig5A/ERa┴/30.tif]

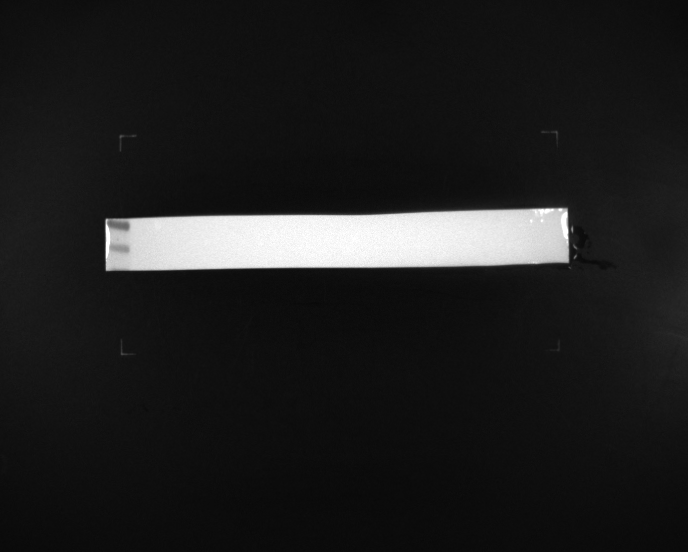

Supplement: Figure 5—source data 1. [file elife-80494-fig5-data1.zip › Fig5/Fig5A/ERa┴/MK.tif]

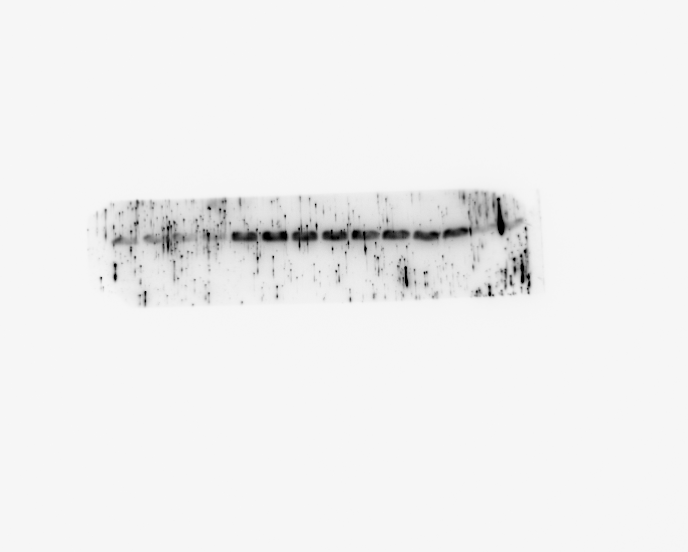

Supplement: Figure 5—source data 1. [file elife-80494-fig5-data1.zip › Fig5/Fig5A/Ftl/10.tif]

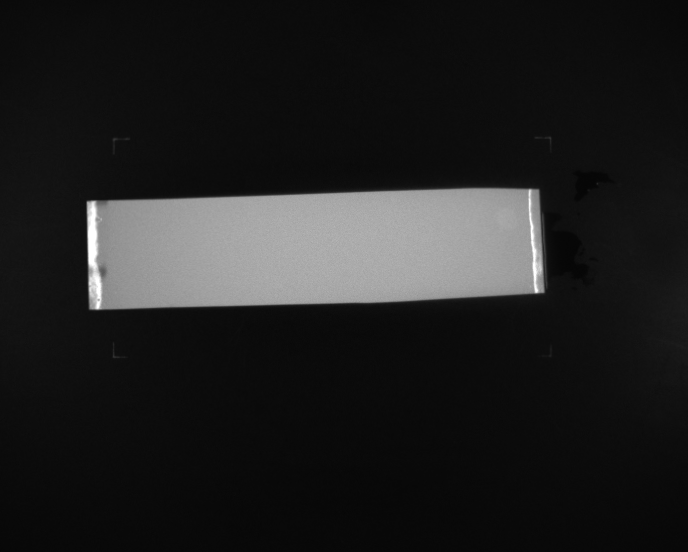

Supplement: Figure 5—source data 1. [file elife-80494-fig5-data1.zip › Fig5/Fig5A/Ftl/MK.tif]

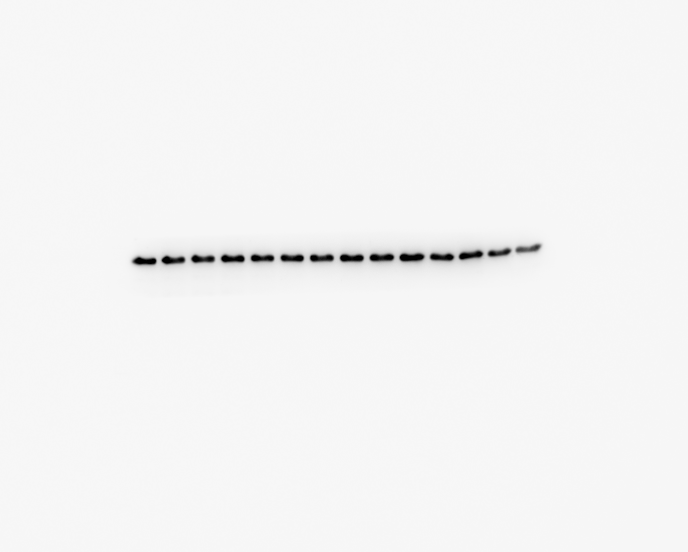

Supplement: Figure 5—source data 1. [file elife-80494-fig5-data1.zip › Fig5/Fig5A/GAPDH/60.tif]

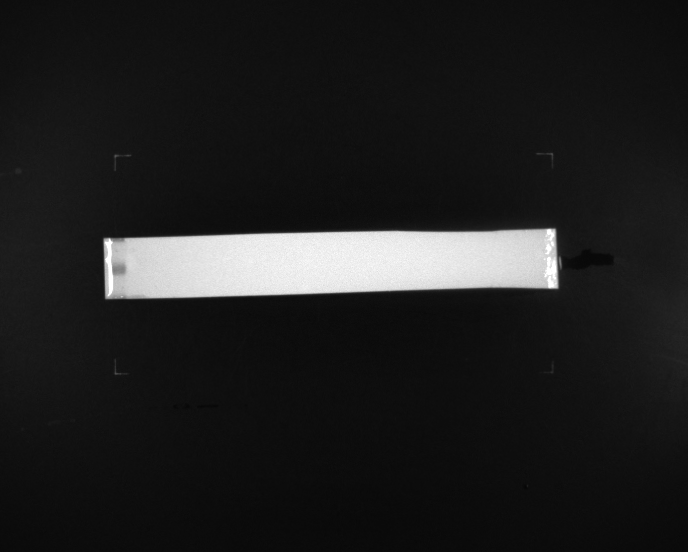

Supplement: Figure 5—source data 1. [file elife-80494-fig5-data1.zip › Fig5/Fig5A/GAPDH/MK.tif]

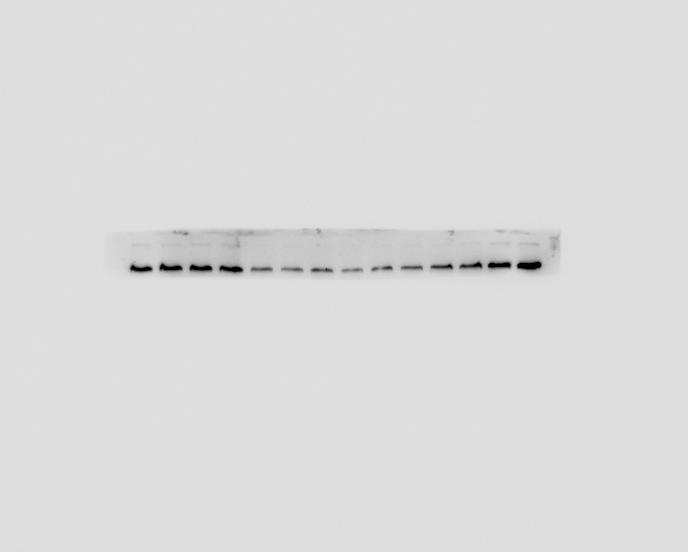

Supplement: Figure 5—source data 1. [file elife-80494-fig5-data1.zip › Fig5/Fig5A/TfR1/30.tif]

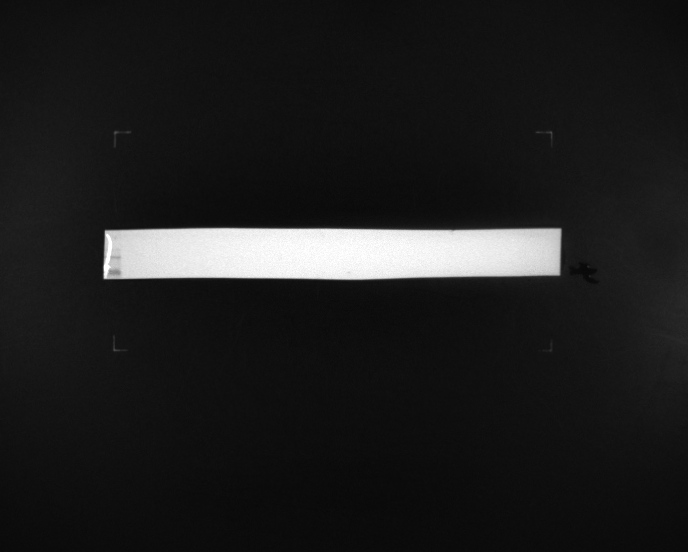

Supplement: Figure 5—source data 1. [file elife-80494-fig5-data1.zip › Fig5/Fig5A/TfR1/JMK.tif]

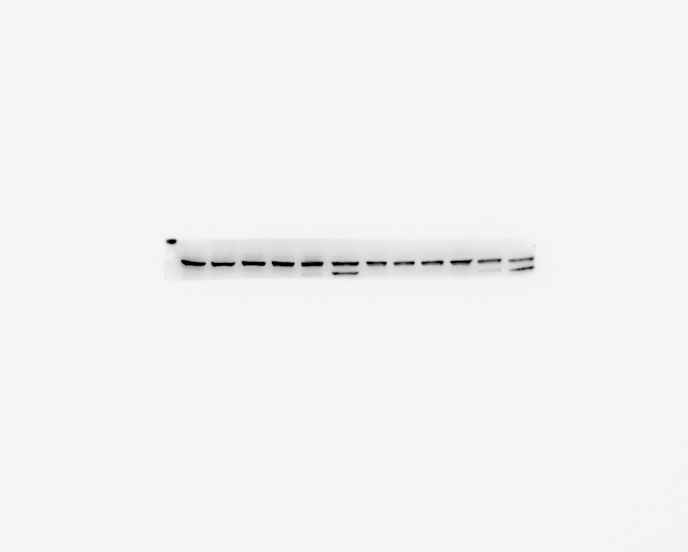

Supplement: Figure 5—source data 1. [file elife-80494-fig5-data1.zip › Fig5/Fig5B/ERa┴/30.tif]

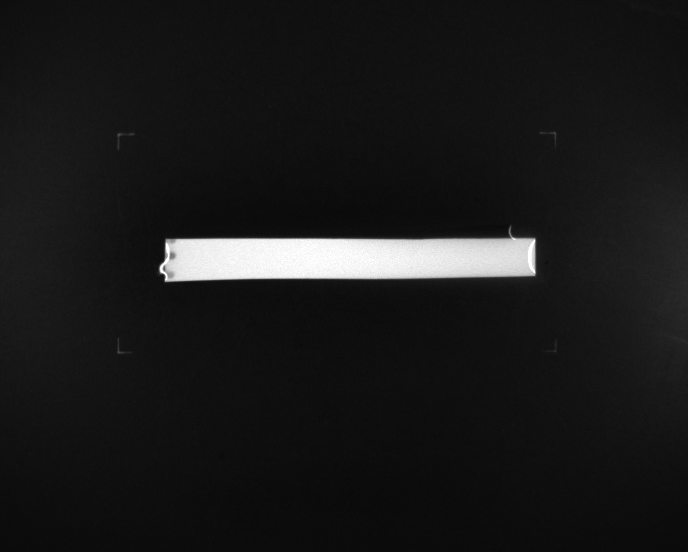

Supplement: Figure 5—source data 1. [file elife-80494-fig5-data1.zip › Fig5/Fig5B/ERa┴/mk.tif]

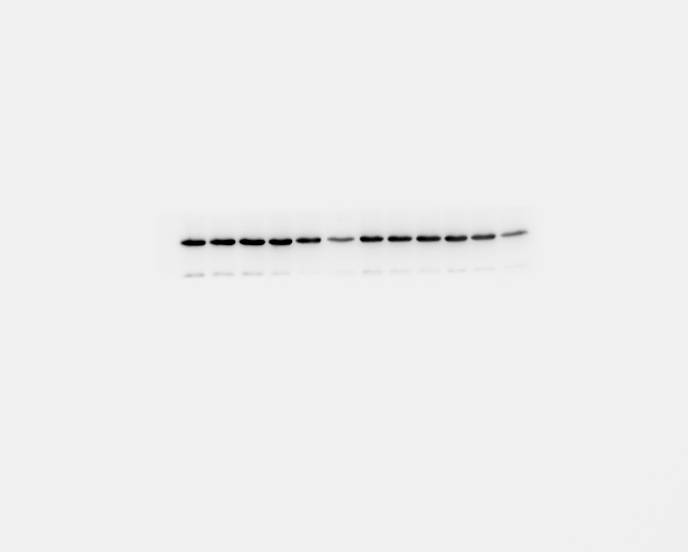

Supplement: Figure 5—source data 1. [file elife-80494-fig5-data1.zip › Fig5/Fig5B/GAPDH/10.tif]

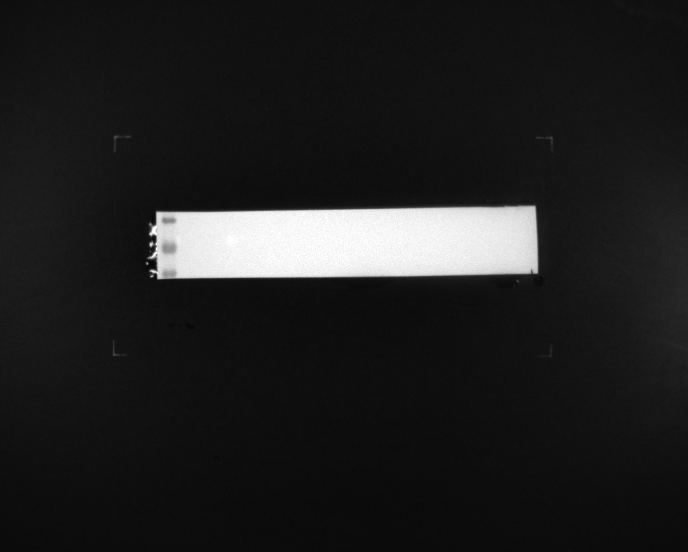

Supplement: Figure 5—source data 1. [file elife-80494-fig5-data1.zip › Fig5/Fig5B/GAPDH/mk.tif]

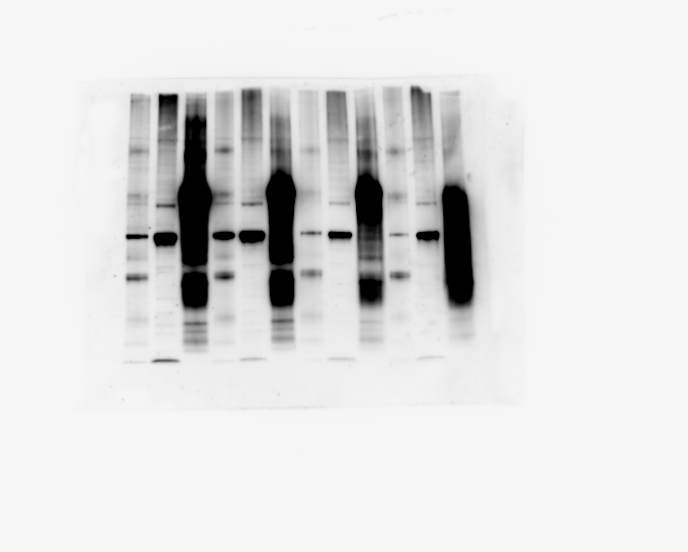

Supplement: Figure 5—source data 1. [file elife-80494-fig5-data1.zip › Fig5/Fig5C/LONG.tif]

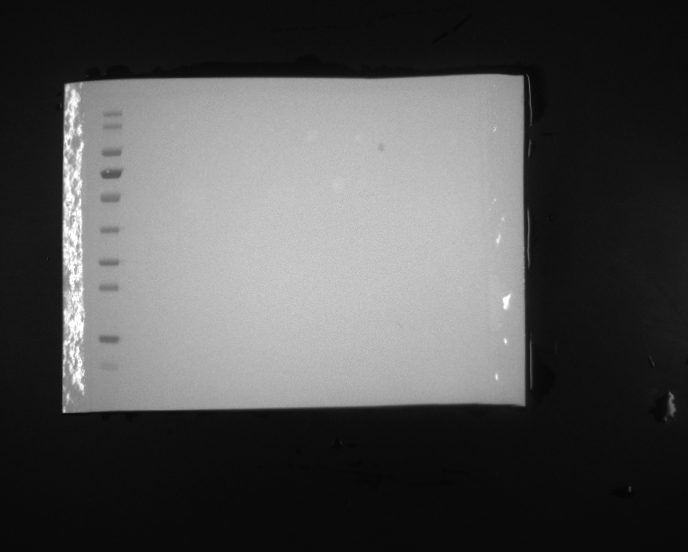

Supplement: Figure 5—source data 1. [file elife-80494-fig5-data1.zip › Fig5/Fig5C/MK.tif]

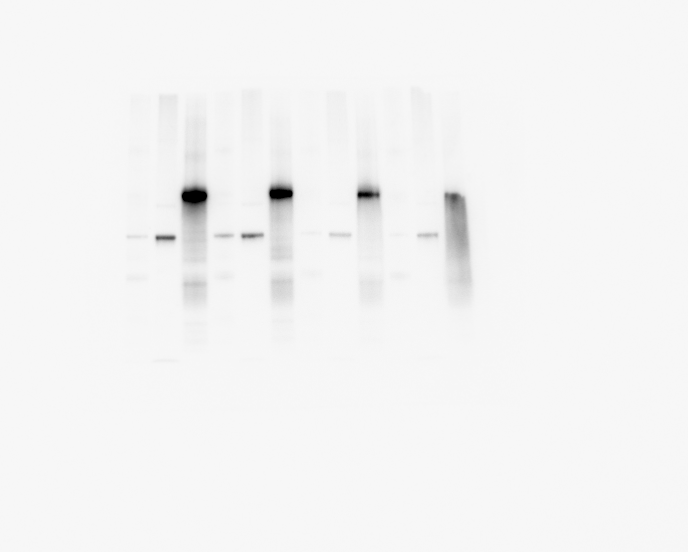

Supplement: Figure 5—source data 1. [file elife-80494-fig5-data1.zip › Fig5/Fig5C/SHORT.tif]

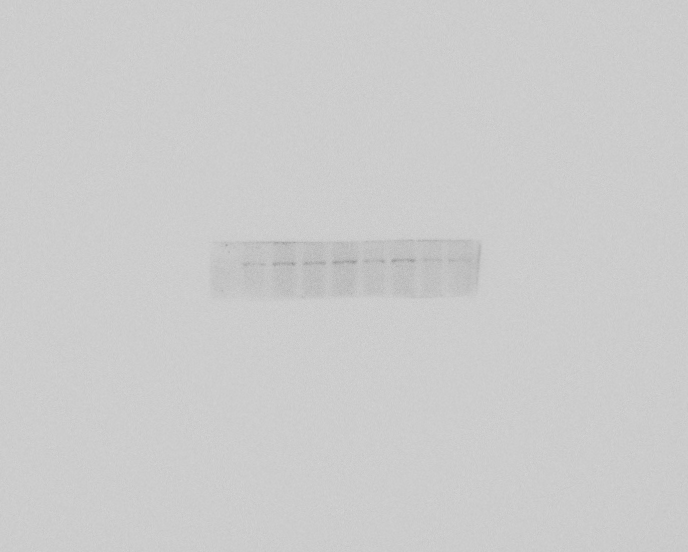

Supplement: Figure 5—source data 1. [file elife-80494-fig5-data1.zip › Fig5/Fig5E/ERA/1.tif]

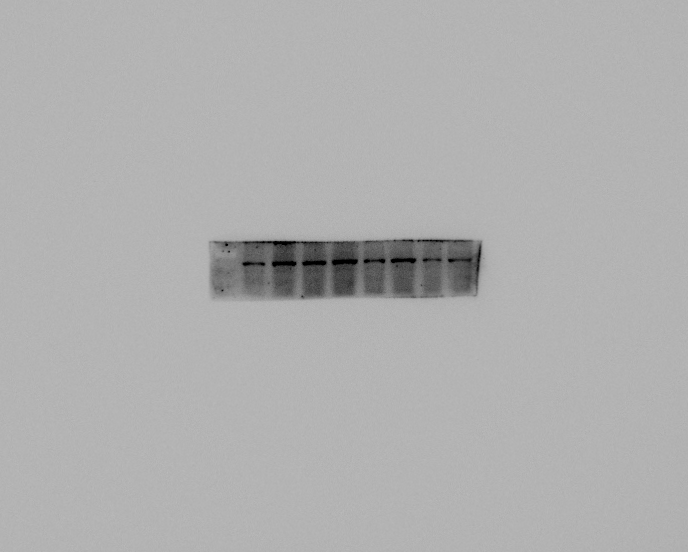

Supplement: Figure 5—source data 1. [file elife-80494-fig5-data1.zip › Fig5/Fig5E/ERA/10.tif]

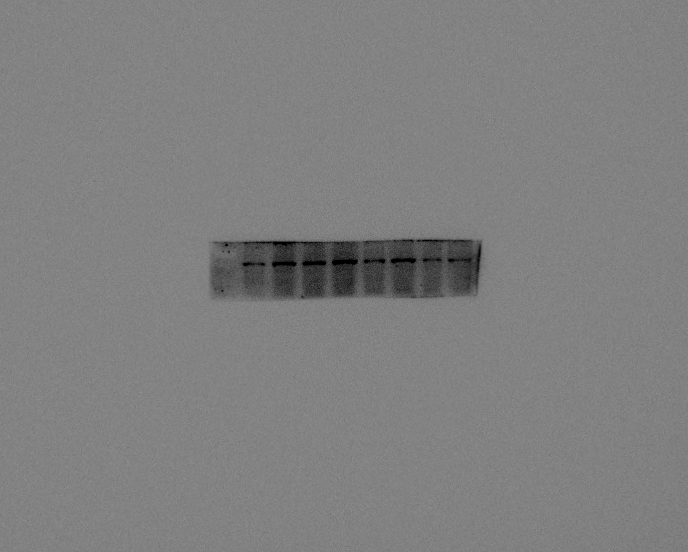

Supplement: Figure 5—source data 1. [file elife-80494-fig5-data1.zip › Fig5/Fig5E/ERA/3.tif]

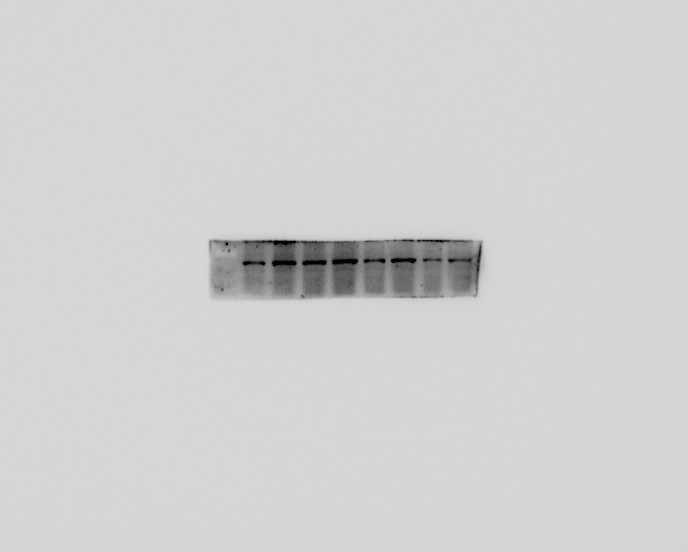

Supplement: Figure 5—source data 1. [file elife-80494-fig5-data1.zip › Fig5/Fig5E/ERA/30.tif]

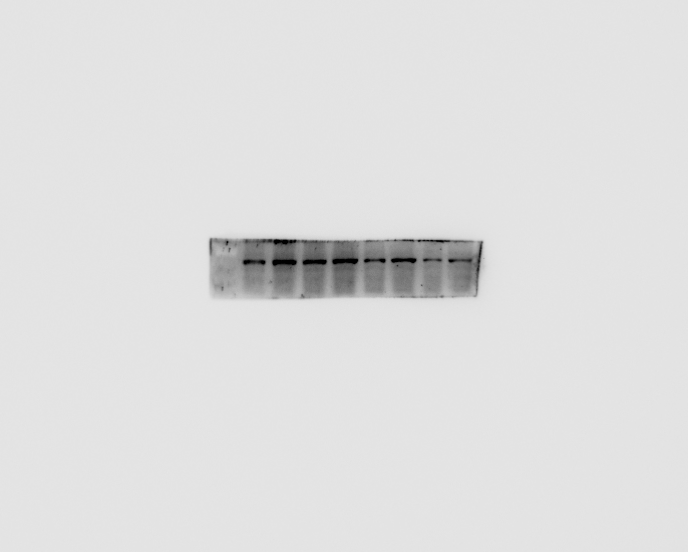

Supplement: Figure 5—source data 1. [file elife-80494-fig5-data1.zip › Fig5/Fig5E/ERA/60.tif]

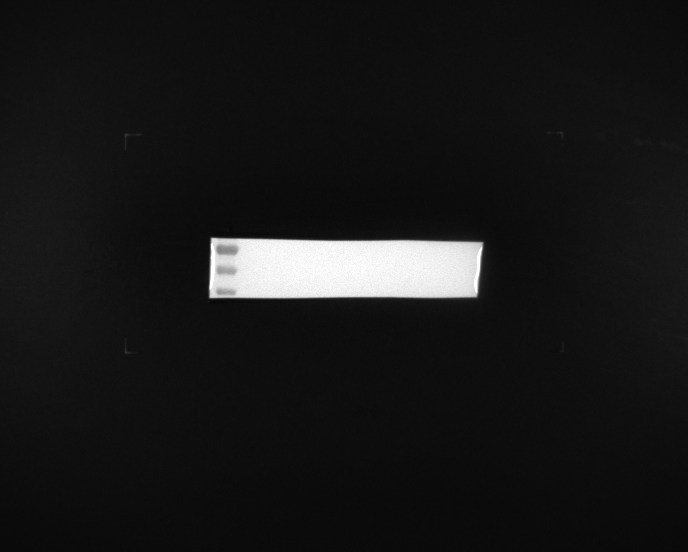

Supplement: Figure 5—source data 1. [file elife-80494-fig5-data1.zip › Fig5/Fig5E/ERA/MK.tif]

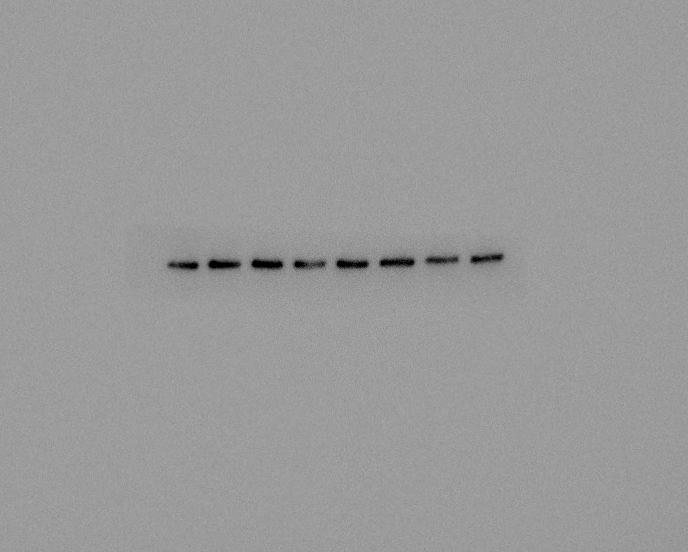

Supplement: Figure 5—source data 1. [file elife-80494-fig5-data1.zip › Fig5/Fig5E/GAPDH/1.tif]

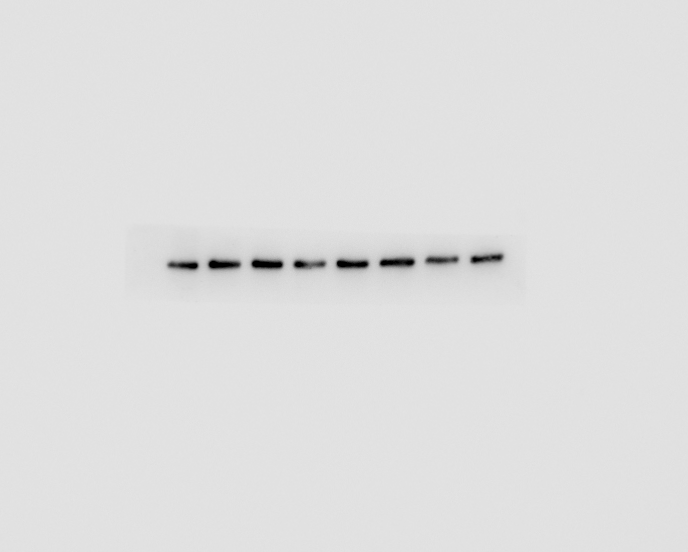

Supplement: Figure 5—source data 1. [file elife-80494-fig5-data1.zip › Fig5/Fig5E/GAPDH/10.tif]

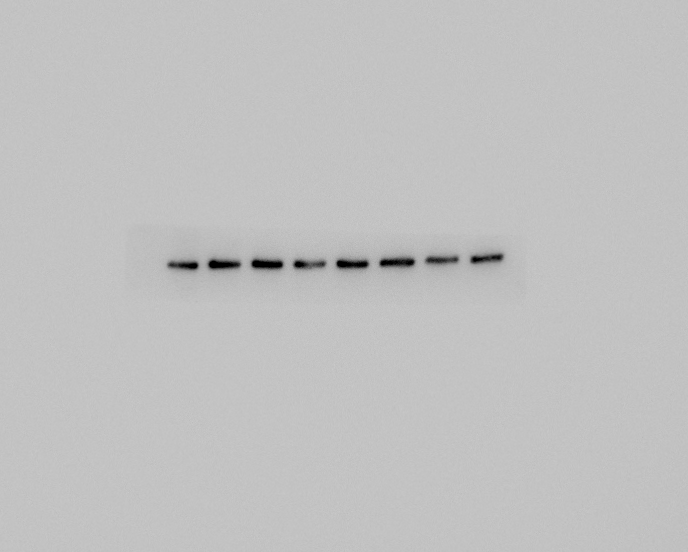

Supplement: Figure 5—source data 1. [file elife-80494-fig5-data1.zip › Fig5/Fig5E/GAPDH/3.tif]

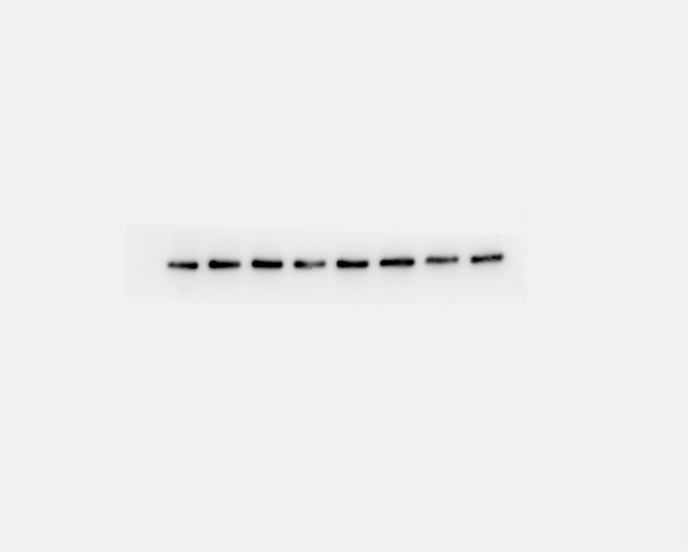

Supplement: Figure 5—source data 1. [file elife-80494-fig5-data1.zip › Fig5/Fig5E/GAPDH/30.tif]

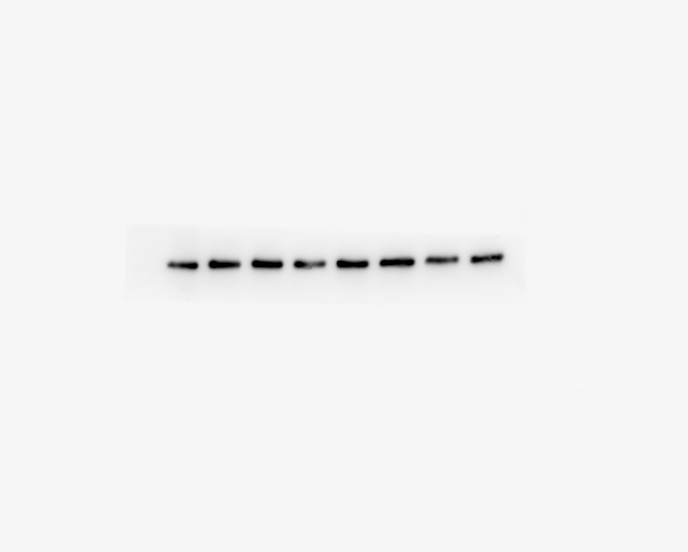

Supplement: Figure 5—source data 1. [file elife-80494-fig5-data1.zip › Fig5/Fig5E/GAPDH/60.tif]

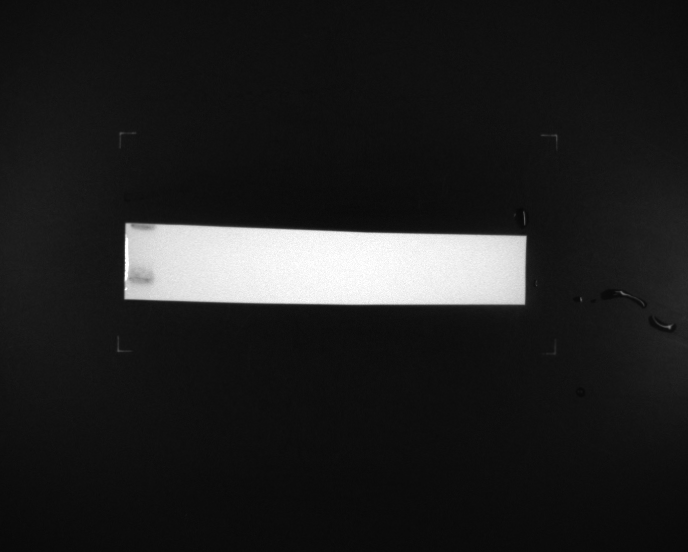

Supplement: Figure 5—source data 1. [file elife-80494-fig5-data1.zip › Fig5/Fig5E/GAPDH/MK.tif]

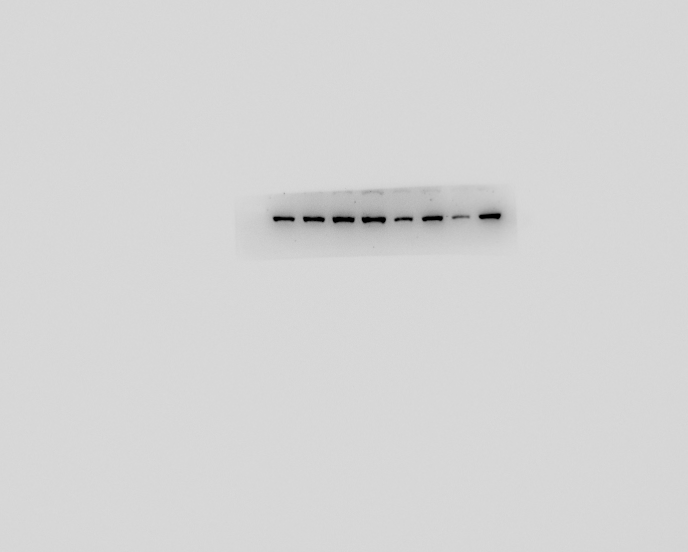

Supplement: Figure 5—source data 1. [file elife-80494-fig5-data1.zip › Fig5/Fig5F/ERa┴/60.tif]

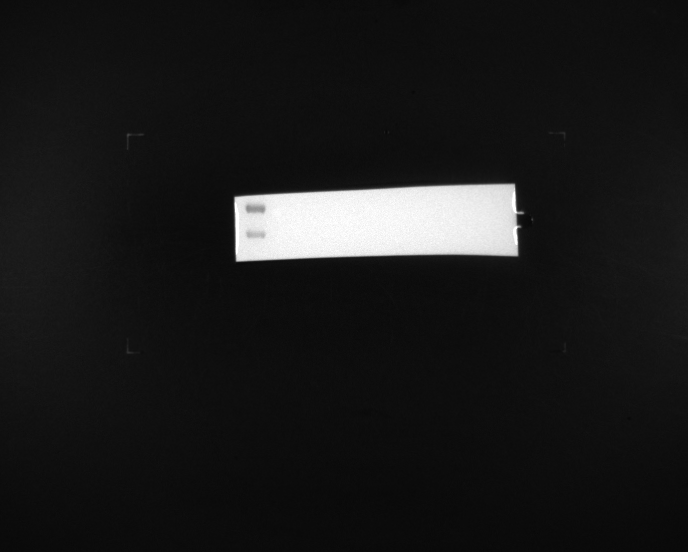

Supplement: Figure 5—source data 1. [file elife-80494-fig5-data1.zip › Fig5/Fig5F/ERa┴/mk.tif]

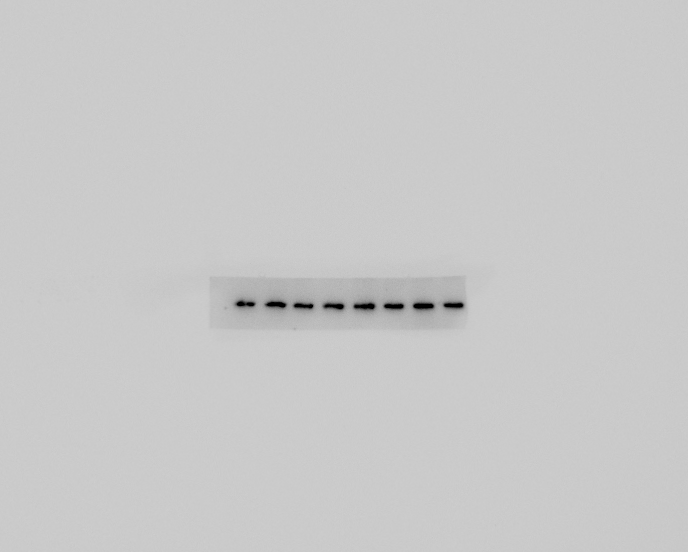

Supplement: Figure 5—source data 1. [file elife-80494-fig5-data1.zip › Fig5/Fig5F/GAPDH/30.tif]

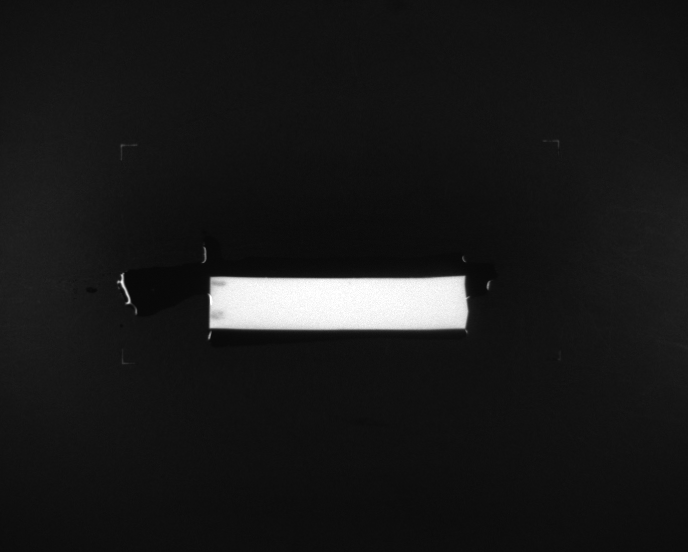

Supplement: Figure 5—source data 1. [file elife-80494-fig5-data1.zip › Fig5/Fig5F/GAPDH/mk.tif]

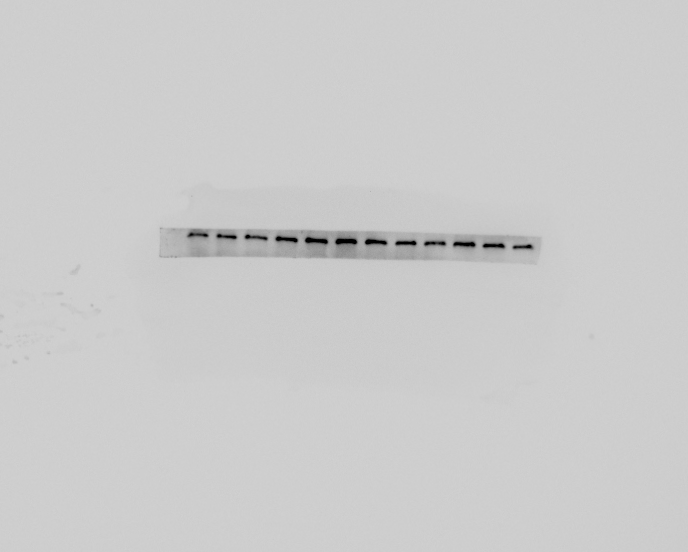

Supplement: Figure 5—source data 1. [file elife-80494-fig5-data1.zip › Fig5/Fig5G/GAPDH/60.tif]

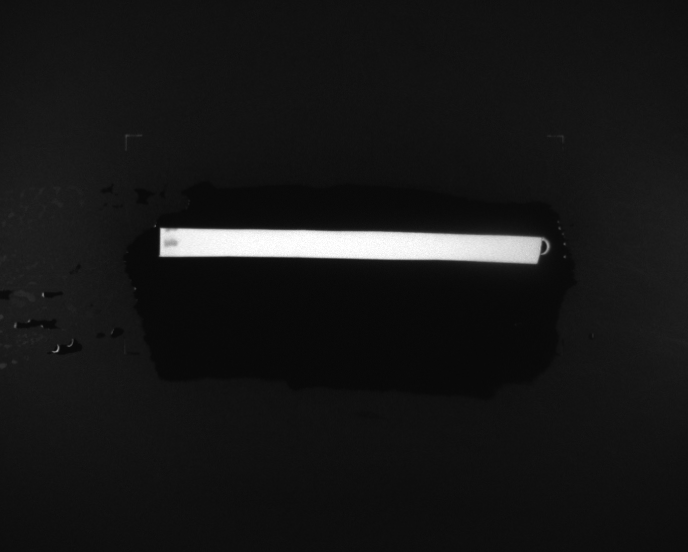

Supplement: Figure 5—source data 1. [file elife-80494-fig5-data1.zip › Fig5/Fig5G/GAPDH/MK.tif]

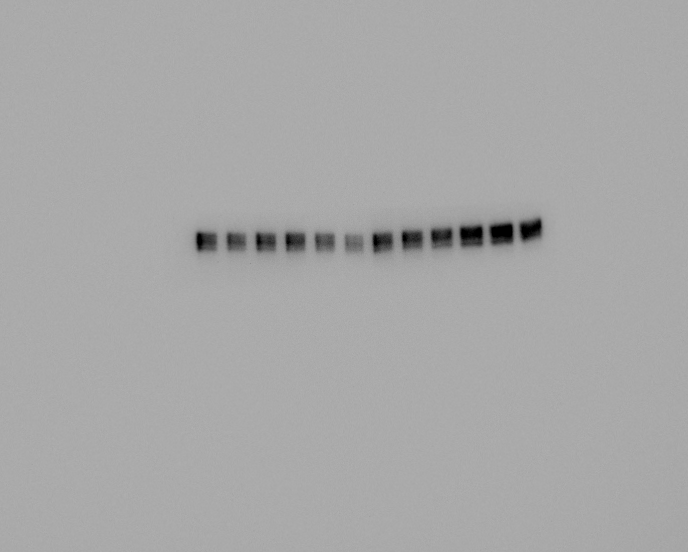

Supplement: Figure 5—source data 1. [file elife-80494-fig5-data1.zip › Fig5/Fig5G/Mdm2/60.tif]

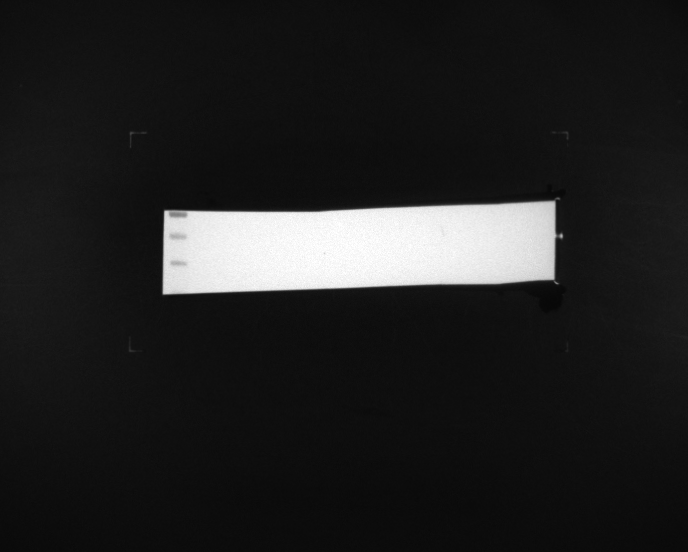

Supplement: Figure 5—source data 1. [file elife-80494-fig5-data1.zip › Fig5/Fig5G/Mdm2/MK.tif]

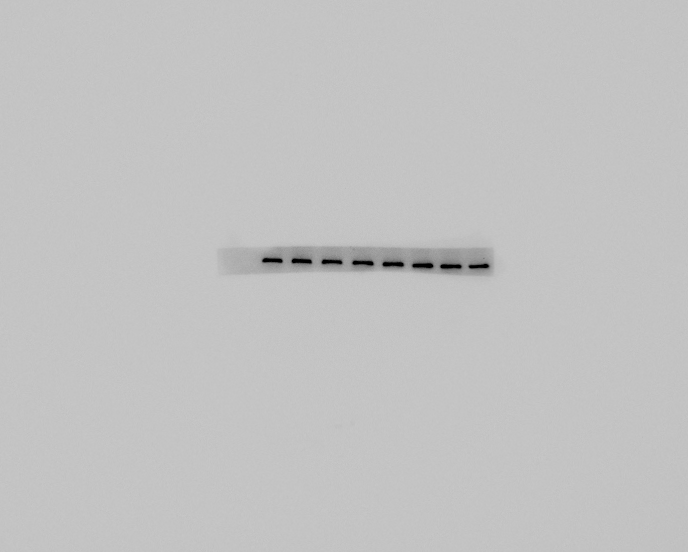

Supplement: Figure 5—source data 1. [file elife-80494-fig5-data1.zip › Fig5/Fig5H/GAPDH/30.tif]

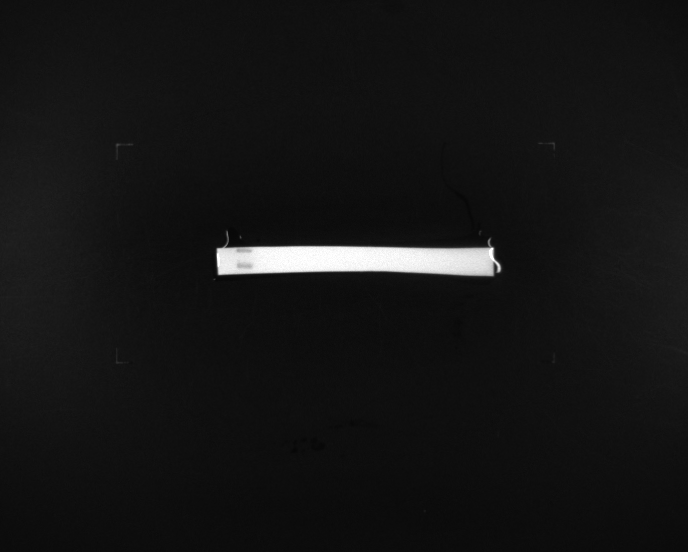

Supplement: Figure 5—source data 1. [file elife-80494-fig5-data1.zip › Fig5/Fig5H/GAPDH/m.tif]

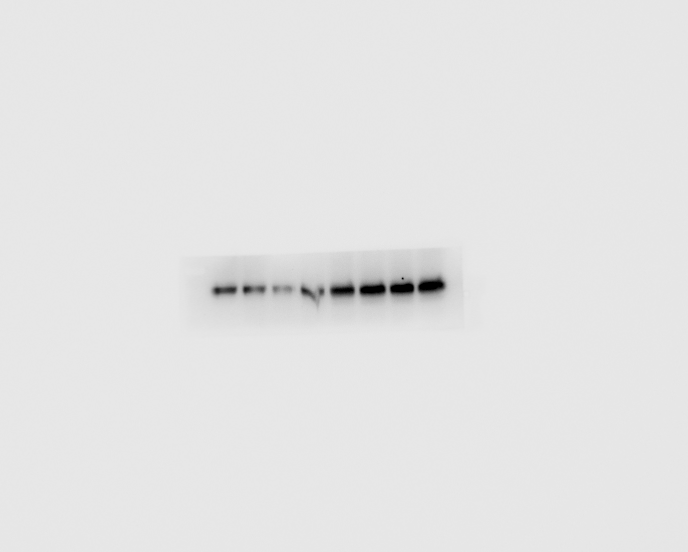

Supplement: Figure 5—source data 1. [file elife-80494-fig5-data1.zip › Fig5/Fig5H/Mdm2/60.tif]

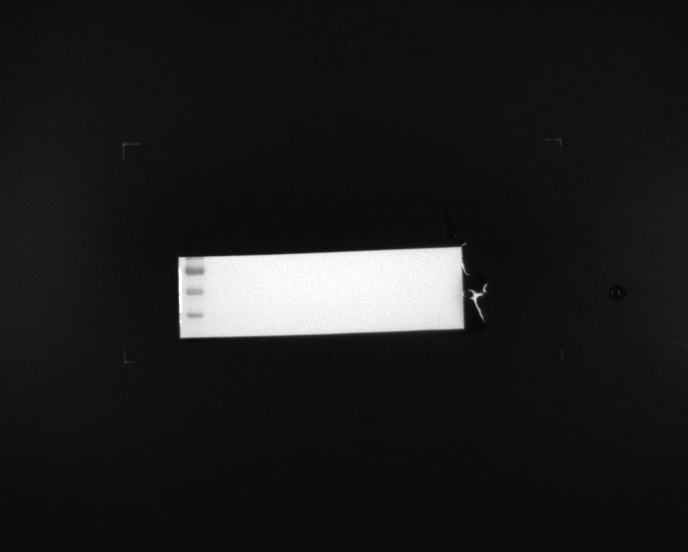

Supplement: Figure 5—source data 1. [file elife-80494-fig5-data1.zip › Fig5/Fig5H/Mdm2/MK.tif]

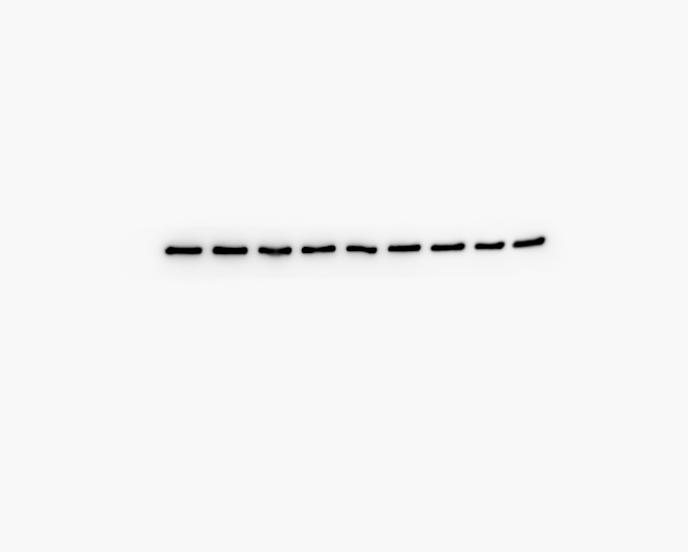

Supplement: Figure 5—figure supplement 1—source data 1. [file elife-80494-fig5-figsupp1-data1.zip › FigS3/SFig2A/GAPDH/30.tif]

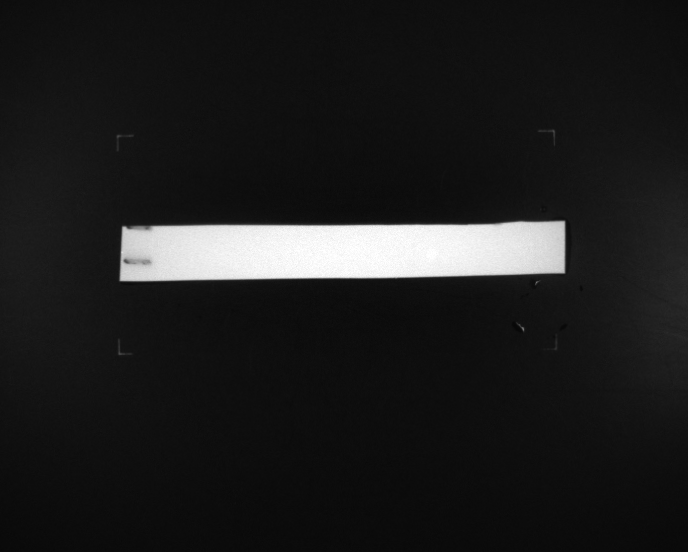

Supplement: Figure 5—figure supplement 1—source data 1. [file elife-80494-fig5-figsupp1-data1.zip › FigS3/SFig2A/GAPDH/MK.tif]

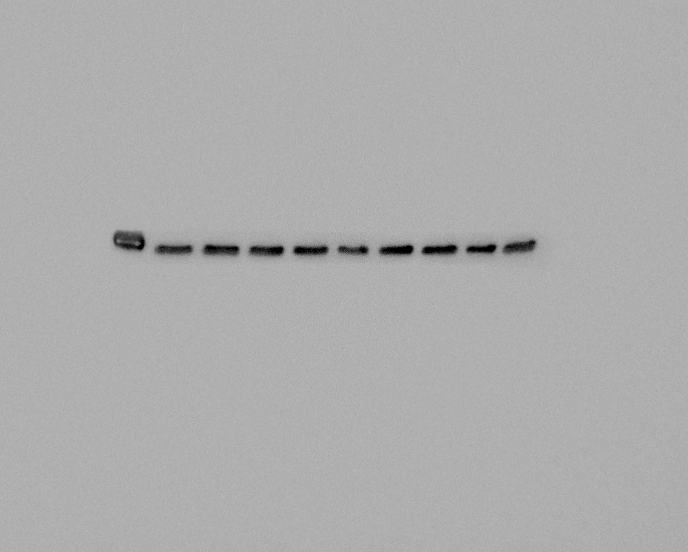

Supplement: Figure 5—figure supplement 1—source data 1. [file elife-80494-fig5-figsupp1-data1.zip › FigS3/SFig2A/SOD2/1.tif]

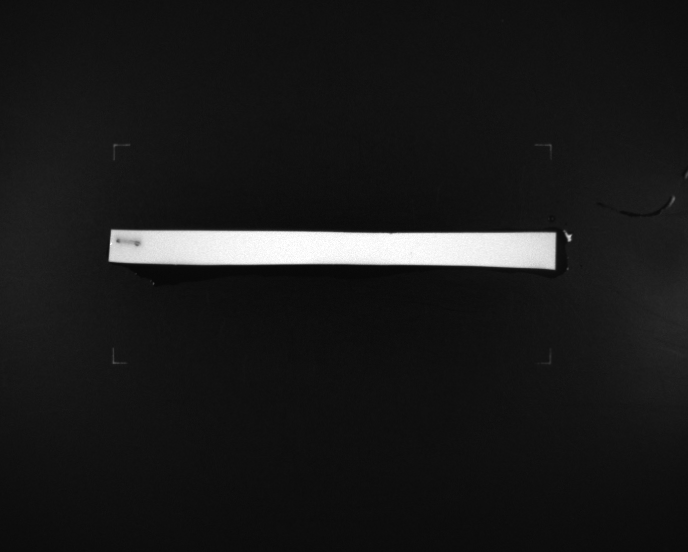

Supplement: Figure 5—figure supplement 1—source data 1. [file elife-80494-fig5-figsupp1-data1.zip › FigS3/SFig2A/SOD2/mk.tif]

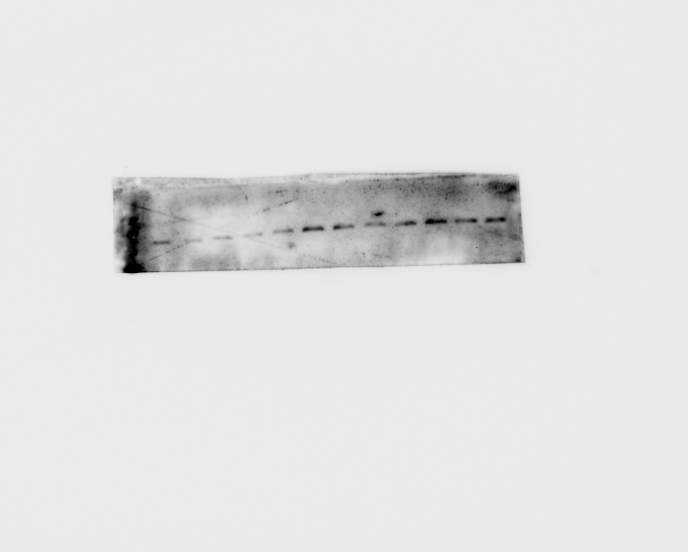

Supplement: Figure 5—figure supplement 2—source data 1. [file elife-80494-fig5-figsupp2-data1.zip › FigS4/SFig3A/AHR/60.tif]

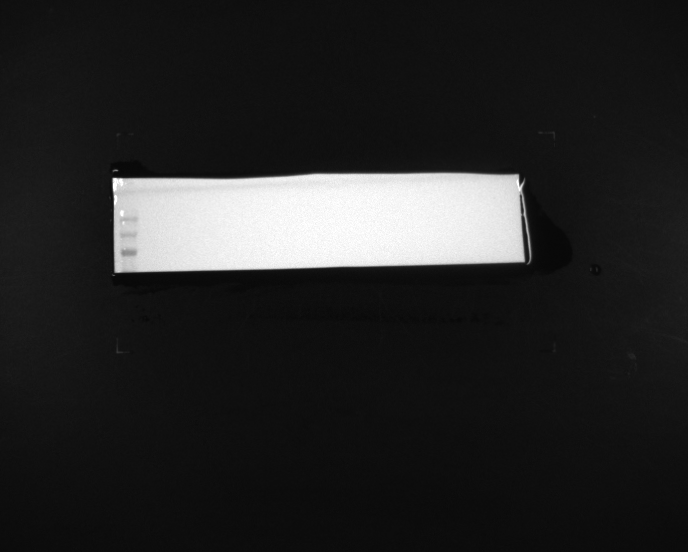

Supplement: Figure 5—figure supplement 2—source data 1. [file elife-80494-fig5-figsupp2-data1.zip › FigS4/SFig3A/AHR/m k.tif]

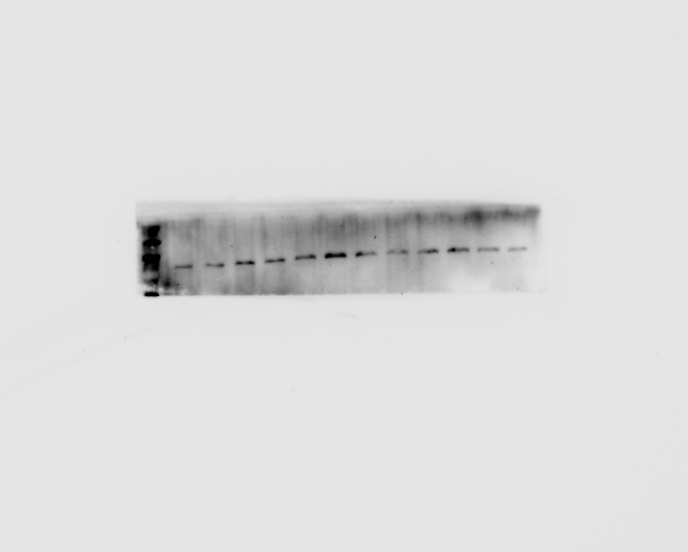

Supplement: Figure 5—figure supplement 2—source data 1. [file elife-80494-fig5-figsupp2-data1.zip › FigS4/SFig3A/BRCA1/60.tif]

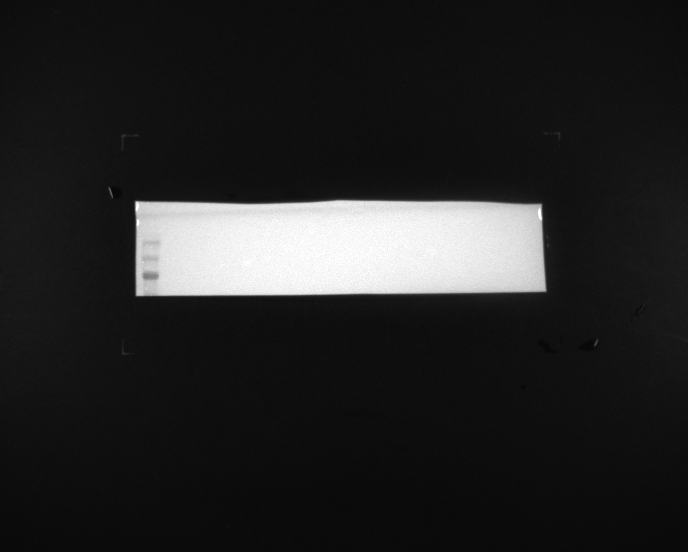

Supplement: Figure 5—figure supplement 2—source data 1. [file elife-80494-fig5-figsupp2-data1.zip › FigS4/SFig3A/BRCA1/MK.tif]

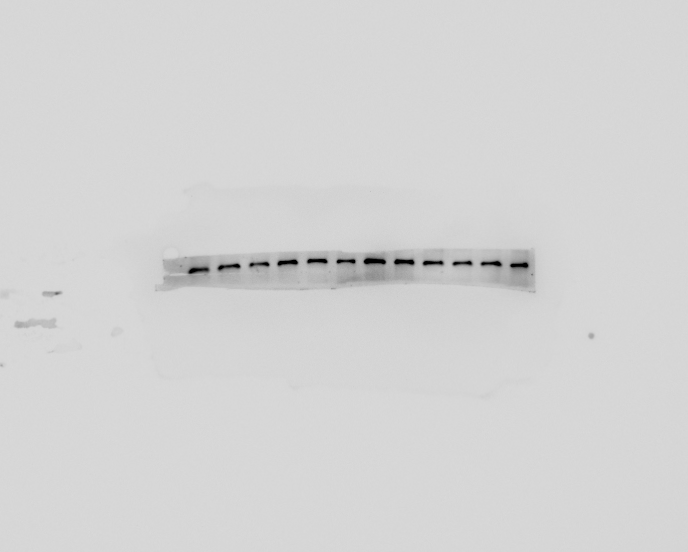

Supplement: Figure 5—figure supplement 2—source data 1. [file elife-80494-fig5-figsupp2-data1.zip › FigS4/SFig3A/GAPDH/60.tif]

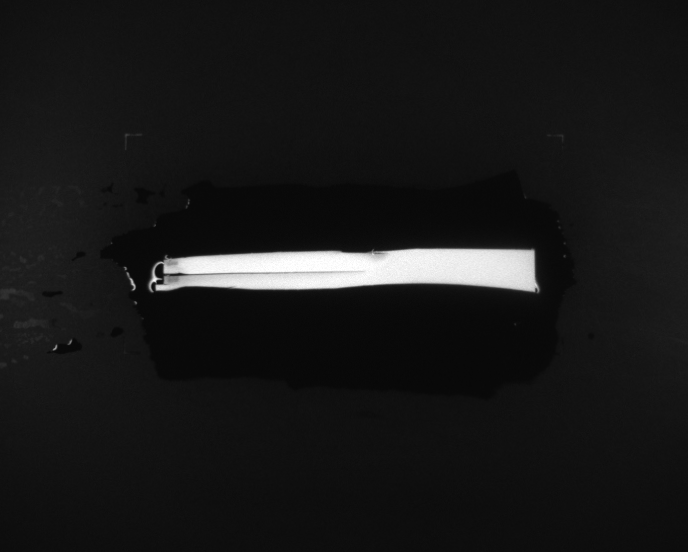

Supplement: Figure 5—figure supplement 2—source data 1. [file elife-80494-fig5-figsupp2-data1.zip › FigS4/SFig3A/GAPDH/MK.tif]

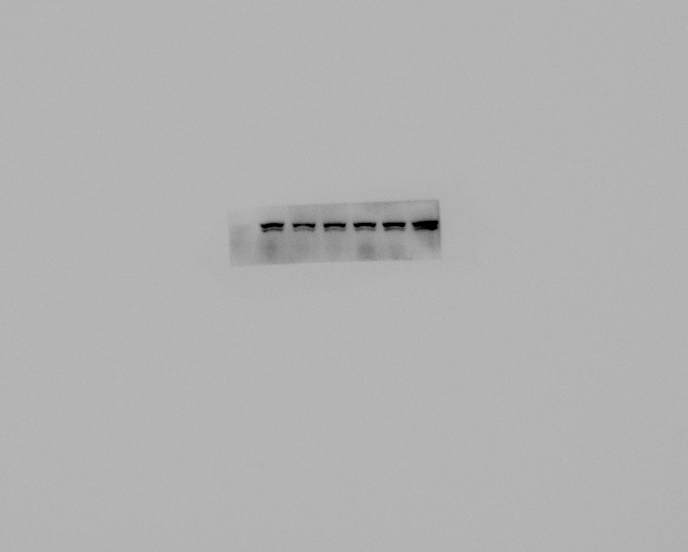

Supplement: Figure 5—figure supplement 2—source data 1. [file elife-80494-fig5-figsupp2-data1.zip › FigS4/SFig3B/AHR/30.tif]

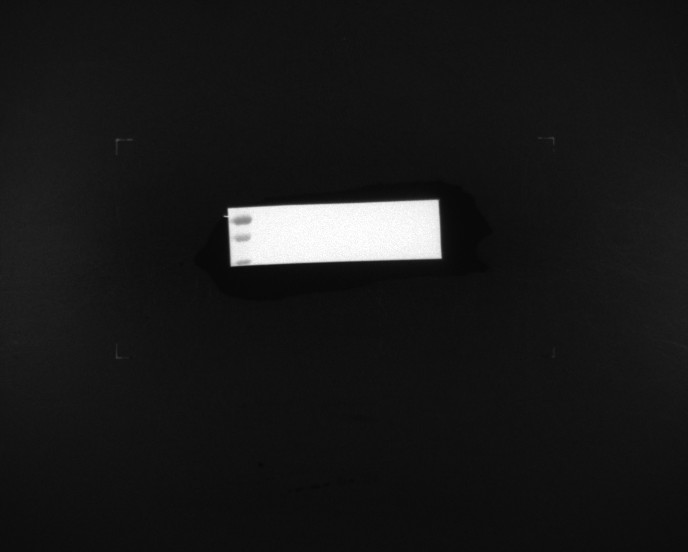

Supplement: Figure 5—figure supplement 2—source data 1. [file elife-80494-fig5-figsupp2-data1.zip › FigS4/SFig3B/AHR/MK.tif]

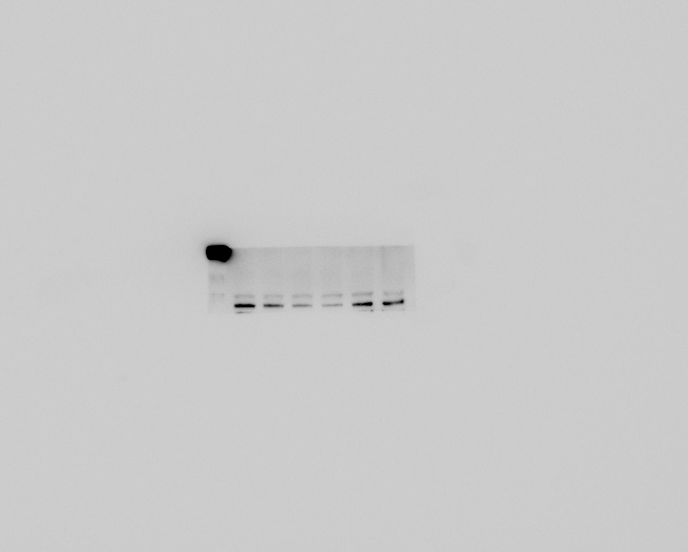

Supplement: Figure 5—figure supplement 2—source data 1. [file elife-80494-fig5-figsupp2-data1.zip › FigS4/SFig3B/BRCA1/30.tif]

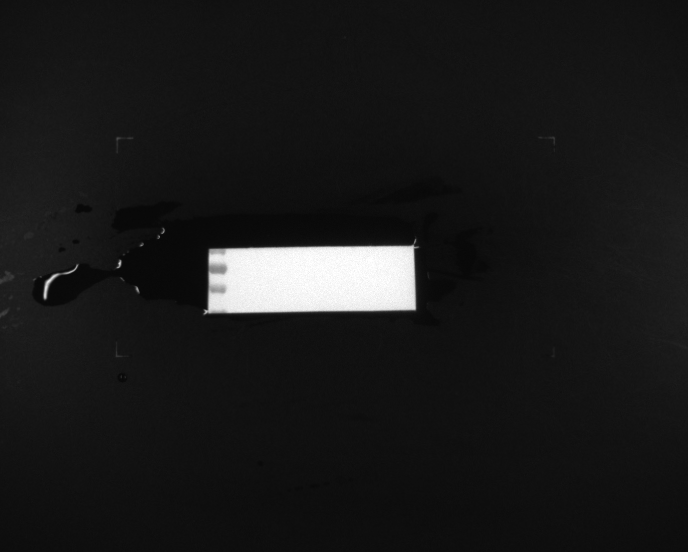

Supplement: Figure 5—figure supplement 2—source data 1. [file elife-80494-fig5-figsupp2-data1.zip › FigS4/SFig3B/BRCA1/MK.tif]

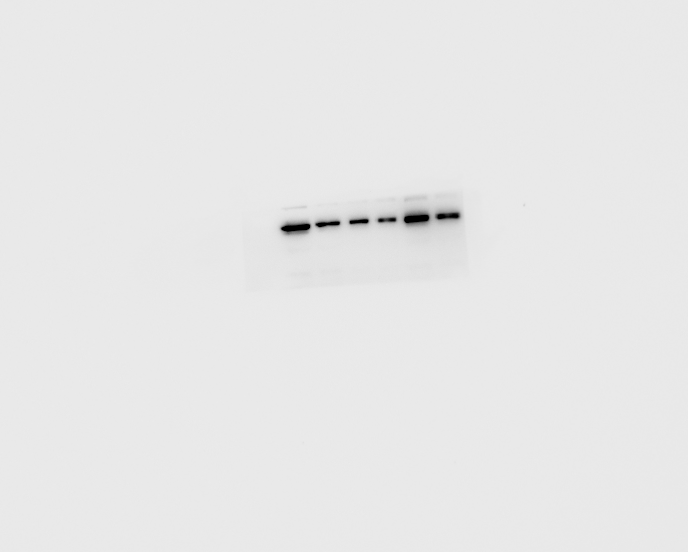

Supplement: Figure 5—figure supplement 2—source data 1. [file elife-80494-fig5-figsupp2-data1.zip › FigS4/SFig3B/ERa┴/30.tif]

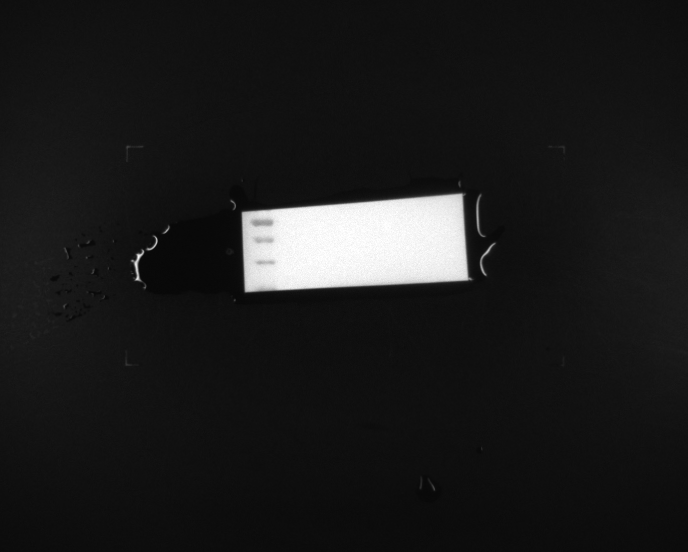

Supplement: Figure 5—figure supplement 2—source data 1. [file elife-80494-fig5-figsupp2-data1.zip › FigS4/SFig3B/ERa┴/MK.tif]

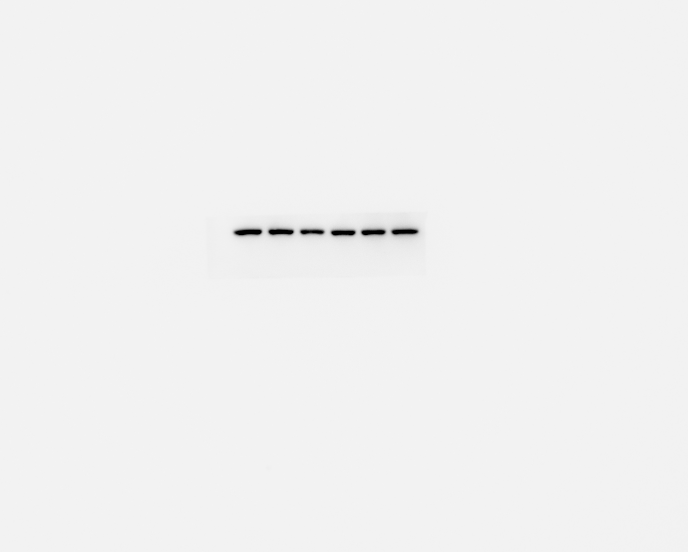

Supplement: Figure 5—figure supplement 2—source data 1. [file elife-80494-fig5-figsupp2-data1.zip › FigS4/SFig3B/GAPDH/10.tif]

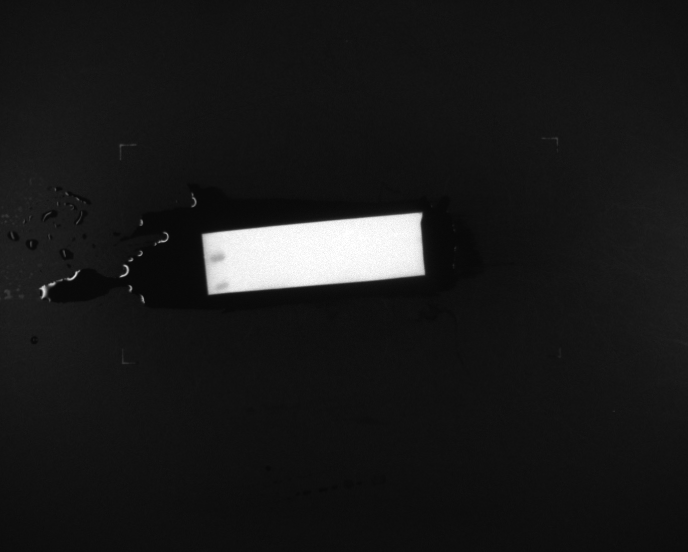

Supplement: Figure 5—figure supplement 2—source data 1. [file elife-80494-fig5-figsupp2-data1.zip › FigS4/SFig3B/GAPDH/MK.tif]

sham

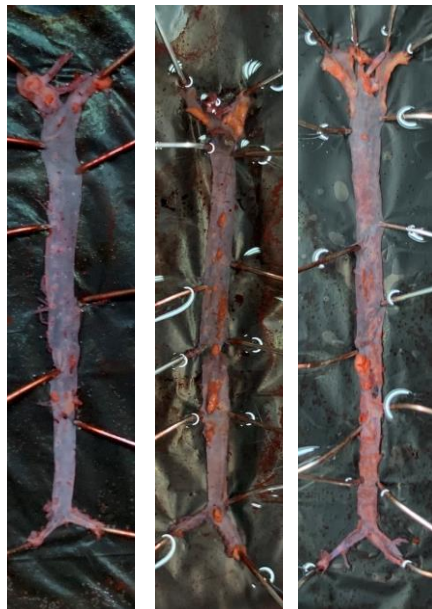

E<sub>2</sub>

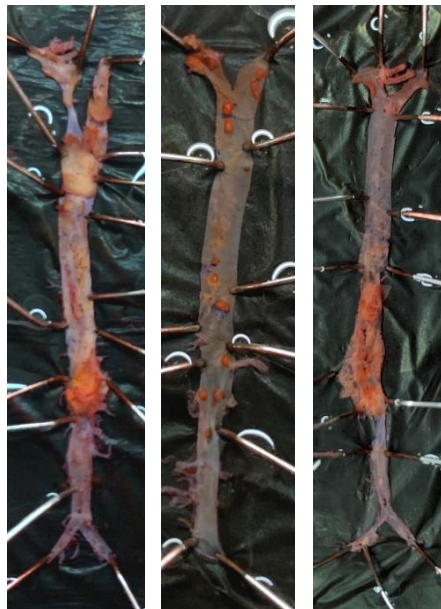

DFP

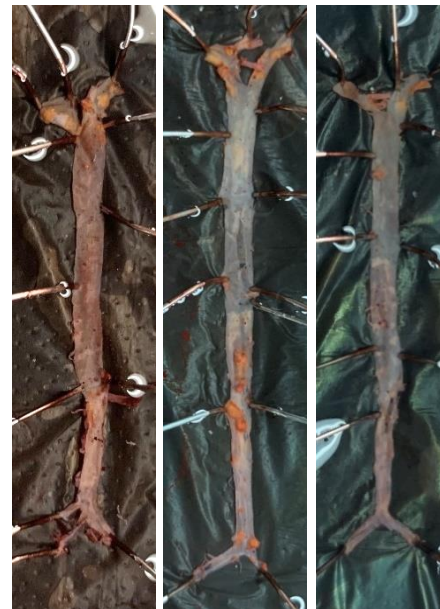

E<sub>2</sub>+DFP

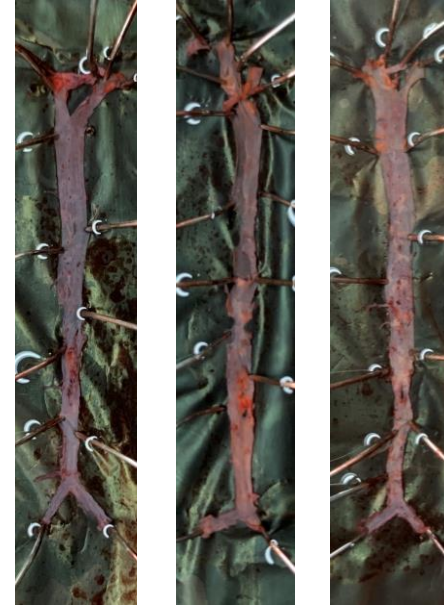

Supplement: Figure 6—source data 1. [file elife-80494-fig6-data1.zip › Fig6/Fig6B/FIG6B.pdf]

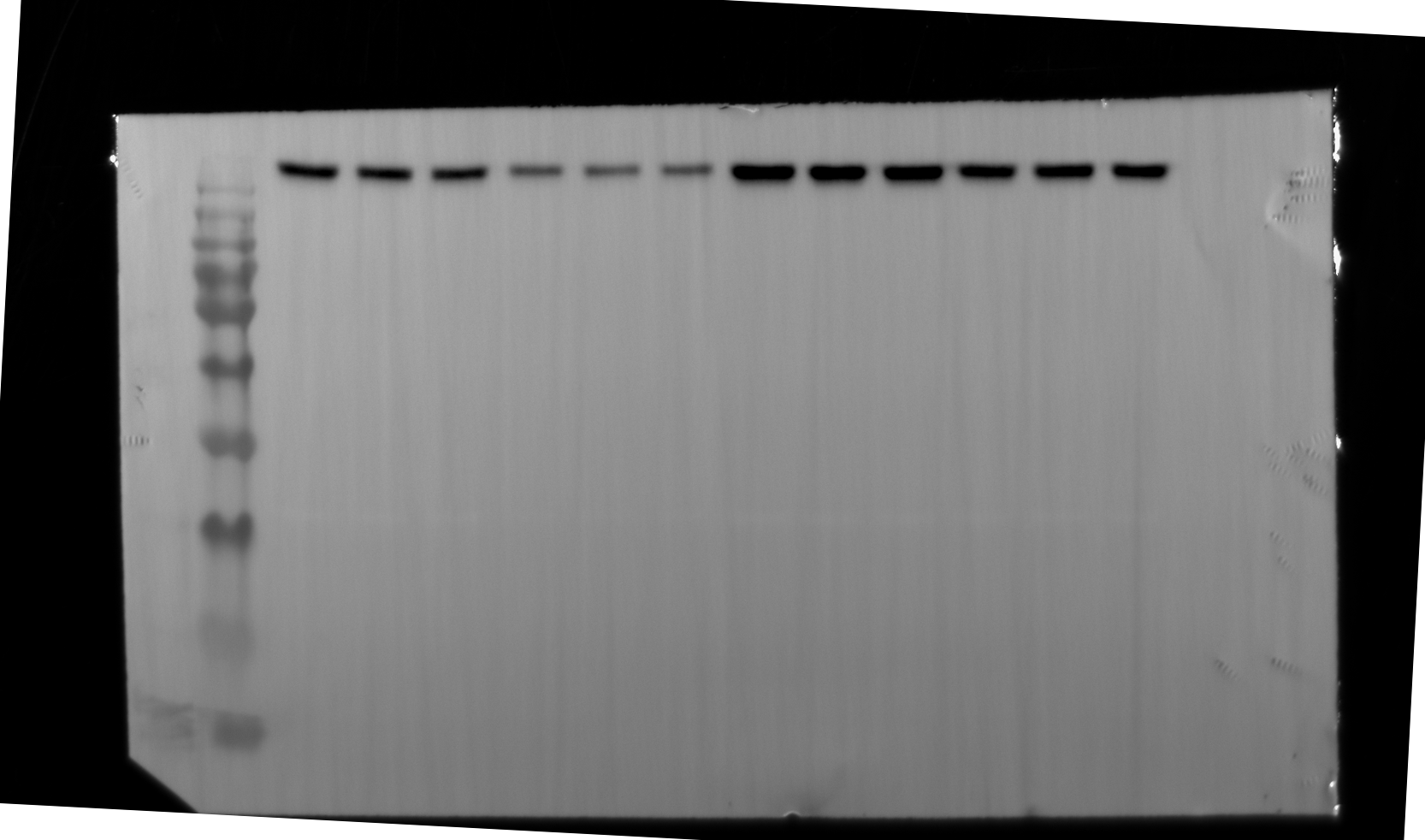

Supplement: Figure 6—source data 1. [file elife-80494-fig6-data1.zip › Fig6/Fig6G/ABCA1.tif]

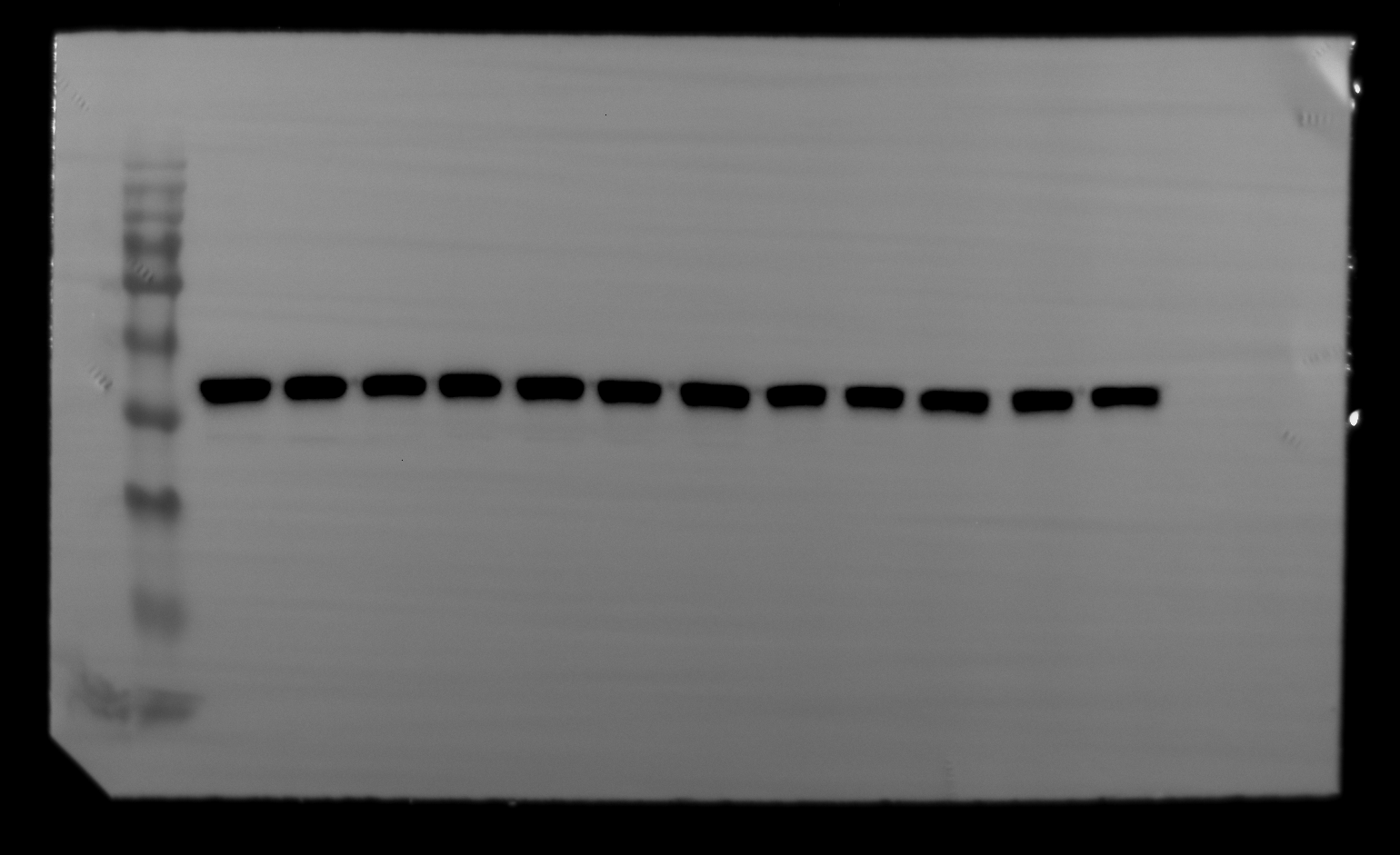

Supplement: Figure 6—source data 1. [file elife-80494-fig6-data1.zip › Fig6/Fig6G/ACTIN.tif]

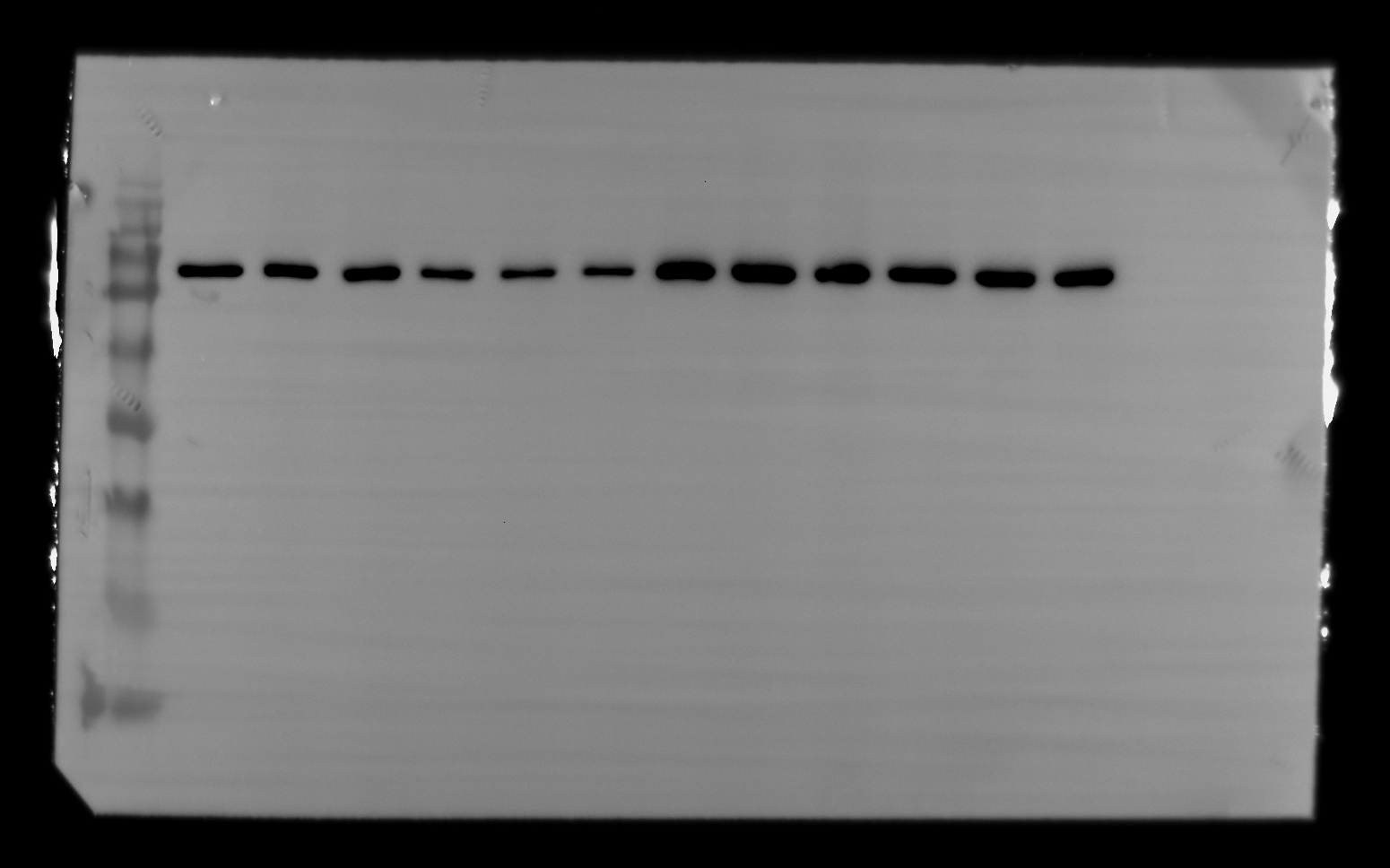

Supplement: Figure 6—source data 1. [file elife-80494-fig6-data1.zip › Fig6/Fig6G/ERa┴.tif]

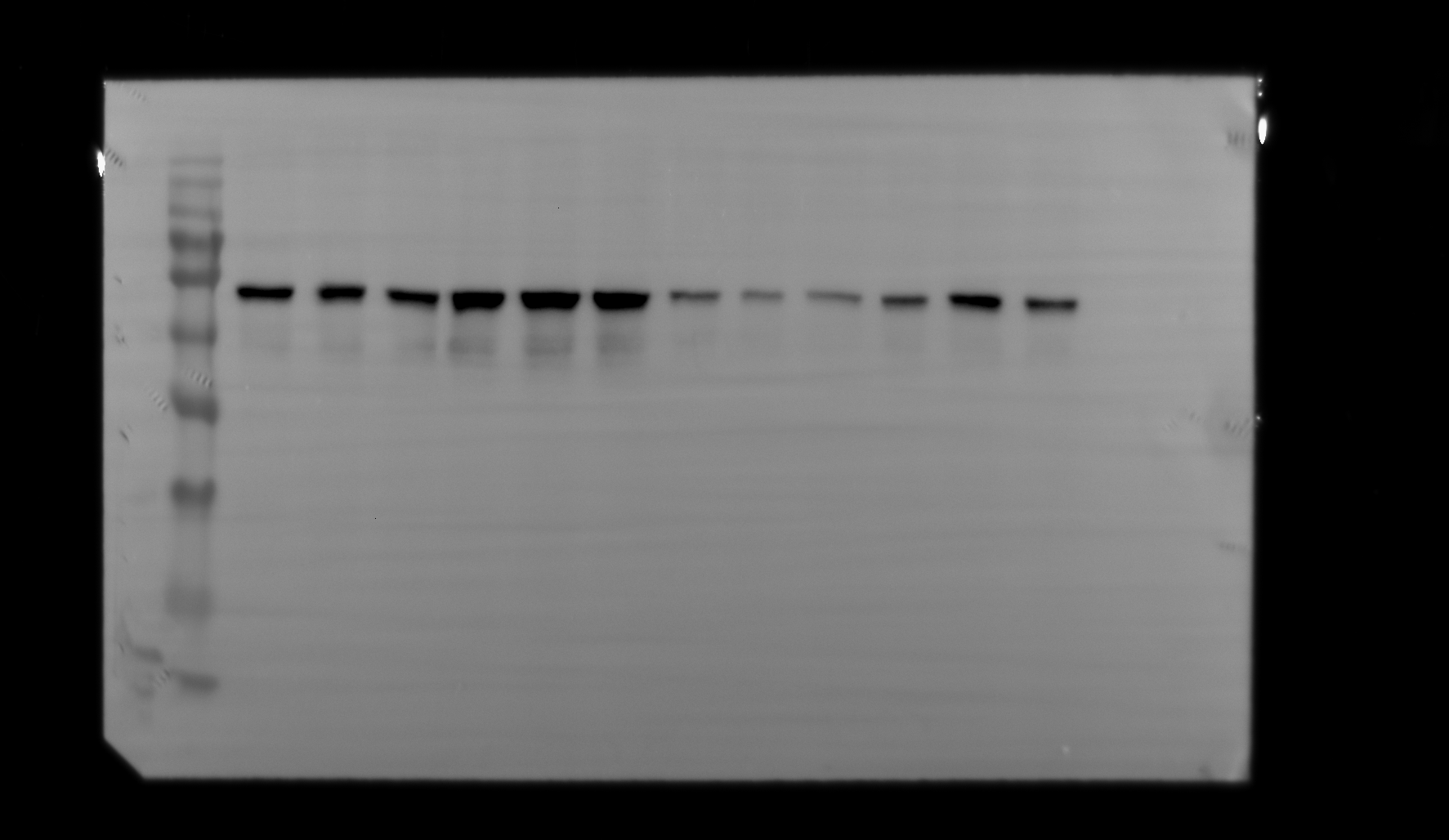

Supplement: Figure 6—source data 1. [file elife-80494-fig6-data1.zip › Fig6/Fig6G/MDM2.tif]

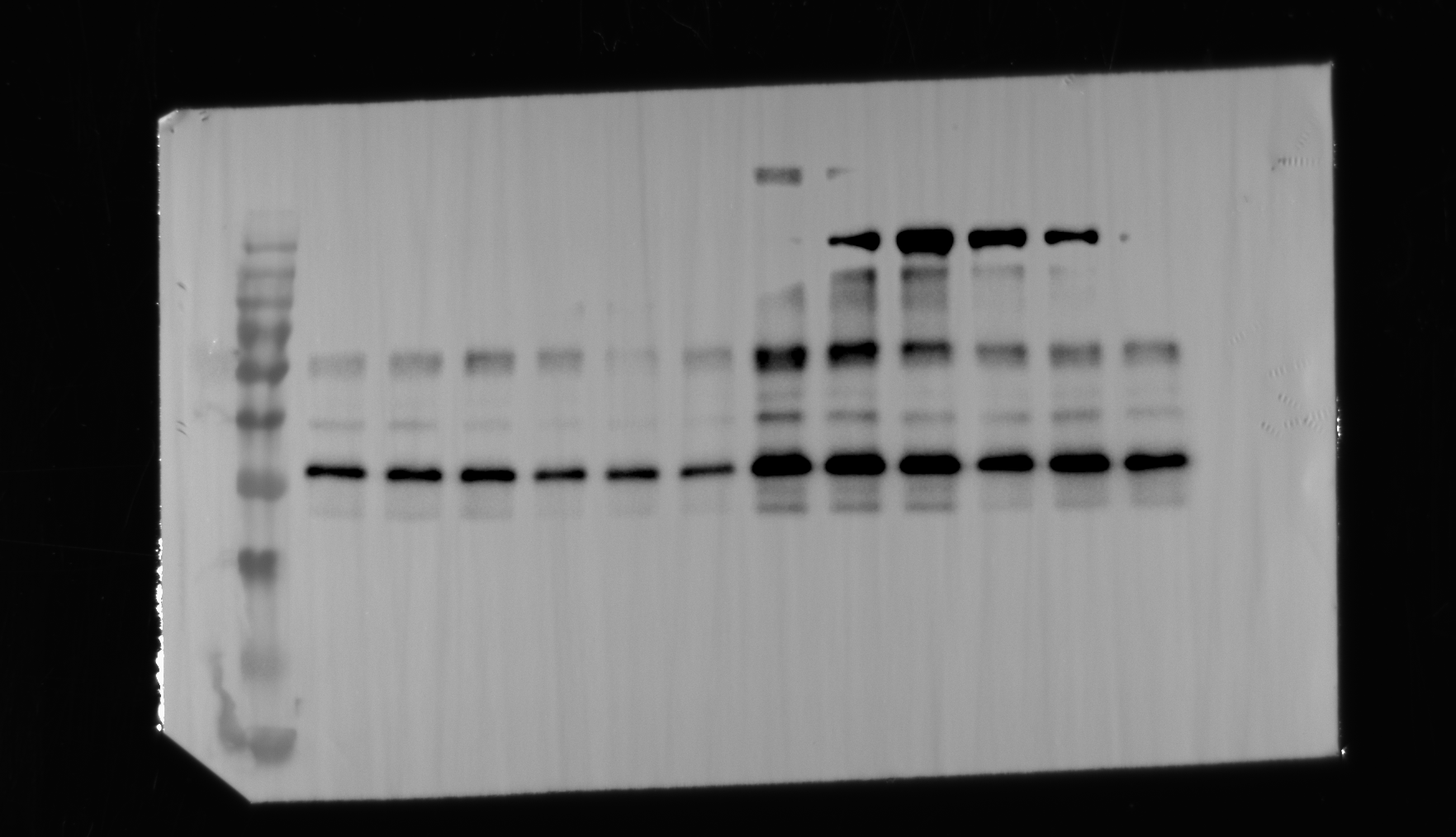

Supplement: Figure 6—source data 1. [file elife-80494-fig6-data1.zip › Fig6/Fig6G/VEGF.tif]
